# Preformed fibrils of α-synuclein rapidly activate LRRK2 on early endosomes, driving Rab5 phosphorylation and disrupting endolysosomal and synaptic function

**DOI:** 10.1038/s41531-026-01382-z

**Published:** 2026-05-12

**Authors:** Xinxin Zuo, Zeyu Chen, Xu-Qiao Chen, Dongxu Guan, Peter X. Shaw, Aaron Johnstone, Ann Becker, Utpal Das, William C. Mobley

**Affiliations:** 1 Department of Neurosciences, University of California San Diego, La Jolla, CA USA; 2 Department of Medicine, University of California San Diego, La Jolla, CA USA; 3 Shiley Eye Institute, University of California San Diego, La Jolla, CA USA

**Keywords:** Cell biology, Molecular biology, Neuroscience

## Abstract

Parkinson’s disease (PD) is characterized by α-synuclein aggregation and perturbation of the endolysosomal network (ELN), yet the molecular mechanisms linking α-synuclein pathology to neuronal dysfunction remain unclear. Here we report that treatment of mouse cortical neurons with α-synuclein preformed fibrils (PFFs) alters lysosomal composition and impairs lysosomal function, coupled with extensive chromatin remodeling and transcriptional reprogramming, including suppression of neuronal gene networks and activation of senescence-like programs. Mechanistically, these changes are associated with rapid recruitment and activation of the PD-associated kinase LRRK2 on early endosomes, where it phosphorylates Rab5, a key early endosomal GTPase, leading to remodeling of the Rab5 interactome, altered effector engagement, and endosomal dyshomeostasis. Pharmacological inhibition of LRRK2 with MLi-2 restores Rab5 activity, lysosomal function, chromatin accessibility, gene expression, and neuronal excitability. Knockdown of Rab5 partially rescues chromatin changes, supporting its role as a downstream effector. These findings identify LRRK2 hyperactivation and the LRRK2-Rab5 axis as key mediators of PFF-induced neuronal dysfunction, highlighting early endosomes as a central platform linking endolysosomal disruption to nuclear responses and offering potential targets for therapeutic intervention in PD.

## Introduction

A key characteristic of neurodegenerative diseases is accumulation of aggregates of specific misfolded proteins and disruption of cellular homeostasis, ultimately leading to progressive neuronal loss^1^. In Parkinson’s disease (PD), this process is marked by degeneration of midbrain dopaminergic neurons and formation therein of Lewy bodies (LBs), primarily composed of fibrillar deposits of α-synuclein as well as entrapped organelles^2,3^. Under physiological conditions, α-synuclein localizes to presynaptic terminals, where it modulates neurotransmitter release^4,5^. The events that disrupt the normal function of α-synuclein and result in its misfolding and deposition are not well understood but are viewed as essential to the seeding and propagation of α-synuclein, which drives the progression of PD pathogenesis. Studies exploring the mechanism employ in vitro systems in which preformed fibrils (PFFs) are added to primary neuronal cultures, accelerating misfolding and aggregation of endogenous α-synuclein, and leading to synaptic dysfunction and neuronal death^6,7^. Intrastriatal injection of PFFs impairs dopamine release and leads to degeneration of dopaminergic neurons in the substantia nigra^8,9^. These findings underscore the utility of PFFs to model the pathogenetic process induced by aggregated α-synuclein and offer insights into degenerative events characteristic of PD.

The endolysosomal network (ELN) is a highly dynamic and interconnected system of membrane-bound vesicles and tubules, transitioning through early endosomes (EEs), recycling endosomes, late endosomes, and lysosomes. Autophagosomes also play a role by delivering intracellular material to lysosomes for autophagic degradation. The ELN orchestrates essential cellular functions such as nutrient uptake via endocytosis, transmission of trophic signals, vesicle recycling, intracellular protein trafficking, and maintenance of proteostasis and organelle integrity^10^. In neurons, these processes are particularly vulnerable due to their complex, polarized architecture, which challenges the efficient transport of trophic signals to the soma and the delivery of degradation-bound cargoes to lysosomes^11,12^. This vulnerability is further amplified in PD, especially in dopaminergic neurons of the substantia nigra pars compacta (SNpc), which exhibit selective degeneration^13^.

Lysosomes serve as the central degradation hub for proteins and organelles, maintaining cellular homeostasis through recycling and turnover. Lysosomal biogenesis is transcriptionally regulated by the MiT/TFE family of transcription factors, including MITF, TFEB, TFE3, and TFEC^14^. These factors bind to the Coordinated Lysosomal Expression and Regulation (CLEAR) element, a palindromic 10-base pair (bp) sequence (GTCACGTGAC) found in the promoter regions of many lysosomal genes, and drive their expression^15^. Post-translational modifications of MiT/TFE factors regulate their nuclear translocation, DNA binding, and subsequent activation of genes required for autophagy and lysosomal biogenesis^16^. Growing genetic evidence implicates lysosomal dysfunction as a significant contributor to PD pathogenesis, with several lysosomal regulatory genes identified as PD risk factors^17–19^. Studies of the ELN have largely focused on changes in lysosomal structure and function^20,21^. However, the mechanisms are not well characterized. Understanding ELN dysregulation in PD requires further investigation of upstream endosomal compartments.

Leucine-rich repeat kinase 2 (LRRK2) is a multidomain protein kinase; mutations in *LRRK2* represent the most common genetic cause of familial PD. Disease-linked LRRK2 mutations typically enhance its kinase activity, though how this leads to PD pathogenesis remains an open question. One clue relates to LRRK2-mediated phosphorylation of specific Rab GTPases, including Rabs 8, 10 and 12^22–24^. Phosphorylation of these Rab proteins disrupt their interaction with key regulators, such as guanine nucleotide exchange factors (GEFs), GTPase-activating proteins (GAPs), guanine nucleotide dissociation inhibitors (GDIs)^25^. Impaired GDIs engagement compromises Rab cycling between active and inactive states and alters effectors interactions, ultimately perturbing Rab function^26^. The net effect of these changes is compromised trafficking and dysregulation of ELN compartments. Recent studies have shown that LRRK2 is recruited to damaged lysosomes, a process amplified by PD-associated mutations such as G2019S^27^. Artificially targeting LRRK2 to lysosomes or Golgi membranes is sufficient to activate its signaling, suggesting that LRRK2 kinase activity is tightly coupled to its membrane localization^28^. However, little is known whether or not and how LRRK2 may be implicated in the function and dynamics of upstream ELN compartments such as early endosomes. Rab5, a master regulator of early endosome biogenesis and trafficking^29^, has been proposed as a potential LRRK2 substrate, but existing evidence remains inconclusive. Proteomic analysis have identified Rab5 phosphorylation in cells overexpressing LRRK2^30^, and in vitro kinase assay revealed only weak phosphorylation by LRRK2^31^. Given the central role of Rab5 in early endosomal dynamics, even subtle changes in its regulation could have profound effects on ELN homeostasis. We therefore speculated that LRRK2-dependent modulation of Rab5 may represent an unrecognized mechanism contributing to early ELN dysfunction in PD.

Herein, we undertook studies to examine molecular and cellular events induced by PFF treatment in mouse cortical neurons, with a focus on ELN regulation. PFF treatment disrupted lysosomal protein composition and compromised lysosomal function and homeostasis. These organelle-level alterations were accompanied by transcriptional reprogramming and chromatin remodeling, engaging multiple cellular pathways including those associated with neuronal senescence. To investigate upstream mechanisms that drive these phenotypes, we examined the role of LRRK2 and Rab GTPases. We found that LRRK2 was rapidly recruited to early endosomes, where it phosphorylated Rab5, leading to remodeling of the Rab5 interactome, altered effector engagement, and disruption of endocytic trafficking. Notably, inhibiting LRRK2 activity or reducing the levels of Rab5 reversed several PFF-induced phenotypes. These findings identify early endosomes as a key platform for propagating LRRK2-mediated neuronal dysfunction. By delineating early pathogenic mechanisms in this model, we provide insights into the pathophysiology of PD and highlight potential therapeutic targets to prevent or delay disease progression.

## Results

### Disruption of lysosomal protein composition and function by PFF

**Fig. 1:**
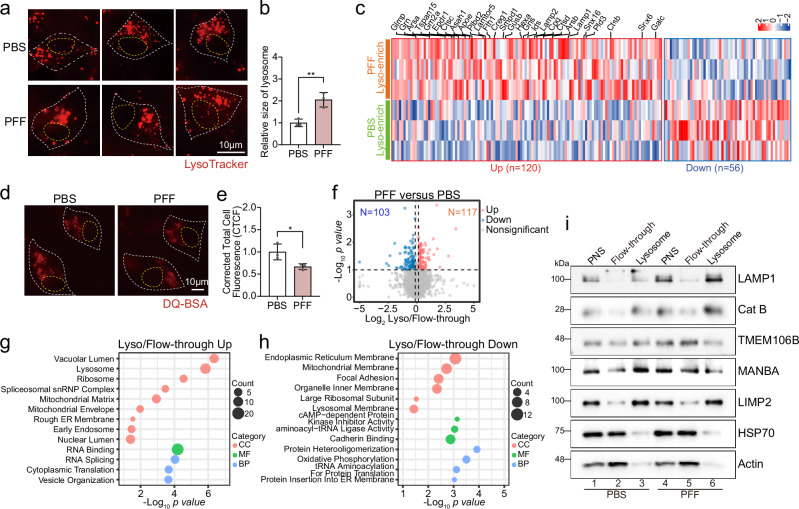
PFF disrupts lysosomal proteome and alters lysosomal function in cortical neurons. **a** Representative live-cell imaging of lysosome by LysoTracker Red DND-99 uptake in mouse primary cortical neurons treated with either preformed fibrils (PFFs) or PBS. Dashed lines outline cell boundaries. Scale bar, 10 µm. **b** Quantitative analysis of the relative size of lysosomes based on LysoTracker fluorescence. >30 cells were analyzed per condition. Data are presented as mean ± SD from *n* = 3 independent experiments. Statistical significance was determined by unpaired Student’s *t* test; **p < 0.01. **c** Heatmap shows the EnScore change of lysosome-enriched proteins in response to PFF versus PBS. Key lysosomal proteins, including enzymes and structural proteins are labeled. Red and blue indicate upregulation and downregulation, respectively. Analyses based on proteomic data from *n* = 3 independent experiments. **d** Representative live-cell imaging of DQ™ Red BSA fluorescence in primary cortical neurons treated with PFF or PBS to assess lysosomal proteolytic activity. Dashed lines outline cell boundaries. Scale bar, 10 µm. **e** Quantitative analysis of corrected total cell fluorescence (CTCF) from DQ-BSA imaging. >30 cells were analyzed per condition. Data are presented as mean ± SD (*n* = 3); unpaired Student’s *t* test; *p < 0.05. **f** Volcano plot showing the change in abundance of proteins between lysosomal and flow-through fractions in PFF-treated neurons versus PBS control. Lyso/Flow-through-Up proteins (orange) showed increased lysosomal localization versus flow-through, and Lyso/Flow-through-Down proteins (blue) were significantly reduced in lysosomal fractions. Nonsignificant proteins are shown in gray. Analyses based on proteomic data from *n* = 3 independent experiments. **g**, **h** Gene Ontology (GO) enrichment analysis of differentially fractionated proteins between PFF and PBS treatments. The left panel shows enriched GO terms for Lyso/Flow-through-Up proteins (**g**), while the right panel shows enriched terms for Lyso/Flow-through-Down proteins (**h**). Categories include cellular components (CC), molecular functions (MF), and biological processes (BP), with the size of the circles reflecting the number of proteins in each category and the color corresponding to the category type. Analyses based on proteomic data from *n* = 3 independent experiments. **i** Western blot analysis of protein distribution across post-nuclear supernatant (PNS), flow-through, and lysosome fractions. Representative blot from *n* = 3 independent experiments. Corresponding quantification of lysosome/flow-through ratios showed increased ratios for LAMP1 (+51.5%, *p* = 0.0493) and Cat B (+37.8%, *p* = 0.0137), and reduced ratios for TMEM106B (−43.1%, *p* = 0.0003) and MANBA (−35.3%, *p* = 0.0497) following PFF treatment; unpaired Student’s *t* test.

We initiated our studies by focusing on the impact of PFF on lysosomes. To investigate the effects of PFF, in mouse primary cortical neurons we treated with PFFs, or with α-synuclein monomers or PBS as controls (Supplementary Fig. 1a). Phosphorylation at Ser129 (pSer129) is a hallmark of α-synuclein aggregates and is widely used as a pathological marker^32^. Using a pSer129-α-synuclein-specific antibody, we confirmed α-synuclein aggregation in PFF-treated cultures; no signal was detected in PBS- or monomer-treated controls. Western blot analysis further demonstrated a dose-dependent increase in pSer129 levels following PFF treatment, whereas monomers and PBS controls had no effect (Supplementary Fig. 1b). In all following studies we used the lowest concentration (1 μg/mL) to explore PFF-mediated pathological phenotypes. To examine the impact on the ELN, we first assessed lysosomes, a compartment that continuously adjusts its number, size, and composition in response to internal and external stressors^33^. Live-cell imaging with LysoTracker Red DND-99 revealed a significant increase in lysosomal area in PFF-treated neurons (Fig. 1a); quantitation showed a 2-fold increase in puncta size in the PFF group as compared to PBS- or monomer-treated controls (Fig. 1b and Supplementary Fig. 1c), with no significant change in the number of lysosomal puncta across treatments (Supplementary Fig. 1d).

To assess whether lysosomal protein composition is altered following PFF-induced lysosome enlargement, we isolated lysosome from the post-nuclear supernatant (PNS) using dextran-coated magnetite beads. The flow-through fraction, containing the remaining contents of cytoplasm, was also collected. The total PNS, flow-through, and isolated lysosomal fractions were subjected to mass spectrometric (MS) analysis to compare proteomic profiles (Supplementary Fig. 1e). Principal component analysis (PCA) of proteomic data revealed a protein composition in lysosomes that was distinct from both the PNS and flow-through fractions, reflecting a collection of proteins characteristic of lysosomes (Supplementary Fig. 1f). To compare lysosome composition in PFF- versus control-treated cultures, we first calculated an enrichment score (EnScore) for each protein, defined as the ratio of its abundance in the lysosomal fraction relative to that in the PNS. We identified 528 lysosome-enriched proteins in PBS-treated neurons and 583 in PFF-treated neurons, results visualized in volcano plots (Supplementary Fig. 1g, h and Supplementary Data 1). Gene ontology (GO) analysis of enriched lysosomal proteins with respect to cellular compartment (CC) revealed that top terms were lysosome-related in both conditions (Supplementary Fig. 1i, j and Supplementary Data 1), confirming the specificity of our approach to lysosome isolation and protein identification. Significantly, over 40% of the proteins enriched in PFF-treated lysosomes did not overlap with those from PBS-treated lysosomes (Supplementary Fig. 1k), suggesting substantial changes in lysosomal protein content due to PFF treatment.

Further analysis of EnScore differences identified 120 proteins with increased enrichment (ΔEnScore > 0.4) and 56 proteins with decreased enrichment (ΔEnScore < −0.4) under the PFF versus the control condition (Fig. 1c and Supplementary Data 2). GO analysis of the 120 increased enriched proteins revealed significant overrepresentation of lysosome-associated cellular components, with “lysosome lumen” and “lysosome” ranking as the top terms (Supplementary Data 2). Among these were 61 lysosomal or late endosomal proteins; a subset of their gene names is highlighted in the heatmap (Fig. 1c). Key proteins with increased abundance in PFF-treated lysosomes included structural markers LAMP1 and LAMP2, and lysosomal enzymes such as Cathepsin B (Cat B), Cathepsin C (Cat C), and iduronate 2-sulfatase (IDS); these findings were confirmed by immunoblotting of isolated lysosomes (Supplementary Fig. 1l). Interestingly, components of high-density lipoprotein (HDL) and low-density lipoprotein (LDL) complexes, collagen-containing extracellular matrix, and Box C/D ribonucleoproteins complex were also enriched upon PFF treatment, suggesting accumulation of lysosomal substrates (Supplementary Data 2).

To ask if these changes were associated with alterations in lysosomal function, we assessed lysosomal degradative capacity using DQ-BSA, a self-quenched fluorescent substrate that emits fluorescence upon proteolytic degradation within lysosomes. Live-cell imaging revealed a marked reduction in DQ-BSA fluorescence in PFF-treated neurons (Fig. 1d), indicating impaired lysosomal degradation. Total cellular BSA uptake remained comparable across groups (Supplementary Fig. 1m), serving as a control for endocytic activity. Quantitative analysis confirmed a significant decrease in proteolytic activity in PFF-treated samples (Fig. 1e), supporting functional impairment despite elevated levels of some lysosomal enzymes. To further explore lysosomal function, we asked if PFF treatment altered the subcellular distribution of lysosomal proteins. Specifically, we compared the protein compositions of the lysosomal and cytoplasmic flow-through fractions to identify potential changes in protein localization. Using a fold-change cutoff of >1.1 for the Lyso/Flow-through ratio difference between treatments, volcano plots revealed shifts in protein localization, with 103 proteins showing reduced lysosomal localization (Lyso/Flow-through-Down) and 117 exhibiting increased localization (Lyso/Flow-through-Up) upon PFF treatment (Fig. 1f and Supplementary Data 3). GO analysis of Lyso/Flow-through-Up proteins enriched in cellular compartments such as the vacuolar lumen, lysosome, rough ER, with functional roles in vesicle organization, as well as mitochondrial envelope and mitochondrial matrix (Fig. 1g). Notably, PFF treatment led to increased abundance of ribosomal proteins as well as nuclear lumen proteins in the lysosomal fraction. The latter may reflect pathological perinuclear lysosome clustering, as reported in LRRK2 mutant models^34^. Overall, Lyso/Flow-through-Up proteins may reflect decreased degradation. Lyso/Flow-through-Down proteins included those in ER membrane, mitochondrial membrane, and those participating in oxidative phosphorylation and protein translation (Fig. 1h), raising the possibility of reduced delivery of substrates to the lysosome for degradation. Among Lyso/Flow-through-Up proteins, LAMP1 and Cathepsin B showed increased levels in both total and lysosomal fractions as confirmed by immunoblotting (Fig. 1i). Conversely, the lysosomal transmembrane protein TMEM106B and the hydrolase enzyme MANBA, seen in the Lyso/Flow-through-Down group, though little changed in the PNS, were significantly reduced in the lysosomal fraction. These changes point to redistribution of multiple lysosome-associated proteins into the flow-through compartment. LIMP2, a lysosomal membrane protein, and HSP70, a molecular chaperone involved in lysosomal function, which showed no change in MS analysis, were included as lysosomal controls in immunoblotting, and Actin was used as a general loading control. Taken together, the findings argue that PFF treatment disrupts both cargo delivery and alters the subcellular distribution of lysosome-associated proteins, leading to changes in protein localization consistent with impaired lysosomal function.

### PFF-induced lysosomal stress triggers activation of TFEB/TFE3 and chromatin remodeling

**Fig. 2:**
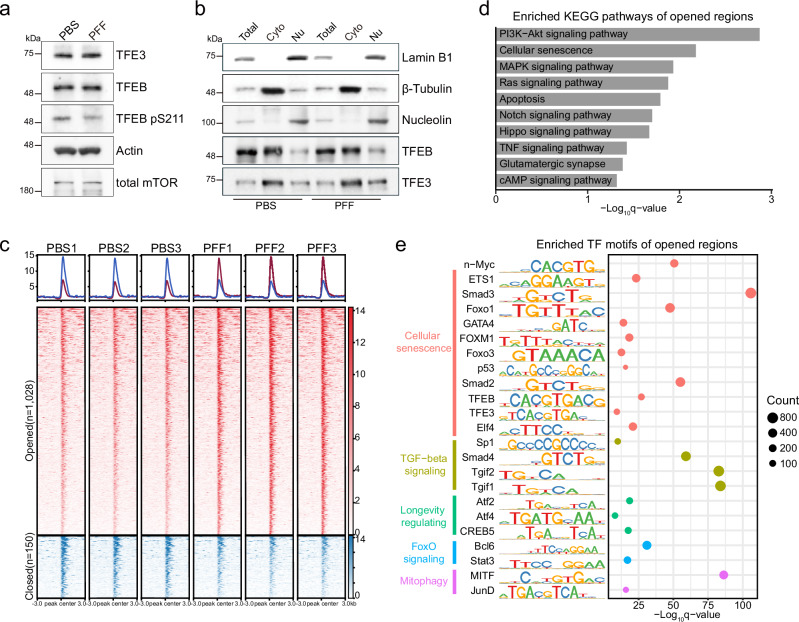
Chromatin accessibility changes and regulatory motif enrichment in PFF-treated neurons. **a** Western blot analysis of total protein levels of TFE3, TFEB, and phosphorylated TFEB (p-TFEB at Ser211). Total mTOR was also probed. Actin was used as a loading control. Representative image from *n* = 3 independent experiments. **b** Western blot analysis of TFE3 and TFEB localization in cellular fractions of neurons treated with PFF or PBS. Total (cell lysates), Cyto (cytoplasmic extract), Nu (nuclear extract). β-Tubulin served as a cytoplasmic marker, Lamin B1 as a nuclear marker, and nucleolin as a nucleolar marker. Representative image from *n* = 4 independent experiments. **c** ATAC-seq heatmaps showing regions with significantly increased (top, red) and decreased (bottom, blue) chromatin accessibility in mouse cortical neurons treated with PFF for 7 days, compared to PBS. Signal intensity is shown ±3 kb around the center of differential peaks. Differential peaks were identified using DiffChIPL with a cutoff of adjusted *p* < 0.05. Analyses based on ATAC-seq data from *n* = 3 independent experiments. **d** KEGG pathway enrichment analysis of genes linked to regions with significantly increased ATAC-seq peaks. Analyses based on ATAC-seq data from *n* = 3 independent experiments. **e** Enriched transcription factor (TF) motifs in regions with increased chromatin accessibility. The left panel shows pathways associated with each TF; the middle panel displays the name and motif sequence of enriched TFs; the right panel shows the enrichment significance (-Log_10_ *q*-value) and the number of predicted motif occurrences for each TF. Analyses based on ATAC-seq data from *n* = 3 independent experiments.

Lysosomal stress activates transcriptional programs that promote lysosome biogenesis and function, primarily through the transcription factors TFEB and TFE3, which translocate from the cytoplasm to the nucleus under stress conditions. Given their central role in lysosomal homeostasis and autophagy, we asked whether PFF-induced lysosomal dysfunction engages TFEB/TFE3 signaling. To this end, we examined the phosphorylation status and subcellular distribution of TFEB and TFE3 in primary cortical neurons following PFF exposure. Phosphorylation of TFEB serves as a marker of its cytoplasmic retention and inactivity. Despite the absence of significant changes in total TFEB/TFE3 levels in PFF-treated neurons, there was a ~30% reduction in phosphorylated TFEB (Fig. 2a and Supplementary Fig. 2a). As nuclear localization of TFEB/TFE3 is a prerequisite for function, we next examined whether PFF treatment alters their subcellular distribution. We first assessed TFEB localization by immunofluorescence. Under control conditions, TFEB was predominantly cytoplasmic, whereas PFF-treated neurons exhibited a marked increase in nuclear TFEB signal, consistent with activation-associated nuclear translocation (Supplementary Fig. 2b). To independently validate this observation and enable quantitative analysis, we performed biochemical fractionation of cytoplasmic and nuclear compartments. Using beta-tubulin and nucleolin as markers for cytoplasmic (Cyto) and nuclear (Nu) fractions, respectively, we confirmed effective fractionation. In PFF-treated neurons, we observed increased nuclear localization of TFEB/TFE3 compared to controls (Fig. 2b), as quantified by the ratio of nuclear to total protein abundance (Supplementary Fig. 2c). Specifically, nuclear TFEB increased by ~25% (1.25-fold), and TFE3 by ~41% (1.41-fold), upon PFF treatment. These findings indicate that PFF-induced lysosomal stress leads to TFEB/TFE3 dephosphorylation and nuclear translocation.

To investigate whether TFEB/TFE3 activation in PFF-treated neurons is associated with transcriptional remodeling, we performed genome-wide profiling of chromatin accessibility using ATAC-seq in neurons treated with PFF or PBS for 7 days. PFF treatment resulted in extensive chromatin remodeling, marked by 1028 regions with significantly increased (“up-peaks”) and 150 regions with significantly decreased (“down-peaks”) chromatin accessibility based on a cutoff of an adjusted *p*-value < 0.05 and a fold-change ≥1.5 (Fig. 2c and Supplementary Data 4). Genomic annotation revealed that down-peaks were primarily located in distal intergenic regions and introns, whereas up-peaks were more frequently found in promoter-proximal regions, with 17.6% of up-peaks mapping to promoters, compared to 7.1% of down-peaks (Supplementary Fig. 2d). We next annotated differentially accessible peaks to their nearest genes, identifying a total of 1073 associated genes. KEGG pathway enrichment analysis of genes linked to up-peaks revealed significantly enriched pathways related to neuronal function and aging, including PI3K-Akt signaling, MAPK signaling, cAMP signaling and apoptosis (Fig. 2d). The cellular senescence pathway was among the top enriched terms, suggesting involvement of senescence-associated processes following PFF treatment. Supporting this, we observed a reduction of total Lamin B1 levels (Fig. 2b), a structural nuclear lamina protein whose downregulation is a well-established marker of senescence-associated chromatin remodeling^35^. Genes associated with down-regulated chromatin regions corresponded to only four pathways, possibly reflecting the relatively small number of down-peaks (Supplementary Fig. 2e). Focusing on regions with increased chromatin accessibility, we performed motif analysis using the HOMER suite to identify potential transcription factors. There was prominent enrichment of TFEB and TFE3 motifs (Fig. 2e). Additionally, transcription factors associated with cellular senescence (e.g., ETS1, Foxo1, FOXM1, p53), TGF-beta signaling (e.g., Smad4, Tgif2), longevity regulation (e.g., Atf4, CREB5), and FoxO signaling (e.g., Bcl6, Stat3) were identified.

Given the role played by the lysosome in coordinating endocytic and autophagic vesicle fusion, we next examined the status of mechanistic target of rapamycin complex 1 (mTORC1), a kinase complex associated with the lysosomal membrane that integrates nutrient and stress signals to modulate cellular metabolism^36^. In PFF-treated neurons, there was no significant change in total mTOR levels. However, phosphorylation of mTOR was increased by approximately 36% in the lysosomal fraction (Supplementary Fig. 2f, g). To more directly assess mTORC1 signaling at lysosomes, we examined the canonical downstream substrate 4E-BP1 and found that lysosome-associated phosphorylation of 4E-BP1 was increased following PFF treatment (Supplementary Fig. 2h), indicating enhanced mTORC1 activity at the lysosomal membrane. In parallel, we examined the phosphorylation status and subcellular localization of TFEB. Despite increased lysosomal mTORC1 signaling, phosphorylation of TFEB at Ser211 within the lysosomal fraction was reduced (Supplementary Fig. 2i), together with reduced abundance of lysosome-associated TFEB. Correlated with this finding was the increase in aggregated α-synuclein in the lysosome fraction (Supplementary Fig. 2f). Enhanced mTOR activity in lysosomes harboring aggregated α-synuclein supports the view that PFF-mediated changes in lysosomes disrupts cellular metabolic function. Taken together, our findings demonstrate a correspondence between PFF-mediated changes in chromatin accessibility and diverse biological pathways. They reinforce a role for PFF acting through lysosomal stress to contribute to neuronal dysfunction.

### Transcriptomic analysis uncovers senescence-like transcriptional programs in neurons

**Fig. 3:**
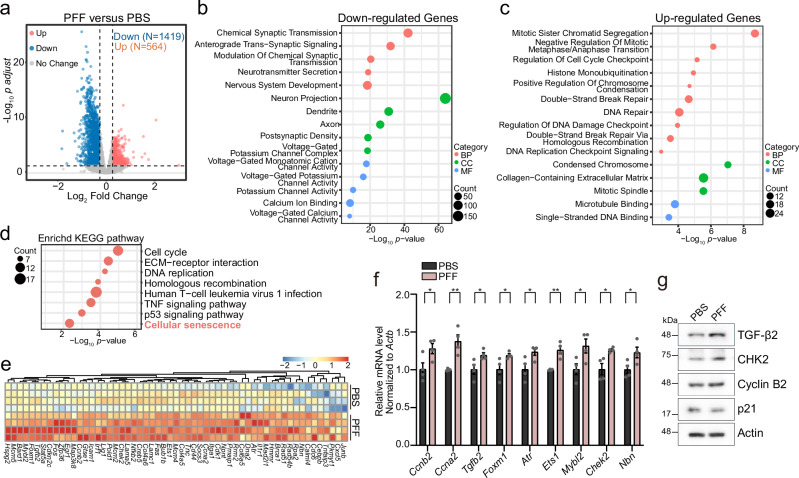
Transcriptomic profiling reveals PFF-induced senescence-associated transcriptional programs in cortical neurons. **a** Volcano plot showing differentially expressed genes in PFF-treated neurons compared to PBS. Upregulated genes are shown in orange, downregulated genes in blue, and unchanged genes in gray. Analyses based on RNA-seq data from *n* = 4 independent experiments. **b**, **c** Gene Ontology (GO) enrichment analysis of downregulated genes (**b**) and upregulated genes (**c**) in response to PFF treatment, with categories including biological processes (BP), cellular components (CC), and molecular functions (MF). Analyses based on RNA-seq data from *n* = 4 independent experiments. **d** KEGG pathway enrichment analysis of upregulated genes, revealing significant enrichment in top-ranked pathways related to cell cycle regulation, p53 signaling, and cellular senescence. Analyses based on RNA-seq data from *n* = 4 independent experiments. **e** Heatmap showing the expression patterns of genes related to the cellular senescence pathway, comparing PBS- and PFF-treated neurons. Analyses based on RNA-seq data from *n* = 4 independent experiments. **f** RT-qPCR analysis of differentially expressed genes (DEGs) related to cell senescence. The relative mRNA levels are normalized to *Actb*. Data are presented as mean ± SD from *n* = 4 independent experiments. Statistical significance was determined by unpaired Student’s *t* test; *p < 0.05, **p < 0.01. **g** Western blot analysis of protein levels associated with cell senescence genes, including TGF-β2, CHK2, Cyclin B2, and p21. Actin is used as a loading control. Representative image from *n* = 3 independent experiments.

To explore the downstream effects of PFF-induced chromatin remodeling, we next investigated transcriptional changes using RNA-seq on primary cortical neuron cultures. With a fold-change cutoff of 1.2 and an adjusted *p*-value < 0.05, we identified 564 upregulated genes and 1419 downregulated genes in PFF-treated neurons as compared to vehicle controls (Fig. 3a and Supplementary Data 5). Gene Ontology (GO) analysis of downregulated genes pointed to significant disruption of key neuronal functions, including chemical synaptic transmission, anterograde trans-synaptic signaling, and neurotransmitter secretion (Fig. 3b). Additionally, pathways related to voltage-gated ion channel activity and neuron projections, both dendritic and axonal, were impaired. GO analysis of upregulated genes indicated strong activation of cell cycle regulation and DNA repair pathways, functions that serve as key components of the cellular senescence program (Fig. 3c). These included mitotic sister chromatid segregation, cell cycle checkpoint regulation, and DNA damage responses such as double-strand break repair and replication checkpoint signaling, suggesting responses to secure genomic stability. Although the lysosomal pathway did not reach statistical significance in this enrichment analysis (*p* > 0.05), eight lysosome-associated genes *Arsg, Prkcd, Manba, Plekhf1, P2rx4, Cp, Aga,* and *Hspg2* were upregulated (Supplementary Data 5). Their upregulation was further validated by RT-qPCR analysis, with relative mRNA levels normalized to *Actb* (Supplementary Fig. 3a). Among these, *Arsg, Manba,* and *Aga* encode enzymes responsible for breakdown of substrates in lysosomes. PLEKHF1(also known as Phafin1), which contains a Pleckstrin homology (PH) and Fab1-YotB-Vac1p-EEA1 (FYVE) domain, is implicated in autophagy and lysosomal degradation pathways^37^. Changes in mRNA levels for these genes may reflect transcriptional activation to maintain cellular homeostasis. CLEAR (Coordinated Lysosomal Expression and Regulation) motif scanning analysis of the promoter regions of these lysosomal genes identified at least one high-confidence binding motif of TFEB in each gene (Supplementary Fig. 3b). Previous studies have shown that TFEB overexpression in Hela cells led to the induction of 291 genes, of which only 20 were lysosomal genes containing CLEAR sites, suggesting that TFEB regulates a wide range of cellular pathways beyond lysosomal function^38^. To further investigate the regulatory influence of TFEB, we analyzed the promoter regions of all 564 upregulated genes identified from our RNA-seq data and found that 307 of them contained at least one TFEB binding motif (Supplementary Data 5), highlighting the potentially broad regulatory impact of TFEB in response to PFF treatment.

KEGG pathway enrichment analysis of the upregulated genes further supported the activation of senescence-associated pathways (Fig. 3d). We noted, in particular, the increases in cell cycle, DNA replication, TNF signaling and p53 signaling. A heatmap of expression profiles for senescence-related genes in PFF-treated neurons, as compared to PBS controls, is shown (Fig. 3e). Among upregulated genes was *Ets1*, a transcription factor that regulates cell proliferation and senescence, and *Tgfb2*, a key component of the TGF-beta signaling pathway whose activities include promoting senescence. Other significant upregulated genes included *Nbn* (involved in DNA repair), *Chek2* (a crucial DNA damage checkpoint kinase), and *Il1r1*, which regulates senescence-associated inflammatory signaling, along with *Map3k8* and *Cxcl5*, which mediate the senescence-associated secretory phenotype (SASP) profile. These changes add to those revealed by chromatin remodeling to implicate activation of senescence-associated regulatory programs in PFF-treated neurons.

To validate these findings, we performed RT-qPCR on a subset of senescence-associated genes and confirmed consistent upregulation (Fig. 3f). Western blot analysis further corroborated these results, showing increased protein levels of TGF-β2 (*Tgfb2*), Cyclin B2 (*Ccnb2*), and CHK2 (*Chek2*) (Fig. 3g). However, p21 (*Cdkn1a*), a commonly used marker of cellular senescence, showed no significant change at the mRNA level by RNA-seq or at the protein level, as confirmed by Western blot. Additionally, p16 (*Cdkn2a*) mRNA was undetectable in our RNA-seq data. To assess additional features of cellular senescence, we examined senescence-associated β-galactosidase (SA-β-gal) activity and found no significant differences comparing PFF-treated and control neurons (Supplementary Fig. 3c). Similarly, measurements of mitochondrial membrane potential and ROS levels showed no noteworthy changes (Supplementary Fig. 3d, e). However, using the Glycolysis/OXPHOS assay kit, we detected a significant metabolic shift towards glycolysis (Supplementary Fig. 3f), a metabolic feature characteristic of senescence-associated cellular programs. We conclude that PFF treatment of cortical neurons induced senescence-like changes in gene expression and speculate that post-mitotic neurons may engage senescence-linked regulatory mechanisms distinct from those in dividing cells^39^.

### Integrative analysis of chromatin accessibility and gene expression in PFF-treated neurons

**Fig. 4:**
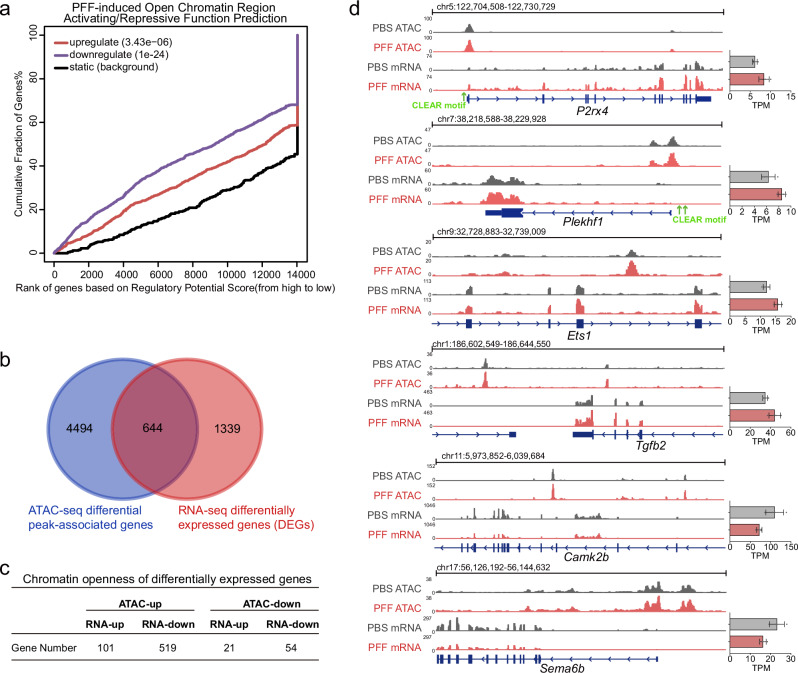
Integrative analysis of chromatin accessibility and gene expression changes in response to PFF treatment. **a** Correlation between PFF-induced chromatin accessibility and gene expression changes. The x-axis represents genes ranked by their Regulatory Potential Score, sorted from highest to lowest, with higher scores indicating stronger regulatory potential. The y-axis shows the cumulative fraction of genes, reflecting the proportion of genes within each category. Red and purple curves correspond to upregulated and downregulated genes, respectively, while the black curve represents static (background) genes with no significant expression changes. Statistical significance (*p*-values) was determined by comparing upregulated and downregulated genes to the static gene set. **b** Overlap between differentially expressed genes (DEGs) identified by RNA-seq and genes associated with differentially accessible chromatin regions identified by ATAC-seq. **c** Table showing the number of overlapped genes exhibiting different patterns of chromatin accessibility and gene expression. **d** Peak diagram showing the transcriptional abundance of differentially expressed genes and the corresponding ATAC-seq peaks in the adjacent region. The top two tracks display ATAC-seq abundance under PBS and PFF conditions, while the bottom two tracks show RNA-seq abundance. The blue lines at the bottom represent the gene structure, and the genomic coordinates of the visualized region are shown at the top. The green arrow highlights the location of the CLEAR motif. The bar chart on the right represents the TPM (transcripts per million) values for the two conditions, with PBS shown in gray and PFF in red.

To further investigate the regulatory mechanisms underlying the transcriptomic changes observed in PFF-treated neurons, we integrated ATAC-seq and RNA-seq data to examine how PFF-induced chromatin remodeling impacted gene expression. First, we analyzed the relationship between chromatin accessibility and gene expression to ask if the changes in accessibility were associated with transcriptional activation or repression. Increased chromatin accessibility was strongly correlated with upregulated gene expression suggesting that PFF-induced chromatin opening facilitates transcriptional activation (Fig. 4a). Interestingly, an even stronger correlation was observed between increased chromatin accessibility and downregulated gene expression, indicating that increased accessibility may participate in both transcriptional activation and repression. In contrast, regions with reduced chromatin accessibility did not show a clear correlation with gene expression changes (Supplementary Fig. 4a), suggesting that the decreased accessibility may be functionally silent. Genomic feature analysis of “up peaks” showed that 36.6% were near gene promoter regions (Supplementary Fig. 4b), consistent with a direct role in gene regulation. Analysis of “down peaks” revealed that most were located in the intron and distal intergenic regions far from promoter sites (Supplementary Fig. 4b).

To increase sensitivity of this analysis, we applied a less stringent cutoff for ATAC-seq (adjusted *p*-value < 0.05 and fold-change ≥1.3) and identified 7660 differentially accessible peaks corresponding to 5138 associated genes. We examined the links between genes with altered chromatin accessibility and differentially expressed genes (DEGs) derived from RNA-seq analysis. Of the 5138 differentially accessible peak-associated genes, 644 were shown to overlap with DEGs (Fig. 4b and Supplementary Data 6). Among the overlaps, 519 of those with up-peaks in their promoter regions were significantly downregulated, while 101 were upregulated at the transcript level (Fig. 4c and Supplementary Fig. 4c). The predominance of downregulated DEGs with increased chromatin accessibility supports the observed stronger correlation between “up peaks” and gene repression. Additionally, peak annotation revealed that “up peaks” linked to gene activation were more frequently located in promoter-proximal regions (≤1 kb) (Supplementary Fig. 4d), consistent with the established positive correlation between promoter accessibility and transcriptional activity. Conversely, “up peaks” associated with repressed genes were somewhat more enriched in regions distal to promoters, such as the first intron and distal intergenic regions, highlighting a role for spatially distributed regulatory elements involved in gene repression. Among the genes associated with “down peaks”, only 21 were significantly upregulated and 54 downregulated (Fig. 4c), reflecting the weaker correlations for “down peaks” and gene expression.

To further illustrate these relationships, we highlighted representative genes showing concordant or discordant changes in accessibility and expression (Fig. 4d). For instance, *P2rx4*, which encodes functional ATP-activated cation channels on lysosomal membranes^40^, exhibited increased mRNA levels in PFF-treated neurons together with increased promoter accessibility containing a predicted CLEAR motif. Another lysosomal gene, *Plekhf1*, displayed a comparable pattern of regulation. Notably, senescence-associated genes *Ets1* and *Tgfb2* also displayed both increased chromatin accessibility and elevated transcript levels. In contrast, *Camk2b* and *Sema6b*, two genes linked to neuronal signaling and plasticity, had significant chromatin opening but lower transcriptional output, illustrating the complexity of gene regulation in response to PFF treatment.

### LRRK2 phosphorylates Rab5 on early endosomes

**Fig. 5:**
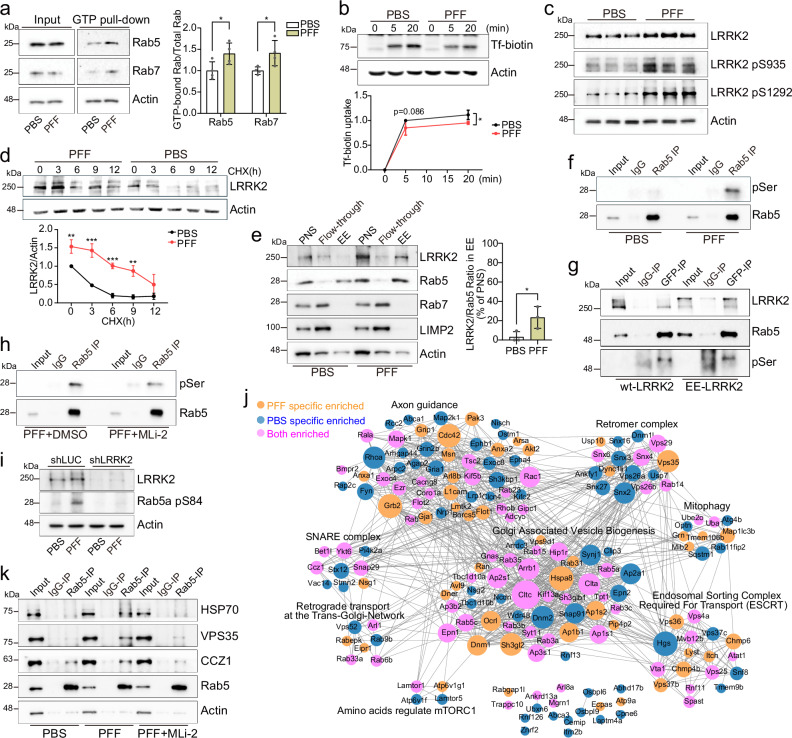
LRRK2-mediated Rab5 phosphorylation remodels the Rab5 interactome in neurons. **a** GTP-bound Rab5 and Rab7 levels in PBS- and PFF-treated cortical neurons were measured using GTP-agarose pull-down assays. Western blot analysis of Rab5 and Rab7 level in total cell lysates (Input) and GTP pull-down fractions. Quantification of the GTP-bound Rab relative to total Rab protein level following PBS or PFF treatment (right panel). Data are presented as mean ± SD from *n* = 4 independent experiments. Statistical significance was determined by unpaired Student’s *t* test; *p < 0.05. **b** Endocytic activity of mouse cortical neurons treated with PFF or PBS were evaluated by incubation with 10 µg/ml biotinylated transferrin (Tf-biotin) for the indicated times. Internalized Tf-biotin was detected using HRP-conjugated streptavidin. Quantification of Tf-biotin/Actin ratios at each time point was based on *n* = 4 independent experiments. Data are presented as mean ± SD; unpaired Student’s *t* test; *p < 0.05. **c** Western blot analysis of total LRRK2 and phosphorylated LRRK2 at serine935 (pS935) and serine1292 (pS1292) in PBS- and PFF-treated cortical neurons. Actin served as the loading control. Representative image from *n* = 3 independent experiments. **d** LRRK2 protein half-life was assessed in PBS- and PFF-treated cortical neurons following treatment with 100 μg/ml cycloheximide (CHX) for the indicated durations. Data are presented as mean ± SD (*n* = 3); unpaired Student’s *t* test; **p < 0.01, ***p < 0.001. **e** Western blot analysis of LRRK2, Rab5, Rab7 and LIMP2 in post-nuclear supernatant (PNS), flow-through, and early endosome (EE) fractions from PBS- and PFF-treated primary neurons. Rab5, Rab7, and LIMP2 were included as markers for early endosomes, late endosomes, and lysosomes, respectively. Actin served as a loading control. Early endosomes were isolated using dextran-coated magnetite beads. In PBS-treated samples, ~65% of PNS LRRK2 was detected in the flow-through fraction and ~4% in the EE fraction. Following PFF treatment, ~47% of PNS LRRK2 was detected in the flow-through fraction and ~18% in the EE fraction. Quantification of the LRRK2-to-Rab5 protein ratio in EE relative to PNS following PFF treatment is shown (right panel). Data are presented as mean ± SD (*n* = 3); unpaired Student’s *t* test; *p < 0.05. **f** Primary neurons treated with PBS or PFF were immunoprecipitated using a Rab5-specific antibody or IgG. The levels of phosphoserine and Rab5 in the immunoprecipitants were analyzed by Western blot. Representative image from *n* = 3 independent experiments. **g** Mouse neuroblastoma cell line (N2a) transfected with GFP-Rab5 was co-transfected with either myc-tagged wild-type LRRK2 or EE-targeted LRRK2. Cell lysates were immunoprecipitated using a GFP antibody or IgG. The levels of phosphoserine, Rab5, and LRRK2 were assessed by Western blot. Representative image from *n* = 3 independent experiments. **h** Primary neurons treated with PFF in the presence of either MLi-2 or DMSO were subjected to Rab5 immunoprecipitation. Phosphoserine and Rab5 levels were analyzed by Western blot. Representative image from *n* = 3 independent experiments. **i** Primary cortical neurons were infected with lentivirus expressing control shRNA (shLUC) or LRRK2 shRNA (shLRRK2) and treated with PBS or PFF. Western blot analysis of total LRRK2, phosphorylated Rab5a at Ser84 (Rab5a pS84), with Actin as a loading control. Representative image from *n* = 3 independent experiments. **j** Protein-protein interaction (PPI) network of endosome proteins identified by Rab5 co-immunoprecipitation (co-IP). Proteins common to both PBS and PFF conditions are shown in purple; those uniquely enriched under PFF are in orange, and those specific to PBS in blue. Analyses based on proteomic data from *n* = 4 independent experiments. **k** Western blot validation of Rab5-associated proteins identified from proteomic analysis, examined under PBS, PFF, and PFF plus LRRK2 inhibitor MLi-2 conditions. Rab5 co-immunoprecipitation followed by immunoblotting for HSP70, VPS35, CCZ1, and Rab5. Actin served as a loading control. Representative image from *n* = 4 independent experiments.

Given our findings that PFF treatment alters lysosomal composition and impairs neuronal gene expression programs, we next sought to identify upstream molecular events that may drive these changes. In particular, we asked whether dysfunction within compartments upstream of lysosomes contributes to the observed disruption. We focused on Rab5, a master regulator of early endosome dynamics, and Rab7, which regulates late endosomes trafficking downstream of Rab5. These GTPases are activated in their GTP-bound state, allowing them to recruit downstream effectors and coordinate membrane trafficking^26^. Using a GTP-agarose pull-down assay, we measured the active forms of Rab5 and Rab7 in cortical neurons treated with PFF or PBS (Fig. 5a). PFF treatment significantly increased the levels of both GTP-loaded Rab5 and Rab7 with both showing ~1.4-fold elevation compared to PBS-treated controls, findings suggesting enhanced activation of these Rabs. To evaluate Rab5 function, we examined endocytosis, which is positively regulated by Rab5 activity, by measuring the internalization of biotinylated transferrin (Tf-biotin) at 0, 5, and 20 min after incubation. Despite the increased levels of GTP-bound Rab5, endocytosis was significantly impaired in PFF-treated neurons (Fig. 5b), suggesting that PFF acted to disrupt Rab5 function.

To explore how Rab5 function could be impacted by PFF treatment, we focused on a possible role for LRRK2, a kinase known to phosphorylate and modulate several Rab GTPases. Whether or not LRRK2 regulates Rab5 is equivocal. We found that although *LRRK2* mRNA levels were unresponsive to PFF treatment (Supplementary Fig. 5a), both total LRRK2 and phosphorylated LRRK2 (pS935) levels were markedly elevated in PFF-treated neurons (Fig. 5c). In parallel, phosphorylation of LRRK2 at Ser1292, an established marker of kinase activity, was also increased following PFF treatment (Fig. 5c). Notably, the pS1292/total LRRK2 ratio was significantly increased (Supplementary Fig. 5b), indicating that this increase was not simply due to greater LRRK2 abundance but reflected enhanced kinase activation. The discrepancy between mRNA and protein levels suggested that LRRK2 levels were regulated post-transcriptionally. To ask if reduced degradation contributes, we performed cycloheximide (CHX) chase assays and found that PFF treatment significantly prolonged LRRK2 half-life by approximately threefold (Fig. 5d). We hypothesized that membrane association might underline the observed increase in LRRK2 stability and kinase activation. Indeed, prior studies have shown that LRRK2 membrane association enhances its kinase activity and promotes dimerization, particularly at endolysosomal compartments, where it engages Rab substrates^41^. Given the PFF-mediated reduction in Rab5 function, we next asked if PFF treatment increased LRRK2 recruitment to early endosomes by isolating early endosomes from PFF- and PBS-treated neurons and assessing LRRK2 in the post-nuclear supernatant (PNS), cytoplasmic flow-through, and early endosome (EE) fractions. Remarkably, LRRK2 was present on early endosomes isolated from PFF-treated cultures (Fig. 5e); in contrast, LRRK2 levels were very low on early endosomes isolated from PBS-treated neurons. Quantitative analysis of the LRRK2/Rab5 ratio revealed more than a 7-fold increase, from 3.2% to 23.3%, in early endosomes after PFF exposure (Fig. 5e).

We next asked whether Rab5 was phosphorylated in PFF-treated neurons and, if so, whether LRRK2 was responsible. To detect phosphorylated Rab5, we used a GFP-tagged Rab5 expression system and performed immunoprecipitation (IP) of GFP from transfected neuroblastoma (N2a) cells, followed by phosphoserine (pSer) detection, the amino acid predicted to be phosphorylated by LRRK2^30^. In the GFP-IP fraction, but not in the IgG-IP control, a phosphoserine signal was detected at the expected molecular weight for Rab5 (Supplementary Fig. 5c). Note that the band for pSer is well separated from the mouse IgG heavy chain (Supplementary Fig. 5d). Next, in the GFP-Rab5 expressing N2a cell line we overexpressed myc-tagged wild-type LRRK2 of human origin (WT-LRRK2), or an empty vector, and repeated the immunoprecipitation experiments. In the presence of LRRK2 overexpression there was a significant increase in Rab5 phosphorylation (Supplementary Fig. 5e). The interaction between LRRK2 and Rab5 was supported by the detection of LRRK2 in the GFP-Rab5 IPs (Supplementary Fig. 5e). We then performed Rab5 immunoprecipitation in primary cortical neurons and confirmed that PFF treatment markedly increased phosphorylation of endogenous Rab5 (Fig. 5f). To test whether endosomal localization of LRRK2 facilitates Rab5 phosphorylation, we transfected N2a cells with GFP-Rab5 and either WT-LRRK2 or an early endosome-targeted LRRK2 construct (EE-LRRK2) for subsequent GFP immunoprecipitation (Supplementary Fig. 5f). Compared to cells expressing wild-type LRRK2, EE-LRRK2 exhibited stronger association with Rab5 in the GFP-Rab5 IP fraction and markedly enhanced Rab5 phosphorylation, as indicated by a 2.7-fold increase in pSer-Rab5/total Rab5 ratio (Fig. 5g and Supplementary Fig. 5g). Co-immunoprecipitation (co-IP) with a LRRK2 antibody after EE-LRRK2 overexpression further confirmed the interaction with endogenous Rab5a and Rab5b (Supplementary Fig. 5h); interestingly, relative to their total protein levels, enrichment of Rab5b greatly exceeded that for Rab5a. To ask if LRRK2 activity is required for Rab5 phosphorylation, we employed the selective LRRK2 inhibitor MLi-2. We first confirmed its efficacy in cultured neurons. As expected, MLi-2 treatment markedly reduced phosphorylation of LRRK2 at Ser935 and at the bona fide autophosphorylation site Ser1292, as well as phosphorylation of its canonical substrate Rab10 at Thr73, without altering total LRRK2 or Rab10 protein levels (Supplementary Fig. 5i), confirming that the inhibitor effectively blocks LRRK2 kinase activity. We then examined Rab5 phosphorylation in PFF-treated neurons and found that MLi-2 significantly reduced phosphoserine levels on immunoprecipitated Rab5 (Fig. 5h). Taken together, the findings are evidence that PFF treatment resulted in activation and recruitment of LRRK2 to early endosomes where it interacts with and phosphorylates Rab5.

We tentatively identified Ser84 as the Rab5 residue phosphorylated by LRRK2. To confirm this assignment, we employed an antibody specific to serine phosphorylated (pSer84) Rab5a. Antibody specificity was independently validated using Rab5 ASO-mediated depletion of endogenous Rab5. In primary neurons treated with PFF, Rab5 immunoprecipitation revealed a stronger pSer84 signal, coinciding with the presence of LRRK2 in complex with Rab5 and supporting their presence on Rab5-containing endosomal membranes (Supplementary Fig. 5j). Furthermore, Western blot analysis of total cell lysates showed that the PFF-induced increase in pSer84 was eliminated by MLi-2 treatment, confirming that Rab5a phosphorylation at Ser84 is mediated by LRRK2 activity (Supplementary Fig. 5k). To genetically corroborate these pharmacological findings, we knocked down LRRK2 in primary neurons using a lentiviral shRNA. LRRK2 knockdown largely abolished PFF-induced Rab5 Ser84 phosphorylation, reducing the signal by ~68% in shLRRK2 neurons relative to shLUC controls (Fig. 5i).

Finally, as additional stringent control to confirm that the PFF-induced increase in LRRK2 activation and Rab5 Ser84 phosphorylation reflects a neuron-intrinsic response, we repeated these analyses in cortical neuron cultures maintained under Ara-C conditions to suppress proliferating non-neuronal cells. Under these neuron-enriched conditions, PFF treatment produced the same increases in LRRK2 activation and Rab5 Ser84 phosphorylation (Supplementary Fig. 5l), further supporting that the LRRK2-Rab5 phosphorylation axis is engaged in neurons upon PFF exposure.

To directly assess whether LRRK2 also regulates Rab5 phosphorylation under basal conditions, we inhibited LRRK2 using MLi-2 or reduced its expression by shRNA-mediated knockdown in the absence of PFF treatment. Because basal pSer84-Rab5 levels are low, Rab5 was immunoprecipitated prior to pSer84 immunoblotting to enrich the signal. Under these conditions, both pharmacological inhibition and genetic reduction of LRRK2 resulted in a modest but reproducible decrease in basal Rab5 Ser84 phosphorylation (Supplementary Fig. 5m, n) indicating that LRRK2 contributes to low-level Rab5 Ser84 phosphorylation under basal conditions, while this regulatory axis is strongly amplified under PFF-induced pathological stress.

### LRRK2 mediates changes in the Rab5 interactome

Phosphorylation of Rab proteins can markedly impact their engagement of regulatory and effector proteins^42^. Given that Rab5 is phosphorylated by LRRK2 following PFF treatment, we next asked whether this modification alters the Rab5 interactome. To address this, we performed Rab5 co-IP using mild lysis buffer under both PBS and PFF-treated conditions. The IP and IgG fractions were analyzed by mass spectrometry, and enriched proteins were identified based on the IP/IgG ratio. We detected 876 Rab5-associated proteins in the PBS treatment condition and 819 in PFF-treated neurons (Supplementary Fig. 5o, p and Supplementary Data 7). Rab5 itself was among the most enriched proteins, confirming the efficiency of Rab5 co-IP. Since our method pulled down proteins from total cell lysates, it captured both active (GTP-loaded) and inactive (GDP-bound) forms of Rab5 along with their associated proteins. As Rab5 is primarily in its active GTP-bound state when associated with early endosome membranes^29^, to identify proteins interacting with the GTP-bound form, we cross-referenced our results with Gene Ontology annotations for endosome-related proteins. This database identified 1,213 endosomal proteins, of which 132 were present in the Rab5 immunoprecipitates of the PBS condition and 111 were found in the PFF treatment condition (Supplementary Fig. 5q, r). Comparing these Rab5-associated endosomal proteins sets we found that only one-third (64 out of 179) of the proteins were shared (Supplementary Fig. 5s). Notably, 68 were specific to PBS treatment and 47 were specific to PFF treatment (Supplementary Fig. 5s and Supplementary Data 7), documenting PFF treatment-induced changes in the Rab5 interactome. Protein-protein interaction (PPI) network analysis to compare the interacting proteins in the PBS and PFF treatment conditions revealed diverse functional categories (Fig. 5j), with enriched biological processes including Golgi-associated vesicle biogenesis, the trans-Golgi network, the retromer complex, axonal guidance, the SNARE complex, and ESCRT-mediated membrane trafficking. Proteins are color-coded by their condition-specific overlap class to highlight differential interaction patterns. These findings demonstrated that PFF exposure induced a broad remodeling of Rab5-associated endosomal machinery, with potential consequences for many vesicular transport and neuronal signaling pathways.

In addition to canonical endosomal proteins, Rab5 immunoprecipitates from PFF-treated neurons exhibited altered interactions with a broader set of regulators involved in inter-organelle communication, vesicle trafficking, and proteostasis (Supplementary Fig. 5t and Supplementary Data 7). Specifically, Rab5 association was reduced with VPS13D, a ubiquitin-binding regulator of mitochondrial dynamics and autophagy^43^, and with synaptotagmin-4 (SYT4), a protein involved in synaptic vesicle exocytosis^44^. In contrast, Rab5 binding was enhanced for several endolysosomal-autophagy (ELA)-related proteins, including GBA and ATP6V0A1, which contribute to lysosomal hydrolase activity and acidification, respectively^45,46^. We also observed increased Rab5 association with TDP-43 and UBQLN2, two stress-responsive proteins that regulate proteostasis through proteasomal degradation and autophagy^47^. These findings extend our proteomic analysis by showing that Rab5 phosphorylation not only perturbs core endosomal trafficking networks but also engages a subset of ELA components, with potential consequences for cargo recognition and endosomal sorting under pathological conditions.

To explore the mechanism by which PFF impacts the Rab5 interactome, we examined whether changes in endosomal proteins were dependent on LRRK2 activity. We performed Rab5 co-immunoprecipitation in neurons treated with PBS, PFF, or PFF combined with MLi-2, and analyzed a subset of proteins identified in our proteomic screen. These included CCZ1, a component of the Mon1-Ccz1 complex and a known GEF for Rab7, VPS35, a regulator of endosomal retrograde transport^48^, and HSP70, which facilities endosomal microautophagy and lysosomal membrane maintenance^49,50^. Compared to control, PFF treatment increased the levels of these proteins in the Rab5 IP (Fig. 5k and Supplementary Fig. 5u), with increases varying from 1.36-fold for CCZ1 to 2.4-fold for VPS35 and 2.1-fold for HSP70. The increases were significantly reduced or eliminated by inhibiting LRRK2 (Fig. 5k and Supplementary Fig. 5u), confirming their mediated by increased LRRK2 activity.

To further probe the functional consequences of Rab5 interactome remodeling, we examined additional endosomal markers. Both Rab7, a late endosome Rab immediately downstream of Rab5, and Rab11, which regulates endosomal recycling downstream of Rab5^51^, were significantly increased by ~1.5-fold in the Rab5 IP fraction from PFF-treated neurons; their enrichment was eliminated by LRRK2 inhibition (Supplementary Fig. 5v). To image changes in endosomal membranes, we employed an antibody against EEA1, a widely accepted and commonly used marker for early endosomes^52^. In agreement with the Rab5 co-IP results, immunostaining revealed markedly increased Rab7 colocalization with EEA1 in PFF-treated neurons (Supplementary Fig. 5w). Our findings support a model in which PFF activates LRRK2, leading to Rab5 phosphorylation with subsequent remodeling of the Rab5 interactome that drives downstream dysregulation of the ELN.

### Rapid LRRK2 recruitment to early endosomes triggered ELN changes

**Fig. 6:**
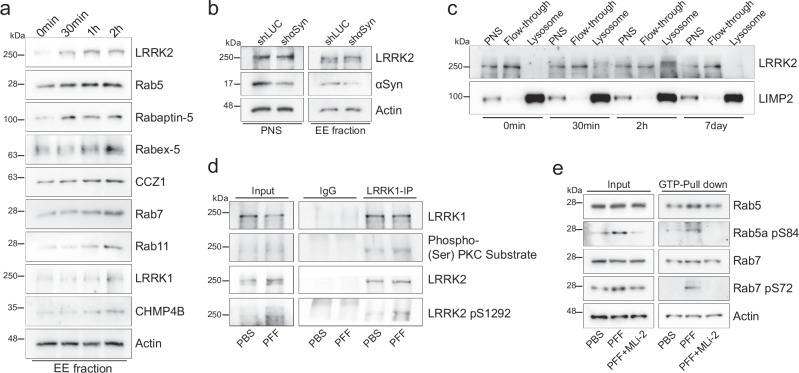
PFF triggers rapid recruitment of LRRK2 to endolysosomal compartments. **a** Early endosomes were purified from primary neurons treated with PFF for durations (0 min, 30 min, 1 h, and 2 h) using dextran-coated magnetite beads. The levels of LRRK2, Rab5, Rabaptin-5, Rabex-5, CCZ1, Rab7, Rab11, LRRK1 and CHMP4B in the early endosome fraction were assessed by Western blot. Actin served as a loading control. Representative image from *n* = 4 independent experiments. **b** Primary cortical neurons were infected with lentiviruses expressing either shRNA targeting mouse α-synuclein (shαSyn) or a control shRNA targeting luciferase (shLUC) for 3 days. Neurons were then treated with PFF for 30 min, followed by purification of early endosomes (EE). Post-nuclear supernatant (PNS) and EE fractions were analyzed by Western blot for LRRK2, αSyn and Actin. Representative image from *n* = 3 independent experiments. **c** Lysosomes were purified from primary neurons treated with PFF for (0 min, 30 min, 2 h, and 7 days) using dextran-coated magnetite beads. Western blot analysis of the distribution of LRRK2 in post-nuclear supernatant (PNS), flow-through, and lysosome fraction. LIMP2 served as a marker for lysosome. Representative image from *n* = 3 independent experiments. **d** LRRK1 co-immunoprecipitation (co-IP) was performed on lysates from primary neurons treated with PBS or PFF. The immunoprecipitates were analyzed by Western blot for LRRK1, pan-PKC phospho (Ser)-substrate, total LRRK2, and LRRK2 phosphorylated at serine1292 (LRRK2 pS1292). Representative image from *n* = 3 independent experiments. **e** Primary neurons were treated with PBS, PFF, or PFF in combination with the LRRK2 inhibitor MLi-2, followed by GTP-Rab pull-down assay. Western blotting was performed to assess total Rab5, Rab5a phosphorylated at Ser84 (Rab5a pS84), total Rab7, and Rab7 phosphorylated at Ser72 (Rab7 pS72). Representative image from *n* = 3 independent experiments.

Evidence for PFF-mediated changes in early endosomes raised the possibility that perturbations in this compartment may precede changes in the downstream ELN elements. To determine when PFF effects are first registered in early endosomes, we carried out time course studies in which PFFs were added to cortical neuron cultures for 30, 60 or 120 minutes prior to isolating early endosomes or lysosomes. Marked changes were observed at each timepoint. Notably, LRRK2 was recruited to early endosomes as early as 30 min post-treatment, indicating a rapid PFF-induced impact on this endosomal compartment (Fig. 6a). Rab5 was also increased at 30 min, as Rabaptin-5, a Rab5 effector. These increases, as well as an increase in Rabex-5, a GEF for Rab5, were maintained and further enhanced over the 2-h period. At 2 h, the increases were highly significant (6.2-fold for LRRK2, 1.7-fold for Rab5, 3.0-fold for Rabex-5, and 3.3-fold for Rabaptin-5). The rapid appearance of both LRRK2 and Rab5 further supports their presence together on early endosomes. There were also rapid and sustained increases Rab7 and its GEF CCZ1, suggesting that enhanced membrane association of this GEF contributes to elevated Rab7 levels (2.8-fold for Rab7 and 1.8-fold for CCZ1). Rab11 was similarly increased (6.2-fold at 2 h), accompanied by CHMP4B (3.0-fold at 2 h), a component of the ESCRT-III complex that appeared selectively in Rab5 co-IP following PFF treatment. Immunofluorescence analyses at the 2-h time point further revealed increased colocalization of LRRK2 with the early endosome marker EEA1, consistent with recruitment of LRRK2 to early endosomes (Supplementary Fig. 6a). Together with the evidence that the inhibition of LRRK2 reduced or eliminated the changes in Rab7, Rab11, CCZ1, and VPS35 (Fig. 5k and Supplementary Fig. 5u, v), we conclude that early endosomes represent an early site for LRRK2-mediated changes and argue that changes in the early endosome are propagated to downstream ELN elements.

Whether or not endogenous α-synuclein serves in the response of early endosome to PFF treatment was addressed by asking if reducing the level of endogenous α-synuclein impacts recruitment of LRRK2. We used lentiviruses encoding short hairpin RNAs against mouse *Snca* (shαSyn-1 and shαSyn-2) to reduce endogenous protein level. Both constructs reduced α-synuclein expression compared to the control, with shαSyn-2 achieving over 50% reduction (Supplementary Fig. 6b). Using shαSyn-2, we examined cortical neurons treated with PFF for 30 min followed by early endosome isolation. Cultures infected with shαSyn-2 lentivirus, but not control virus, showed a reduction in α-synuclein levels. Notably, despite reduced α-synuclein levels, there was no change in LRRK2 in the early endosome fraction of PFF-treated cells (Fig. 6b and Supplementary Fig. 6c). The finding is evidence that PFF-mediated recruitment of LRRK2 to early endosomes does not require endogenous α-synuclein. Rather, the evidence points to PFFs within early endosomes as responsible for rapid LRRK2 recruitment.

To assess how PFF exposure affects lysosomal recruitment of LRRK2 over time, we pre-labeled lysosomes by overnight incubation with magnetic beads and then added PFFs for the times listed above as well as for 7 days, followed by lysosome isolation. Remarkably, LRRK2 was detected on lysosomal fractions as early as 30 min post-treatment, with a further increase at 120 min (5.7-fold increase) (Fig. 6c and Supplementary Fig. 6d). To further examine this, immunofluorescence analyses at 120 minutes using a validated LAMP1 antibody (confirmed by overlap with LAMP2; Supplementary Fig. 6e), was performed in parallel with shLRRK2 to validate the signal generated by the LRRK2 antibody. The findings revealed partial colocalization of LRRK2 with the lysosomal marker LAMP1 in PFF-treated neurons (Supplementary Fig. 6f). Interestingly, LRRK2 recruitment to lysosomes was not sustained for 7 days, suggesting that LRRK2 recruitment to both early endosomes and lysosomes is an early response to PFF treatment. Given that 30 min is not likely to suffice for PFF to traffic through the endocytic pathway and reach lysosomes, the early lysosomal localization of LRRK2 was unexpected. To test whether LRRK2 recruitment was directly triggered by α-synuclein aggregates within lysosomes, we examined the distribution of higher-molecular-weight α-synuclein species in endolysosomal compartments at 30 min. Immunoblotting using an antibody that detects both mouse and human α-synuclein revealed aggregated species (≥17 kDa) in early endosomes, but not in lysosomes (Supplementary Fig. 6g, h), despite equal amounts of total aggregates in the post-nuclear supernatant of both conditions. These data suggest that LRRK2 recruitment to lysosomes does not depend on the presence within lysosomes of aggregated α-synuclein, raising the possibility that upstream signaling may serve to trigger lysosomal recruitment.

### PFF-induced LRRK2 activation promotes Rab7 phosphorylation

PFF treatment unexpectedly increased Rab7 phosphorylation at Ser72 (Supplementary Fig. 6i), a site previously attributed to LRRK1 activation^53^; the increase occurred in the absence of any detectable change in total LRRK1 protein levels. Although Rab7 is not a canonical substrate of LRRK2, its phosphorylation was suppressed by MLi-2 co-treatment (Supplementary Fig. 6j), arguing that LRRK2 activation in some way contributes. To clarify the respective roles of LRRK1 and LRRK2 in Rab7 phosphorylation, we performed shRNA-mediated knockdown of LRRK1 in primary neurons, with or without LRRK2 inhibition, and assessed Rab7 Ser72 phosphorylation (Supplementary Fig. 6k). LRRK1 knockdown markedly attenuated PFF-induced Rab7 Ser72 phosphorylation, resulting in an 82% reduction relative to shLUC controls, whereas LRRK2 inhibition alone produced a more modest reduction of 36.6%. A low residual pRab7 signal remained following LRRK1 knockdown under PFF treatment, likely reflecting incomplete suppression, and was further eliminated by LRRK2 inhibition. Basal Rab7 Ser72 phosphorylation under PBS conditions was also largely abolished by LRRK2 inhibition. Together, these data implicate LRRK1 as the principal kinase responsible for Rab7 phosphorylation in this context.

We next asked whether PFF-induced activation of LRRK1 depend on LRRK2. PFF treatment resulted in robust activation of LRRK1 accompanied by increased Rab7 phosphorylation (Supplementary Fig. 6l). Increased LRRK1 activation was inferred from enhanced detection of phospho-(Ser) PKC substrate signals in LRRK1 immunoprecipitates, using a pan-phosphoserine antibody that recognizes PKC targets^54^. Upon LRRK2 knockdown, these signals were partially reduced but remained elevated relative to PBS controls, with decreases of ~50% for LRRK1 activity and ~40% for Rab7 Ser72 phosphorylation. Thus, while LRRK1 activation was not fully dependent on LRRK2, its level of activation was impacted.

We noted that LRRK2 activation correlated with increased recruitment of Rab7 to Rab5 immunoprecipitates (Supplementary Fig. 5v), suggesting that LRRK2 effects on Rab7 occur on Rab5-positive membranes. To further examine the relationship between LRRK1 and LRRK2, we expressed endosomally targeted LRRK2 in N2a cells and performed LRRK2 immunoprecipitation, which revealed the presence of LRRK1 (Supplementary Fig. 5h). To determine whether PFF exposure modulates the association of LRRK1 and LRRK2 in neurons, we performed LRRK1 immunoprecipitation from lysates of primary neurons treated with PBS or PFF. Both LRRK1 and LRRK2 were detected in these immunoprecipitates, and notably, their activity was enhanced following PFF treatment (Fig. 6d). Together, these data indicate that both LRRK1 and LRRK2 are implicated in PFF-induced Rab7 phosphorylation. The findings are consistent with LRRK1 acting as the Rab7 kinase, while supporting a role for LRRK2 in regulating the level of LRRK1 activation and, consequently, Rab7 phosphorylation. We speculate that a complex containing LRRK1 and LRRK2 may facilitate or stabilize LRRK1 access to Rab7. Whether or not there is a direct interaction between the two kinases, our data indicate that LRRK2 activation functionally cooperates with LRRK1.

To further characterize Rab5 and Rab7 activation following PFF treatment, we performed GTP pull-down assays to examine whether their phosphorylated forms accumulate in the GTP-bound state. GTP pull-down studies showed increases in both phosphorylated Rab5 and Rab7 in PFF-treated neurons compared to PBS, and these increases were prevented by LRRK2 inhibition (Fig. 6e). We conclude that phosphorylated Rab5 and Rab7 are present in their GTP-bound forms in the setting of PFF-induced LRRK2 activation.

### LRRK2 inhibition rescues PFF-induced changes in ELN phenotypes, chromatin accessibility, gene expression and abnormal synaptic activity

**Fig. 7:**
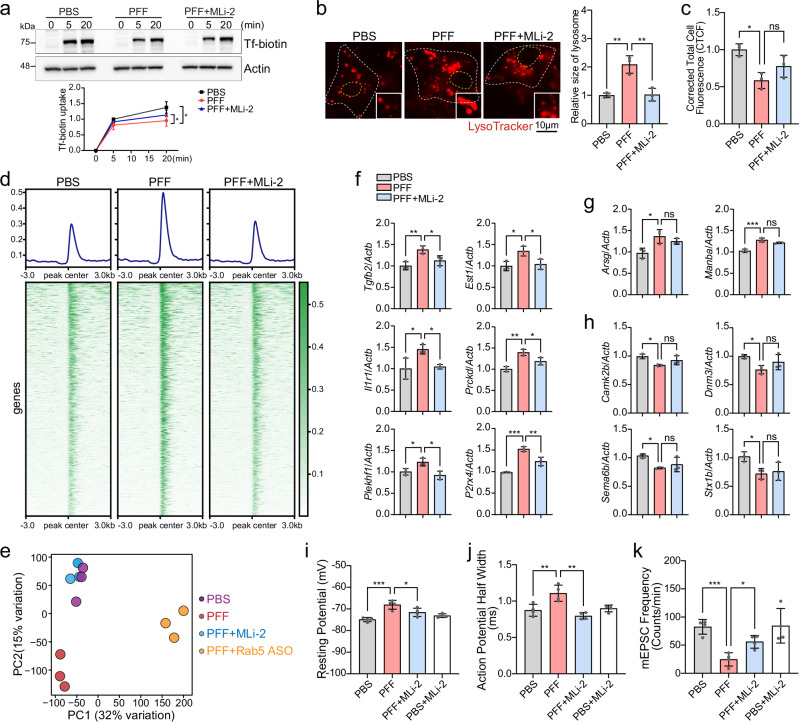
Rescue of ELN phenotypes, chromatin accessibility, gene expression, and synaptic activity by LRRK2 inhibition after PFF treatment. **a** Primary cortical neurons were treated with PBS, PFF, or PFF in combination with the LRRK2 inhibitor MLi-2. Endocytic activity was evaluated by incubating cells with 10 µg/ml biotinylated transferrin (Tf-biotin) for the indicated durations, followed by detection of internalized Tf-biotin using HRP-conjugated streptavidin. Representative image from *n* = 4 independent experiments. Quantification of transferrin uptake was performed at the indicated time points. Data are presented as mean ± SD (*n* = 4); one-way ANOVA followed by Tukey’s post hoc test; *p < 0.05. **b** Representative live-cell imaging of lysosome using LysoTracker Red DND-99 in mouse primary cortical neurons treated with PBS, PFF, or PFF plus MLi-2. Quantification of lysosomal size was based on LysoTracker fluorescence. >30 cells were analyzed per condition. Data are presented as mean ± SD from *n* = 3 independent experiments. Statistical significance was determined by one-way ANOVA followed by Tukey’s post hoc test; **p < 0.01. **c** Quantitative analysis of corrected total cell fluorescence (CTCF) from DQ™ Red BSA imaging in neurons treated with PBS, PFF, and PFF plus MLi-2. >30 cells were analyzed per condition. Data are presented as mean ± SD (*n* = 3); one-way ANOVA followed by Tukey’s post hoc test; ns, not significant; *p < 0.05. **d** Heatmaps display chromatin accessibility around the center of differential ATAC-seq peaks, extending 3 kb upstream and downstream. Upper panels show the average ATAC-seq signal across all these regions for each treatment condition. Analyses based on ATAC-seq data from *n* = 3 independent experiments. **e** Principal component analysis (PCA) of ATAC-seq peaks in neurons treated with PBS, PFF, or PFF combined with either MLi-2 or Rab5 ASOs. Analyses based on ATAC-seq data from *n* = 3 independent experiments. **f** RT-qPCR analysis in neurons treated with PBS, PFF, or PFF plus MLi-2, focusing on representative genes involved in lysosomal and senescence pathways that show PFF-induced chromatin opening and transcriptional upregulation, with chromatin accessibility changes reversible by MLi-2 treatment. Data are presented as mean ± SD (*n* = 3); one-way ANOVA followed by Tukey’s post hoc test; *p < 0.05, **p < 0.01, ***p < 0.001. **g** RT-qPCR analysis in neurons treated with PBS, PFF, or PFF plus MLi-2, focusing on genes that were transcriptionally upregulated upon PFF treatment but did not exhibit corresponding changes in chromatin accessibility. Data are presented as mean ± SD (*n* = 3); one-way ANOVA followed by Tukey’s post hoc test; ns not significant; *p < 0.05, ***p < 0.001. **h** RT-qPCR analysis in neurons treated with PBS, PFF, or PFF plus MLi-2, focusing on neuronal function-related genes showing PFF-induced open chromatin but transcriptional downregulation, with chromatin accessibility changes reversible by MLi-2 treatment. Data are presented as mean ± SD (*n* = 3); one-way ANOVA followed by Tukey’s post hoc test; ns not significant; *p < 0.05. **i** Resting membrane potentials of primary cortical neurons treated with PBS, PFF, PFF plus MLi-2 or PBS plus MLi-2. Data are presented as mean ± SD from *n* = 4 independent recordings per condition. Statistical significance was determined by one-way ANOVA followed by Tukey’s post hoc test; *p < 0.05, ***p < 0.001. **j** Action potential half-widths measured from primary cortical neurons under the same treatment conditions as in (**i**), using whole-cell patch-clamp recordings. Data are presented as mean ± SD (*n* = 4); one-way ANOVA followed by Tukey’s post hoc test; **p < 0.01. **k** Frequencies of AMPA receptor-mediated miniature excitatory postsynaptic currents (mEPSC) exhibited in primary cortical neurons treated as in (**i**). mEPSC frequency was expressed as events per minute. Data are presented as mean ± SD (*n* = 4); one-way ANOVA followed by Tukey’s post hoc test; *p < 0.05, ***p < 0.001.

To determine whether LRRK2 activity contributes to the phenotypic alterations induced by PFF, we examined the ability of MLi-2 to rescue key endolysosomal defects. Quantitative analysis showed that MLi-2 treatment significantly alleviated the PFF-induced impairment in transferrin internalization (Fig. 7a), suggesting that LRRK2 activation underlies deficits in Rab5-mediated uptake. Live-cell imaging further demonstrated that MLi-2 normalized the enlarged lysosomal morphology observed in PFF-treated neurons (Fig. 7b). In addition, while PFF significantly suppressed lysosomal proteolytic activity as measured by DQ-BSA fluorescence, MLi-2 co-treatment restored activity to near-baseline levels, although the change did not reach statistical significance (Fig. 7c and Supplementary Fig. 7a). These findings suggest that LRRK2-mediated phosphorylation of Rab5 impairs its function and disrupts early endosomal trafficking.

We next examined whether LRRK2 inhibition also reverses the chromatin accessibility alterations elicited by PFF, as revealed by ATAC-seq. In PFF-treated neurons, MLi-2 restored a large fraction of these changes, including both regions with increased and decreased accessibility (Fig. 7d and Supplementary Fig. 7d, e). Principal component analysis (PCA) showed that MLi-2 shifted the PFF-induced chromatin profiles closer to PBS controls along the PC2 axis (Fig. 7e). RT-qPCR was used to examine the expression of key genes whose products are linked to lysosomal or senescence pathways and that showed PFF-induced increases in both chromatin accessibility and mRNA levels. MLi-2 treatment restored the expression of these genes to levels comparable to those in PBS-treated controls (Fig. 7f and Supplementary Fig. 7f). In contrast, the genes that were upregulated without corresponding changes in chromatin accessibility were not rescued by MLi-2 (Fig. 7g), providing evidence that transcriptional rescue was linked to restored chromatin accessibility. Interesting, several downregulated genes related to neuronal function did not show restored mRNA levels despite normalized chromatin structure (Fig. 7h and Supplementary Fig. 7g), suggesting that additional regulatory mechanisms beyond chromatin structure contribute to sustained repression following PFF treatment.

Finally, we asked whether the LRRK2 inhibition could alleviate PFF-induced neuronal dysfunction at the physiological level. To assess neuronal function, we performed patch-clamp recordings on cultured cortical neurons treated with PFF or PBS, with or without MLi-2. Passive membrane properties, including capacitance and input resistance, were unchanged following 7 days of PFF exposure and 1–7 h of recovery. However, PFF-treated neurons exhibited a sustained depolarization of resting membrane potential by more than 6 mV compared to PBS controls, an effect that was reversed by MLi-2 co-treatment (Fig. 7i). PFF also significantly increased the half-width of action potentials, indicative of altered membrane excitability, and this phenotype was likewise normalized by MLi-2 (Fig. 7j). Although the amplitudes and kinetics of AMPA receptor-mediated mEPSCs were unaffected, the mEPSC frequencies were reduced by over 50% in the PFF group (Fig. 7k and Supplementary Fig. 7h), indicating impaired excitatory synaptic activity. Importantly, MLi-2 significantly restored mEPSC frequency, indicating that inhibition of LRRK2 rescues both intrinsic excitability and synaptic activity compromised by PFF exposure.

### Rab5 ASO partially reverses PFF-induced chromatin changes

Given the changes in phosphorylation of Rab5 and its interactome in PFF treated neurons, we next asked if reducing the levels of Rab5 would reverse PFF-mediated phenotypes. Primary cortical neurons were treated with antisense oligonucleotides (ASOs) targeting both Rab5a and Rab5b, the Rab5 isoforms most abundantly expressed in neurons, at final concentrations of 300 nM each (600 nM total) for three days. These ASOs effectively reduced Rab5a and Rab5b mRNA levels by ~70–90%, and protein levels by 50–60% (Supplementary Fig. 7b, c). ATAC-seq analysis revealed that Rab5 knockdown partially restored chromatin accessibility. Rab5 ASO-treated samples were positioned between the PBS and PFF-treated groups along the PC2 axis but showed a distinct shift along PC1 (Fig. 7e), suggesting that Rab5 loss not only mitigates PFF-induced chromatin remodeling but may also trigger broader changes in accessibility. Interestingly, while Rab5 ASOs significantly induced changes in open chromatin they had little to no effect on closed chromatin (Supplementary Fig. 7d, e). The extent to which common mechanisms underlie changes due to LRRK2 activation and Rab5 leading to chromatin remodeling is yet to be clarified. Nevertheless, these findings provide evidence that Rab5 contributes to the cellular changes induced by PFF treatment.

To further define the functional relevance of Rab5 phosphorylation in this context, we next examined whether phospho-state-specific Rab5 mutants could phenocopy or counteract PFF-induced effects. Using lentiviral expression of WT Rab5, the phospho-mimetic S84D mutant, or the phospho-dead S84A mutant in primary neurons, we first confirmed comparable expression levels of the three Rab5 variants (Supplementary Fig. 8a). We then assessed phosphorylation-dependent changes in Rab5 effector interactions by GFP-Rab5 co-immunoprecipitation. Under basal (PBS) conditions, Rab5 S84D showed increased association with Rab7 and CCZ1 compared with WT Rab5, whereas Rab5 S84A exhibited reduced binding to both proteins (Supplementary Fig. 8b). We next examined these interactions following a 2 h PFF exposure and observed a similar pattern: relative to WT Rab5, Rab5 S84D further enhanced Rab5 interaction with Rab7 and CCZ1, while Rab5 S84A attenuated these interactions (Supplementary Fig. 8c). Consistent with these effects, Rab5 S84D exacerbated PFF-induced lysosomal phenotypes, including increased lysosome size, while Rab5 S84A attenuated these changes (Supplementary Fig. 8d). DQ-BSA fluorescence measurements showed similar directional trends, although these differences did not reach statistical significance (Supplementary Fig. 8e). Finally, Rab5 phosphorylation state also influenced downstream transcriptional responses following PFF exposure, with Rab5 S84D generally enhancing, and Rab5 S84A reducing, PFF-responsive mRNA changes associated with chromatin remodeling (Supplementary Fig. 8f). Together with the Rab5 ASO rescue experiments, these findings provide functional evidence that Rab5, and specifically phosphorylated Rab5, play a critical role in mediating PFF-induced ELN remodeling and downstream chromatin-associated responses.

## Discussion

PD is a progressive neurodegenerative disorder lacking disease-modifying therapies, with a global burden that continues to rise in parallel with population ageing^55,56^. Hallmark features of PD include pathological aggregation of α-synuclein and dysfunction of the endolysosomal system^57^. We asked if the two are mechanistically linked. Using a neuronal model of α-synuclein pathology induced by PFFs, we documented a multifaceted cascade of cellular dysfunction. Central to this cascade was rapid activation and recruitment of LRRK2 to early endosomes, where it phosphorylates Rab5; this finding indicates that Rab5 is a substrate in the context of PFF exposure. This post-translational modification altered Rab5-dependent vesicular components, disrupted endolysosomal function, and initiated widespread epigenomic and transcriptional reprogramming. Our findings support the LRRK2-Rab5 axis as a key pathological driver and nominate this pathway as a promising target for therapeutic intervention.

PFF treatment induced profound remodeling of neuronal lysosomes, as evidenced by increased lysosomal size and marked alterations in protein composition. Proteomic analysis revealed the accumulation of lysosomal membrane proteins (LAMP1/2) and hydrolases (Cathepsins B and C), along with non-canonical substrates such as ribosomal and nuclear proteins, indicative of substrate overload and trafficking defects. Despite these increases, overall proteolytic activity declined, pointing to a disconnect between enzyme abundance and function. Furthermore, redistribution of lysosomal proteins into cytosolic fractions pointed to compromised membrane compartmentalization, although lysosomal pH remained unchanged (Supplementary Fig. 1n). Increased phosphorylated mTOR (p-mTOR), a key autophagy suppressor, suggests that blockade of autophagic initiation may contribute to the accumulation of undegraded substrates and disrupted proteostasis. These findings align with previous studies showing that α-synuclein aggregates impede autophagosome-lysosome fusion and lysosomal clearance^58,59^. Our organelle-resolved proteomic analysis expands upon this, revealing membrane destabilization and a decoupling of enzyme abundance from function, thus highlighting underappreciated aspects of lysosomal pathology in response to PFF exposure.

Lysosomal dysfunction was accompanied by transcriptional upregulation of lysosomal genes and nuclear translocation of TFEB and TFE3, key transcription factors that coordinate the lysosomal-autophagy program. We observed reduced phosphorylation of TFEB, consistent with nuclear localization. ATAC-seq revealed widespread chromatin remodeling, including increased accessibility at promoter regions of TFEB target genes and other loci beyond canonical lysosomal function. For example, Phafin1 (PLEKHF1), a PH/FYVE-domain protein involved in endosomal remodeling and lysosome targeting^60^, showed coordinated promoter opening and transcriptional upregulation, consistent with the presence of predicted TFEB-binding motifs. These findings suggest that PFF exposure initiates both a lysosome-specific transcriptional program and broader chromatin accessibility changes that mediate adaptive or maladaptive transcriptional responses. Supporting this, nuclear fractionation revealed reduced Lamin B1 protein levels, a feature associated with nuclear lamina disorganization and stress-induced senescence, consistent with prior reports in senescent astrocytes and aging neural stem/progenitor cells^61,62^.

A key finding of this study is the induction of a senescence-like transcriptional program in neurons following PFF-induced lysosomal stress. Notably, several hallmark senescence genes, including *Tgfb2*, *Chek2*, and *Ets1*, were significantly upregulated, implicating converging stress-response pathways involving oxidative stress, DNA damage, and inflammatory signaling^63,64^. In particular, ETS1 was recently identified as a key driver of senescence-like signatures in Alzheimer’s disease patient-derived induced neurons (iNs), where it regulates *CDKN2A* (p16) and pro-inflammatory gene expression^65^, underscoring a conserved role in neuronal stress responses. Interestingly, however, *Foxm1*, a transcriptional regulator that counters senescence by promoting cell cycle progression and stress resilience^66^, was also upregulated, suggesting that PFF treatment induces a transitional, potentially reversible senescence-like regulatory state. Supporting this, we observed weak SA-β-gal staining, minimal SASP gene induction, and a lack of transposable elements activation (L1, ALU, SVA), and no significant reduction in global H3K9me3 levels, features that typically characterize late-stage, irreversible senescence^67^. Together, these results suggest that PFF-induced lysosomal stress elicits a dynamic, non-terminal senescence-associated program in neurons, potentially enabling recovery if compensatory pathways like FOXM1 can be activated. Understanding this transitional state may provide opportunities to modulate neuronal fate and resilience under proteotoxic stress.

While chromatin accessibility is typically linked to transcriptional activation, as shown in canonical senescence models^68^, our integrative ATAC-seq and RNA-seq analysis in PFF-treated neurons revealed a paradoxical pattern: increased accessibility was observed in both upregulated and downregulated genes, with a stronger association in the latter. Up-peaks linked to upregulated genes were enriched in promoter-proximal regions, whereas those associated with downregulated genes were more frequently found in intronic and distal intergenic regions, indicating a shift toward distal regulatory elements. This spatial bias suggests that PFF-induced chromatin remodeling may preferentially affect distal regulatory elements whose function becomes compromised. One plausible mechanism is a disruption of long-range enhancer-promoter interactions due to altered three-dimensional genome topology, such as weakened topologically associated domain (TAD) boundaries or aberrant loop formation^69^. Under neurotoxic stress, accessible distal enhancers may fail to engage with their target promoters, rendering them transcriptionally ineffective despite remaining open. Additionally, chromatin opening in these regions may not represent active regulation but rather passive heterochromatin decompaction, a hallmark of nuclear expansion and architectural disorganization observed in aged cortical neurons^70^. These physical changes can redistribute regulatory sequences distant from transcriptional hubs or lamina-associated domains, thereby altering their functional context.

Our focus on the lysosome followed on the findings in earlier reports that highlight lysosomal changes induced by PFF treatment^71,72^. To further explicate endolysosomal dysregulation we examined upstream events regulators and identified marked changes in Rab5. Despite the increased GTP-bound Rab5, its function was impaired, as evidenced by reduced endocytic uptake. This paradox was explained by LRRK2-mediated phosphorylation of Rab5 on early endosomes, leading to a remodeled Rab5 interactome with respect to both canonical and non-canonical effectors. Rab GTPase modulation by LRRK2 is well-characterized and has been extensively reviewed^73^. Its relevance for Rab5 has been demonstrated herein. Concurrent Rab7 phosphorylation, via LRRK2-LRRK1 functional crosstalk, further suggests widespread endosomal remodeling. Thus, LRRK2 activation reprograms Rab5 and Rab7 activity, reshaping vesicle identity and propagating endolysosomal dysfunction.

To place the alterations in early endosomes within the temporal framework of endolysosomal dysregulation we examined the early time course of PFF-induced changes. Using magnetic particle-based isolation optimized with an early capture time (8 min), we enriched a population of Rab5-positive early endosomes. Under basal (0 min PFF) conditions, our preparation contained predominantly Rab5, but also detectable amounts of Rab7 and Rab11. The presence of these Rabs is consistent with the capture of a small population of transitional Rab5-positive endosomes progressing toward Rab7- or Rab11- positive states. Remarkably, within 30 min of PFF addition there was LRRK2 activation and Rab5 phosphorylation in the early endosome, denoting rapid involvement of this compartment in the cellular response. Early endosomes accumulated not only Rab5 but also Rabs 7 and 11, along with regulatory effectors of both Rab5 and Rab7, further supporting the early endosome as an early and possibly initial hub for PFF-induced cellular remodeling. Interestingly, LRRK2 was also detected on lysosomes at this early stage, prior to PFF arrival, raising the possibility of a signal transmitted from early endosomes to lysosomes. In this regard, we observed early endosomal recruitment of Rabaptin-5, a known GEF for Rab29^74^. Rab29, in turn, has been shown to recruit and activate LRRK2 at lysosomes^75,76^. One speculation is that PFF-induced signaling at early endosomes engages Rabaptin-5 to activate Rab29, subsequently promoting LRRK2 activation at the lysosome, thereby linking changes in early endosome remodeling to downstream lysosomal responses. We envision the possibility that rapid recruitment of LRRK2 to both early endosomes and lysosomes may together mediate changes in endolysosomal function and downstream changes in nuclear and neuronal function.

The discovery of an LRRK2-pRab5 axis integrates cell biological mechanisms with genetic insights in PD. In addition to highlighting the involvement of α-synuclein and LRRK2, the axis opens the possibility that changes in early endosomes also implicate the gene products of *VPS35*, *SYT4*, and *ATP6V0A1*, all of which are recognized PD risk alleles^45^. Of note, variants in the *GBA* gene represent the most common risk factor for PD. The increase in GBA levels associated with Rab5-positive early endosomes may further inform our understanding of the pathogenic events induced by mutant GBA.

Pharmacological inhibition of LRRK2 with MLi-2 reversed multiple PFF-induced phenotypes, including Rab5 phosphorylation, lysosomal structural alterations, chromatin remodeling, and the upregulation of senescence-associated genes. At the functional level, MLi-2 restored neuronal excitability, as evidenced by improved resting membrane potential and mEPSC frequency. Notably, however, the transcriptional repression of several neuronal genes persisted despite normalization of chromatin accessibility, highlighting a dissociation between epigenetic remodeling and gene activation. These findings suggest that while LRRK2 inhibition can effectively rescue key aspects of PFF-induced structural and physiological disruption, certain PFF-induced transcriptional changes may reflect LRRK2-independent or irreversible programs. Together, these results indicate that LRRK2 activation is necessary, but not fully sufficient for mediating the full spectrum of PFF-induced neuronal dysfunction. Nevertheless, its central role in initiating early pathological changes highlights LRRK2 as a promising therapeutic target for early intervention in Parkinson’s disease. It is noteworthy that this therapeutic approach is currently being evaluated in clinical trials, including a small-molecule kinase inhibitor DNL151 (BIIB122, developed by Denali and Biogen) and an antisense oligonucleotide ION859 (BIIB094, developed by Ionis and Biogen)^77^.

Finally, our results demonstrate that dysregulated Rab5 activity plays a mechanistic role in PFF-induced epigenomic remodeling. Knockdown of Rab5 via ASOs partially restored chromatin accessibility, particularly at loci associated with stress responses and vesicle trafficking. However, the extent and pattern of rescue differed from those observed with LRRK2 inhibition, as PCA revealed a distinct chromatin landscape with limited reversal of repressive changes. These findings suggest that while Rab5 is a critical downstream target of LRRK2, reducing the levels of the protein alters chromatin architecture through some effects beyond those driven by LRRK2-mediated phosphorylation. Thus, while Rab5 dysregulation contributes to disease pathogenesis, selectively targeting it may not suffice to normalize function, underscoring a hierarchical role for LRRK2 in orchestrating chromatin and functional outcomes.

To exclude the possibility that the lysosomal alterations observed following PFF treatment reflected nonspecific membrane rupture or leakage, lysosomes were isolated using magnetic beads under gentle mechanical conditions to preserve compartmental integrity. Evidence supporting preserved lysosomal integrity was the absence of leakage of the soluble lysosomal enzyme Cat B or the membrane protein LIMP2. Several complementary measures supported this conclusion. pHrodo Red revealed no significant change in lysosomal acidity following PFF treatment. In addition, under the conditions of our experiments imaging of galectin-3 did not exhibit punctate lysosomal recruitment. Importantly, cytosolic cathepsin L activity also remained unchanged in PFF-treated neurons compared with PBS controls. Despite preserved membrane integrity, proteomic profiling revealed substantial alterations in lysosomal and cytoplasmic proteins following PFF exposure. We observed both retention and depletion of specific protein classes, including enrichment of endolysosomal proteins, ribosomal components, and RNA processing machinery in the lysosomal fraction, alongside a reduction of oxidative phosphorylation-associated and ER-associated proteins. These bidirectional shifts likely reflect the redistribution of cellular components due to impaired trafficking dynamics. Furthermore, PFF exposure revealed Rab5 interactions with ESCRT components CHMP6 and CHMP4B, pointing to involvement of membrane remodeling or repair.

**Fig. 8:**
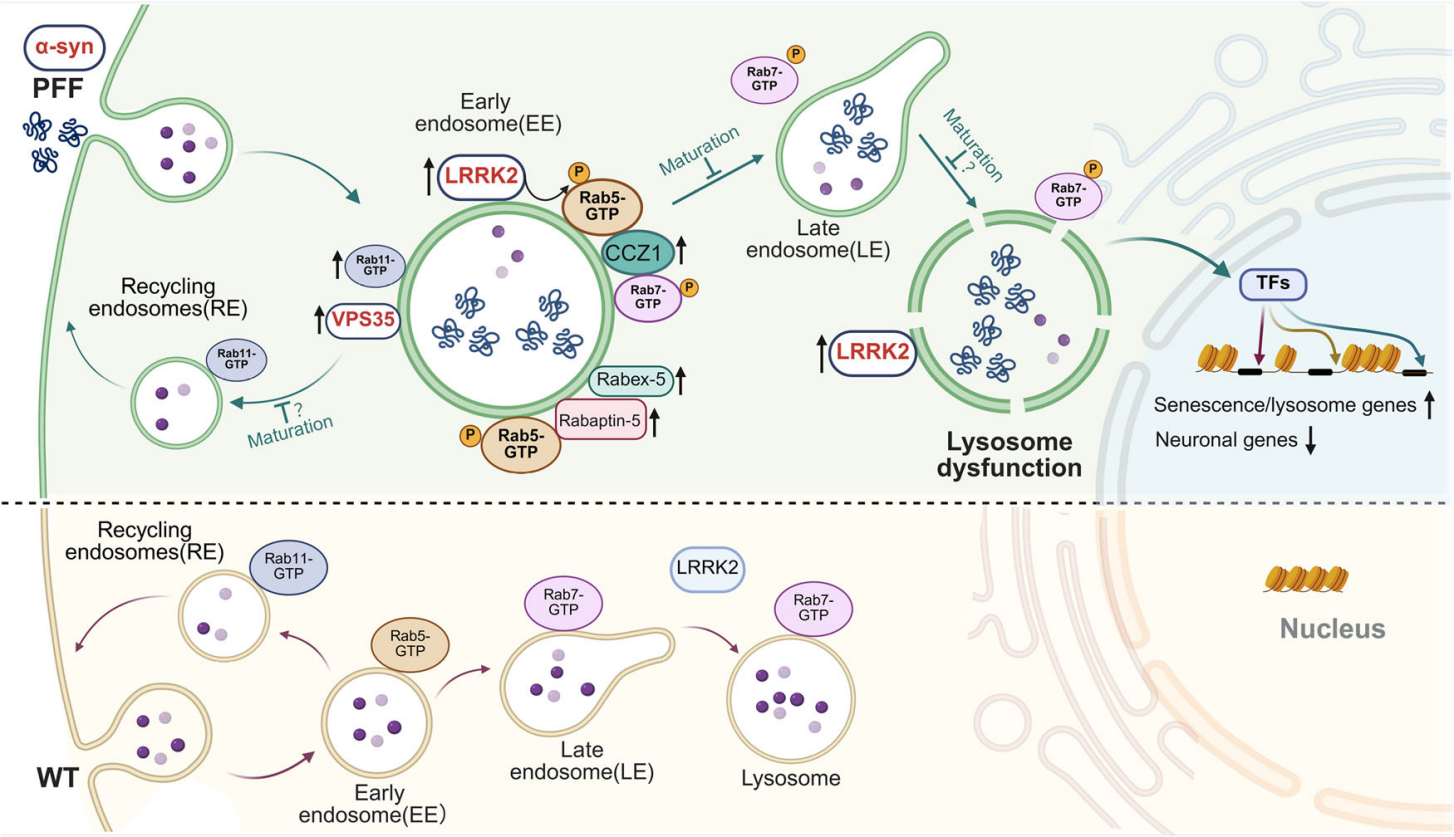
Schematic model of LRRK2-Rab5-mediated endolysosomal impairment and neuronal dysfunction upon PFF exposure. PFF exposure induces LRRK2 upregulation and recruitment to early endosomes (EEs), where it phosphorylates Rab5 and reprograms the Rab5 interactome, including increased association with Rabaptin-5, Rabex-5, CCZ1, and VPS35. These alterations impair endosomal maturation, recycling, and lysosomal function, accompanied by phosphorylated Rab7 and defective vesicle trafficking. Resulting cellular stress activates TFEB/TFE3 and other transcription factors, leading to chromatin remodeling, upregulation of lysosomal and senescence-associated genes, and repression of neuronal genes. Parkinson’s disease (PD) key regulators α-synuclein, LRRK2 and VPS35 are highlighted in red text with black boxes. Figure created with BioRender.com.

Collectively, our findings define a mechanistic model in which PFF exposure triggers LRRK2 recruitment to early endosomes, leading to Rab5 phosphorylation. Remarkably, the resulting changes in early endosome proteins mark the disruption of the endosomal Rab cascade during which Rab5 recruitment of the GEFs for downstream Rabs normally initiates discharge of Rab5 to allow conversion of the early endosomes to either late endosomes or recycling endosomes. The failure to properly discharge Rab5 from early endosomes in the PFF condition can be readily envisioned to disrupt endolysosomal trafficking and lysosomal dysfunction. That the attendant senescence-associated transcriptional remodeling can be linked to ELN disruption is supported by its reversal following LRRK2 inhibition. As summarized in Fig. 8, we envision the LRRK2-Rab5 axis to serve as an early, central hub linking vesicular and genomic stress responses. The findings elucidate how α-synuclein pathology impairs neuronal homeostasis and identify LRRK2 and Rab5 as potentially actionable targets for therapeutic intervention in PD and related neurodegenerative conditions.

## Methods

### Animal ethics

All animal procedures were conducted in strict accordance with the Guide for the Care and Use of Laboratory Animals of the National Institutes of Health and were approved by the UC San Diego Institutional Animal Care and Use Committee (IACUC; Protocol No. S09315). Mice were bred and maintained under standard conditions, housed two to five per cage on a 12-h light/dark cycle, and provided ad libitum access to food and water. Timed-pregnant females were deeply anesthetized with isoflurane and euthanized by anesthetic overdose in accordance with UCSD IACUC guidelines; embryos (E16–E18) were immediately collected for primary cortical neuron preparation. Each pregnancy (yielding approximately nine embryos) was treated as one independent biological replicate; neuronal cultures derived from each pregnancy were randomly assigned to experimental groups. All animal studies were conducted and reported in accordance with the ARRIVE guidelines.

### Cell culture

Mouse neuroblastoma cell line N2a (ATCC; Cat# CCL-131) and human embryonic kidney cell line HEK293T (ATCC; Cat# CRL-3216) were cultured in DMEM medium (Gibco) supplemented with 10% FBS (Omega Scientific; Cat# FB-02) and 1% Penicillin-Streptomycin (Gibco). All the cell lines were maintained at 37 °C and 5% CO_2_ and were regularly tested for mycoplasma contamination.

Primary cortical neurons were prepared from embryonic day 16–18 embryos dissected from pregnant female mice (C57BL/6; The Jackson Laboratory). The pups were removed from the amniotic sacs and immediately transferred to cold PBS plus penicillin and Streptomycin. Cortical tissues were dissected, minced with a sterile, fire-polished Pasteur pipette, and gently triturated. The tissue was then digested with 0.25% trypsin (Thermo Fisher Scientific; Cat# 15090046) diluted in HBSS (Thermo Fisher Scientific; Cat# 14185052) at 37 °C water bath for 10 min, followed by treatment with 1 mg/ml DNase I (Roche; Cat# 10104159001). After further trituration to obtain single-cell suspensions, cells were neutralized with Neurobasal medium (Thermo Fisher Scientific; Cat# 21103049) supplemented with 10% FBS, B-27 Supplement (Thermo Fisher Scientific; Cat# 17504044), and GlutaMAX Supplement (Thermo Fisher Scientific; Cat# 35050061). The mixture was filtered with a 40-μm cell strainer (FALCON), and the cells were harvested by centrifugation and centrifuged at 1000 rpm for 5 min at room temperature. The neuronal pellet was resuspended and counted, then plated onto poly-D-lysine (Millipore Sigma; Cat# P6407)-coated plates with seeding medium (Neurobasal medium supplemented with 10% FBS, B-27, and GlutaMAX). Four hours after seeding, the medium was replaced with maintenance medium (Neurobasal with B-27 and GlutaMAX), and half of the medium was refreshed every 2–3 days until further use.

Under these standard culture conditions, primary cortical neuron cultures exhibited minimal glial contamination, with GFAP-immunopositive cells typically representing ≤1% of the total cell population at DIV14, while MAP2-positive neurons were abundant.

For neuron-enriched cultures, 1 μM Ara-C (cytosine β-D-arabinofuranoside; MilliporeSigma; Cat# C3350000) was added to the culture medium for the first 48 h after plating to suppress proliferation of non-neuronal cells. Ara-C was then removed, and cultures were maintained in standard neuronal maintenance medium before experimental treatments.

### Treatment of neurons with α-synuclein and MLi-2

Mouse primary cortical neurons were treated at DIV7 with human wild-type α-synuclein preformed fibrils (PFFs) (StressMarq Biosciences; Cat# SPR-322). PFFs were commercially obtained and supplied with manufacturer-provided quality control documentation, including endotoxin testing (low endotoxin, <5 EU/mL at 2 mg/mL). Prior to treatment, PFFs were diluted in sterile PBS to the working concentration (100 μg/ml) and sonicated immediately using a pulse protocol to generate short fibrillar fragments, as described previously. Sonication was performed in an on/off cycle mode (10 s on, 5 s off) for a total duration of 1 min. The sonicated PFFs were then added directly to the culture medium at a final concentration of 1 μg/mL and incubated for 7 days. Neurons treated with an equal volume of PBS served as vehicle controls. In parallel, human α-synuclein monomers (StressMarq Biosciences; Cat# SPR-321), supplied with manufacturer quality control and endotoxin certification, were applied at the same final concentration (1 μg/mL) as an additional control condition.

The LRRK2 kinase inhibitor MLi-2 (BOC Sciences; Cat# 1627091-47-7) was used at a final concentration of 600 nM. This dosing regimen was based on a previous study^78^, and independently validated in our primary cortical neurons by dose-response analyses, which confirmed that 600 nM MLi-2 effectively inhibited LRRK2-dependent phosphorylation without detectable effects on neuronal morphology or viability. An equal volume of vehicle (DMSO) was added to control cultures. To assess whether pharmacological inhibition of LRRK2 rescues PFF-induced phenotypes, neurons were treated with MLi-2 starting at DIV9 (2 days after PFF exposure) and maintained in culture for 5 days at the same final concentration of 600 nM, with vehicle (DMSO) as control.

### Western blotting analysis

Proteins were analyzed by Western blotting using either equal amounts of total protein or equal volumes of subcellular fraction samples. Samples were resolved by SDS-PAGE and transferred onto PVDF membranes (Bio-Rad). Membranes were blocked in 5% nonfat milk for 1 h and then incubated with the indicated primary antibodies. Signal detection was performed using the Clarity Western ECL substrate (Bio-Rad), and images were acquired with the ChemiDoc XRS+ imaging system (Bio-Rad). Quantification was carried out using ImageLab 6.0 software (Bio-Rad), but only for blots with signals within the linear dynamic range.

Antibodies used for Western blotting are detailed below: Mouse monoclonal anti-α-Synuclein (BD Biosciences; Cat# 610786), Rabbit monoclonal anti-α-Synuclein (Cell Signaling Technology; Cat# 4179), Rabbit monoclonal anti-Phospho-α-Synuclein (Ser129) (D1R1R) (Cell Signaling Technology; Cat# 23706), Mouse monoclonal anti-Beta Actin (Proteintech; Cat# 60008-1-Ig), Rabbit Polyclonal anti-beta III Tubulin (Abcam; Cat# ab18207), Mouse monoclonal anti-Bovine Serum Albumin (BSA) (Proteintech; Cat# 66201-1-Ig), Rabbit monoclonal anti-cathepsin B (Cell Signaling Technology; Cat# 31718), Mouse monoclonal anti-Cathepsin C(D-6) (Santa Cruz Biotechnology; Cat# sc-74590), Mouse monoclonal anti-CCZ1(Santa Cruz Biotechnology; Cat#sc-514290), Rabbit Polyclonal anti-CHEK2 (Proteintech; Cat# 13954-1-AP), Rabbit Polyclonal anti-Cyclin B2 (Proteintech; Cat# 21644-1-AP), Rabbit polyclonal anti-HSP70 (Proteintech; Cat# 10995-1-AP), Mouse monoclonal anti-Iduronate 2 sulfatase (IDS) (Proteintech; Cat# 66112-1-Ig), Rabbit Polyclonal anti-Lamin B1 (Proteintech; Cat# 12987-1-AP), Rabbit monoclonal anti-LAMP1 (Cell Signaling Technology; Cat# 99437), Mouse monoclonal anti-LAMP2 (Proteintech; Cat# 66301-1-Ig), Rabbit monoclonal anti-LIMP-2 (Cell Signaling Technology; Cat# 27960), Rabbit monoclonal anti-LRRK2 (Abcam; Cat# ab133474), Rabbit monoclonal anti-LRRK2 (phospho S935) (Abcam; Cat# ab133450), Rabbit monoclonal anti-LRRK2 (phospho S1292) (Abcam; Cat# ab203181), Rabbit Polyclonal anti-LRRK1 (Thermo Fisher Scientific; Cat# PA5-13868), Rabbit Polyclonal anti-MANBA (Thermo Fisher Scientific; Cat# PA5-90351), Rabbit Polyclonal anti-mTOR (Cell Signaling Technology; Cat# 2972), Rabbit Polyclonal anti-Phospho-mTOR (Ser2448) (Cell Signaling Technology; Cat# 2971), Mouse monoclonal anti-MYC tag (Proteintech; Cat# 60003-2-Ig), Rabbit monoclonal anti-Nucleolin (Cell Signaling Technology; Cat# 14574), Mouse monoclonal anti-p21 (Thermo Fisher Scientific; Cat# AHZ0422), Rabbit Polyclonal anti-Phosphoserine (Abcam; Cat# ab9332), Rabbit polyclonal anti-Phospho-(Ser) PKC Substrate (Cell Signaling Technology; Cat# 2261), Mouse monoclonal anti-Rab5 (Synaptic Systems; Cat# 108 011), Mouse monoclonal anti-Rab5a antibody (Santa Cruz Biotechnology; Cat# sc-46692), Mouse monoclonal anti-RAB5b (Santa Cruz Biotechnology; Cat# sc-373725), Rabbit polyclonal anti-Phospho-Rab5a (Ser84) (Affinity Biosciences; Cat# AF3706), Mouse Monoclonal anti-Rab7 (Cell Signaling Technology; Cat# 95746), Rabbit monoclonal anti-RAB7 (phospho S72) (Abcam; Cat# ab302494), Mouse Monoclonal anti-RAB10 (Abcam; Cat# ab307296), Rabbit monoclonal anti-RAB10 (phospho T73) (Abcam; Cat# ab230261), Mouse monoclonal anti-RAB11A (Proteintech; Cat# 67902-1-Ig), Mouse monoclonal anti-Rabaptin-5 (BD Biosciences; Cat# 610676), Mouse monoclonal anti-Rabex-5 (Santa Cruz Biotechnology; Cat# sc-166611), Rabbit monoclonal anti-TFE3 (Cell Signaling Technologies; Cat# 81744), Rabbit monoclonal anti-TFEB (Cell Signaling Technology; Cat# 83010), Rabbit monoclonal anti-Phospho-TFEB (Ser211) (Cell Signaling Technology; Cat# 37681), Rabbit monoclonal anti-4E-BP1 (Cell Signaling Technology; Cat# 9644), Rabbit monoclonal anti-Phospho-4E-BP1 (Thr37/46) (Cell Signaling Technology; Cat# 2855), Rabbit Polyclonal anti-TGF beta 2 (Proteintech; Cat# 19999-1-AP), Rabbit monoclonal anti-TMEM106B (Cell Signaling Technology; Cat# 93334), Rabbit Polyclonal anti-Vps35 (Proteintech; Cat# 10236-1-AP), Goat anti-rabbit IgG-HRP (Jackson ImmunoResearch Laboratories, Cat# 111-035-144); Goat anti-mouse IgG-HRP (Jackson ImmunoResearch Laboratories; Cat# 115-035-003), HRP-Conjugated Streptavidin (Thermo Fisher Scientific; Cat# N100).

### Plasmids transfection, ASO treatment, lentiviral expression constructs and shRNA delivery

2×Myc-LRRK2-WT (Addgene; Cat# 25361), EE-LRRK2 (Addgene; Cat# 193583), and GFP-RAB5 (available in our lab) were transfected into N2a cells using Lipofectamine™ 2000 (Thermo Fisher Scientific) following the manufacturer’s protocol. Cells were collected 36–48 h post-transfection for analysis.

Primary cortical neurons were treated with antisense oligonucleotides (ASOs) targeting both Rab5a and Rab5b (Integrated DNA Technologies), the Rab5 isoforms most abundantly expressed in the brain. ASOs were applied at final concentrations of 300 nM each (600 nM total) on DIV11. ASOs were diluted in fresh culture medium and directly added to neurons without transfection reagents. After 72 h of treatment, cells were collected for downstream analysis.

Human wild-type Rab5a was cloned into a lentiviral expression vector based on the pLenti CMV GFP Puro backbone (Addgene; Cat# 17448), generating a Lent-GFP-wt-human Rab5a construct. Serine 84 was mutated to alanine (S84A) or aspartate (S84D) using PCR-based site-directed mutagenesis. All constructs were sequence-verified by Sanger sequencing prior to lentiviral packaging and experimental use. LRRK1 shRNA plasmid (mouse) (Santa Cruz Biotechnology; Cat# sc-149115-SH) and LRRK2 shRNA plasmid (mouse) (Santa Cruz Biotechnology; Cat# sc-45750-SH) were purchased from Santa Cruz Biotechnology. Short hairpin RNAs targeting α-synuclein (shαSyn-1 and shαSyn-2) or luciferase (shLUC, as control) were cloned into the pLKO.1 puro vector (addgene; Cat# 8453) between the AgeI and EcoRI sites. The target sequences were 5’-AGAATCGTCGTATGCAGTGAA-3’ for shLUC, 5’-GCAGTGAGGCTTATGAAAT-3’ for shαSyn-1, and 5’-ACCAAAGAGCAAGTGACAAAT-3’ for shαSyn-2. Lentiviral particles were produced in HEK293T cells by co-transfecting the shRNA expression plasmids with the packaging plasmids pMD2.G (addgene; Cat#12259) and psPAX2 (addgene; Cat#12260) using Lipofectamine™ 2000. Viruses were harvested 48 h post-transfection, concentrated by ultracentrifugation at 24,000 rpm for 2 h at 4 °C using SW-28 rotor, resuspended in PBS (Ca^2+^/Mg^2+^-free), and applied to primary cultured neurons at DIV11. Cells were harvested at DIV14 for downstream analysis.

### Immunoprecipitation

N2a cells and mouse primary cortical neurons were used for immunoprecipitation (IP) experiments. Cells cultured in 10-cm plates were washed twice with cold PBS and harvested by centrifugation at 500 × *g* for 5 min at 4 °C. Cells were lysed in 1 ml of Pierce™ IP Lysis Buffer (Thermo Fisher Scientific; Cat# PI87787), supplemented with Halt™ Protease and Phosphatase Inhibitor Cocktail, EDTA-free (Thermo Fisher Scientific; Cat# 78445), on ice for 15 min. Lysates were clarified by centrifugation at 12,000 × *g* for 15 min at 4 °C, and a portion of the supernatant was retained as input. The remaining supernatant was incubated with the indicated antibody or control IgG on a rotator overnight at 4 °C, followed by incubation with protein G Dynabeads (Thermo Fisher Scientific; Cat# 10003D) for 2 h at 4 °C. Beads were washed twice with cold IP lysis buffer and twice with high-salt wash buffer (IP lysis buffer supplemented with 300 mM NaCl), and bound proteins were eluted by boiling in SDS-PAGE sample buffer. The following antibodies were used: Monoclonal anti-GFP tag (Proteintech; Cat# 66002-1-Ig), Mouse IgG control (Jackson ImmunoResearch Laboratories; Cat# 015-000-003), Rabbit Monoclonal Rab5 antibody (Abcam; Cat# ab218624), Rabbit IgG control (Proteintech; Cat# 30000-0-AP).

For mass spectrometry analysis of Rab5-interacting proteins in primary cortical neurons treated with PBS or PFF, immunoprecipitation was performed using the Pierce™ Classic Magnetic IP/Co-IP Kit (Thermo Fisher Scientific; Cat# 88804) according to the manufacturer’s instructions. After antibody coupling and IP enrichment, beads were extensively washed and directly submitted to the proteomics facility, where on-bead elution and LC-MS/MS analysis were performed.

### GTP-bound Rab pull-down assay

GTP-bound Rab proteins were examined as previously described^79^. Briefly, Primary neurons cultured in 10-cm plates were harvested and lysed in buffer containing 50 mM Tris-HCl (pH 7.5), 250 mM NaCl, 5 mM magnesium acetate, 0.5% Triton X-100, 0.2 mM sodium orthovanadate, and protease inhibitors. Lysates were rotated at 4 °C for 30 min and clarified by centrifugation at 12,000 rpm for 15 min at 4 °C. Protein concentrations were determined using the Bradford assay, and aliquots were reserved to assess total Rab protein levels. Equal amounts of lysates were incubated overnight at 4 °C with 100–150 µL of guanosine 5′-triphosphate (GTP)-agarose beads (Millipore Sigma; Cat# G9768) under gentle rotation. Beads were washed three times with lysis buffer (excluding protease inhibitors) and eluted by boiling in SDS-PAGE sample buffer. GTP-bound Rabs were subsequently detected by Western blotting.

### RNA isolation and quantitative PCR

Total RNA was extracted from cultured mouse cortical neurons using the Quick-RNA Miniprep Kit (Zymo Research; Cat# R1054). RNA concentration and purity were assessed using a Nanodrop spectrophotometer (Thermo Fisher Scientific). Equal amounts of RNA were reverse transcribed into cDNA using the High-Capacity cDNA Reverse Transcription Kit (Thermo Fisher Scientific; Cat# 4368814) according to the manufacturer’s instructions. Quantitative PCR (qPCR) was performed for 40 cycles using the Applied Biosystems 7300 Real-Time PCR System. Primer sequences were primarily obtained from PrimerBank and are listed in Supplementary Data 5. Gene expression was analyzed using the ΔΔCt method. A portion of the extracted total RNA was submitted to the IGM Genomics Center for ribosomal RNA-depleted RNA sequencing. Library preparation and sequencing were performed following the center’s standard protocols.

### Transferrin uptake assay

Mouse cortical neurons were cultured in 12-well plates. At DIV14, neurons were starved in Neurobasal medium for 2 hours, then incubated with 10 μg/mL biotin-conjugated transferrin (MilliporeSigma; Cat# T3915) for the indicated time periods. To remove surface-bound transferrin, cells were washed with glycine buffer (100 mM NaCl, 50 mM glycine, pH 3.0), followed by two rinses with cold PBS. Cells were subsequently lysed in buffer containing 50 mM Tris-HCl (pH 7.5), 150 mM NaCl, 1% Triton X-100, 1 mM PMSF, and Halt™ Protease and Phosphatase Inhibitor Cocktail for Western blot analysis.

### LysoTracker-based lysosome analysis

Mouse cortical neurons were cultured on poly-D-lysine-coated 35-mm glass-bottom dishes for two weeks. Cells were then incubated with 50 nM LysoTracker™ Red DND-99 (Thermo Fisher Scientific; Cat# L7528) in pre-warmed culture medium at 37 °C for 30 min. Following incubation, cells were washed with fresh culture medium, and live-cell imaging was performed immediately after staining using a Leica TCS SPE confocal microscope equipped with a 100× oil immersion objective. Images were acquired under identical laser and detector settings for all conditions. Lysosomal size and number were quantified using ImageJ (NIH), with particle analysis performed on thresholded images. For each culture, >10 neurons per condition were analyzed. Values were averaged within each culture to generate one biological replicate, and statistical analyses were performed on these biological replicates.

### Magnetic isolation of lysosomes and endosomes

Lysosomes and endosomes were magnetically isolated from cultured neurons as previously described^80^ with minor modifications. Neurons were plated on poly-D-lysine-coated 10-cm dishes at a density of 10 × 10⁶ cells per dish and incubated with DexoMAG 40 iron-dextran solution (10 mg/mL; Liquids Research; Cat# F2726) at a ratio of 1 mL per 10 mL of culture medium. For lysosome isolation, cells were incubated for 20 h. For endosome isolation, the incubation period was limited to 8 min.

After incubation, cells were rinsed twice with ice-cold PBS, collected by scraping, and pelleted by centrifugation at 300 × *g* for 5 min at 4 °C. Pellets were resuspended and lysed by mechanical shearing through a 25 G needle in SCA buffer (20 mM HEPES-KOH, pH 7.5, 10 mM KCl, 1.5 mM MgCl₂, 1 mM EDTA, 1 mM EGTA, 250 mM sucrose, supplemented with protease and phosphatase inhibitors). The lysates were centrifuged at 800 × *g* for 5 min at 4 °C to obtain post-nuclear supernatants (PNS); this step was repeated once, and the resulting supernatants were pooled. After equilibration of the LD Column (Miltenyi Biotec) with PBS containing 0.5% BSA, the combined PNS was loaded onto the column attached to a QuadroMACS Separator magnet, followed by washes with SCA buffer and PBS. Magnetically labeled organelles were eluted by removing the column from the magnetic field and flushing with 200 µL of elution buffer (5 mM Tris-HCl, pH 7.5, 1% Triton X-100, with protease and phosphatase inhibitors). All procedures were performed at 4 °C. All isolation and handling steps were performed under gentle mechanical conditions to minimize membrane disruption and preserve organelle integrity during fractionation. The isolated lysosomal and endosomal fractions were used for downstream Western blot or mass spectrometry analyses.

### Lysosome degradation activity assay in neurons

DIV14 cortical neurons were incubated with DQ™ Red BSA (10 µg/mL; Thermo Fisher Scientific; Cat# D12051) diluted in culture medium for 6 h. Following incubation, cells were briefly rinsed with HBSS and transferred to fresh, pre-warmed medium. Live-cell imaging was then conducted immediately under the same acquisition parameters as described previously. Soma-associated fluorescence intensity was quantified using ImageJ. Specifically, Corrected Total Cell Fluorescence (CTCF) was calculated by measuring raw fluorescence in individual somas and subtracting background signal from adjacent non-cellular areas. For each culture, >10 neurons per condition were analyzed. Values were averaged within each culture to generate one biological replicate, and statistical analyses were performed on these biological replicates. To rule out the possibility that observed differences in DQ-BSA signal were due to variations in uptake efficiency, total intracellular levels of DQ-BSA were also assessed from whole-cell lysates after the 6-h incubation. Immediately prior to lysis, cells were subjected to stringent surface stripping using an ice-cold acid wash (0.2 M acetic acid/0.5 M NaCl, pH 2.5), followed by extensive PBS washes to remove residual extracellular or surface-associated proteins. These measurements served as uptake controls, ensuring equivalent endocytic internalization across experimental groups.

### Immunocytochemistry

For immunostaining, neurons cultured on poly-D-lysine-coated 35-mm glass-bottom dishes were washed with PBS and fixed with 4% paraformaldehyde (Millipore Sigma; Cat# P6148) for 10 min at room temperature, washed twice with PBS, and then permeabilized for 15 min on ice with 0.1% Triton X-100 in PBS. Cells were washed three times with PBS. Primary antibody diluted in PBS containing 3% BSA was applied to the cells and incubated at 4 °C overnight. After five washes with PBS, fluorescence-conjugated secondary antibody was applied and incubated in a dark for 1 h. After incubation with appropriately diluted DAPI (Thermo Fisher Scientific; Cat# 62248) and subsequent five PBS washes, images were captured under identical confocal imaging settings as previously described. The following antibodies were used: Mouse Monoclonal anti-EEA1(BD Biosciences; Cat# 610457), Rabbit Monoclonal anti-LRRK2 (Abcam; Cat# ab133518), Rat monoclonal anti-CD107a/LAMP1 (clone 1D4B; Proteintech; Cat# 65050-1-Ig), CoraLite®594-conjugated rabbit recombinant monoclonal anti-CD107b/LAMP2 (clone 241893A7; Proteintech; Cat# CL594-84474), Mouse Monoclonal anti-NeuN (Proteintech; Cat# 66836-1-Ig), Rabbit Polyclonal anti-TFEB (Proteintech; Cat# 13372-1-AP), Rabbit monoclonal anti-Rab7 (Cell Signaling Technology; Cat# 9367), Goat anti-Rabbit IgG (H + L) Cross-Adsorbed Secondary Antibody, Alexa Fluor™ Plus 488 (Thermo Fisher Scientific; Cat# A-11008), Goat anti-Rabbit IgG (H + L) Cross-Adsorbed Secondary Antibody, Alexa Fluor™ 594 (Thermo Fisher Scientific; Cat# A-11012), Goat anti-Mouse IgG (H + L) Cross-Adsorbed Secondary Antibody, Alexa Fluor™ 488 (Thermo Fisher Scientific; Cat# A-11001), Goat anti-Mouse IgG (H + L) Cross-Adsorbed Secondary Antibody, Alexa Fluor™ 594 (Thermo Fisher Scientific; Cat# A-11005), and Goat anti-rat IgG (H + L) cross-adsorbed secondary antibody, Alexa Fluor™ 488 (Thermo Fisher Scientific; Cat# A-11006).

### Lysosomal fraction proteomic data analysis

Analysis of the label-free quantification (LFQ) proteome mass spectrometry data was performed using R. Initial quality control included filtering proteins based on a coverage percentage >2%, with the criterion of Top == “TRUE,” which indicates that the protein was identified by the top-ranking peptides in the mass spectrometry analysis. Proteins with a high proportion of missing values were excluded. Lysosome-enriched proteins were identified using the R package Limma (v3.52.4) by comparing the lysosomal fraction to the input background (PNS, post-nuclear supernatant). Proteins with an enrichment score (EnScore) > 1.1 and a *p*-value < 0.1 were defined as lysosome-enriched, with the EnScore defined as the ratio of protein abundance in the lysosomal fraction to the PNS. Differences in EnScore between PFF- and PBS-treated samples were compared, and a cutoff of 0.4 for the EnScore difference was applied to identify proteins specifically enriched in PFF-treated lysosomes or PBS-treated lysosomes. To identify differentially abundant proteins between the lysosomal and cytoplasmic fractions, the ratio of protein abundance (Lyso/Flow-through) in the lysosomal fraction to the lysosomal-depleted cytoplasmic fractions were calculated for each sample in a paired manner, and a *t*-test was applied to determine the significance of the group differences. Proteins with a Lyso/Flow-through difference between treatments greater than 1.1 and a *p*-value less than 0.1 were considered significantly different in their fractional abundance.

### Rab5 interactome proteomic analysis

The data processing platform and quality control criteria were consistent with the lysosomal proteomics analysis described above. Rab5 interactome proteins were identified using the R package Limma (v3.52.4) by comparing protein abundances in the Rab5 immunoprecipitation (IP) fraction versus the background IgG-IP fraction. Proteins with a fold-change >1.3 and a *p*-value < 0.3 were considered Rab5-associated proteins.

To investigate protein-protein interactions, the identified proteins were analyzed using the STRING database (Version12.0) (https://string-db.org) with a confidence score threshold of 0.4. K-means clustering was applied (k = 10) to group proteins and identify enriched Gene Ontology (GO) terms in the biological process (BP) category. The resulting interaction network was visualized using Cytoscape (version 3.10.2), where proteins are represented as nodes and interactions as edges. Node diameter was scaled to reflect node degree, representing the number of connected partners.

### Functional and pathway enrichment analysis

GO and KEGG pathway enrichment analyses were conducted with DAVID (v2023q4) (https://david.ncifcrf.gov), a tool that streamlines the interpretation of large gene lists using various annotation resources. Enriched biological processes and pathways were identified based on an adjusted *p*-value threshold of <0.05.

### RNA-seq analysis

Raw RNA-seq reads, generated on the Illumina NovaSeq 6000 platform in PE150 mode, were quality-checked using FastQC (v0.11.2) and trimmed with Trim Galore (v0.6.7) to remove low-quality bases and adapter sequences. Clean reads were then aligned to the mouse reference genome (GRCm38, Mus musculus) using HISAT2 (v2.2.1). Gene expression levels were quantified with StringTie (v2.2.1). Gene counts were obtained with FeatureCounts (v2.0.3) and used for differential expression analysis with DESeq2 (v1.36.0). Differentially expressed genes (DEGs) were identified using the following criteria: fold-change ≥1.2 and adjusted *p*-value < 0.05. Four biological replicates were included per condition.

### CLEAR motif scanning analysis

CLEAR (Coordinated Lysosomal Expression and Regulation) motif analysis of gene promoter regions was performed using TFEBexplorer^81^. For each gene, the output included the number of predicted binding sites, binding sequences, genomic positions, distances from the transcription start site (TSS), and Position Weight Matrix (PWM) scores. To define high-confidence predictions, a PWM score threshold of ≥0.85 was applied in motif enrichment analysis, ensuring the reliability of identified binding sites.

### ATAC-seq analysis

Primary cortical neurons were cultured in 6-well plates, and ATAC-seq libraries were constructed using the ATAC-Seq Kit (Active Motif; Cat# 53150) according to the manufacturer’s instructions. Libraries were preliminarily quantified using a Qubit 3.0 Fluorometer (Life Technologies) and submitted to the IGM Genomics Center, where library quality was assessed, including fragment size distribution analysis using the Agilent Bioanalyzer 2100 system (Agilent Technologies), followed by high-throughput sequencing. Raw ATAC-seq reads were quality-checked using FastQC and trimmed with Trim Galore (v0.6.7) to remove low-quality bases and adapter sequences. Bowtie2 (v2.5.1) software was used to align the clean reads to the reference genome (GRCm38, Mus musculus) under default mapping parameters. MACS2 (version 2.2.7.1) software was used for peak detection to obtain the open chromatin regions of the whole genome of each sample. Differential chromatin accessibility regions (Diff Peak) were identified across biological triplicates (*n* = 3 per condition) using DiffChIPL (v0.1.0) with stringent statistical criteria: Benjamini–Hochberg adjusted *p* < 0.05 and absolute fold-change ≥1.5. Genomic annotation of differential peaks was performed using the HOMER suite (v4.11), and chromatin accessibility patterns were visualized using deepTools (v3.5.1) based on normalized read-depth matrices.

To gain mechanistic insights, both known and de novo motif enrichment analyses were performed on differentially accessible regions using the findMotifsGenome.pl module in HOMER, with non-differential peaks serving as background controls. Scanning parameters included motif lengths of 8, 10, and 12 bp, with the -size parameter set to genomic region width.

To link chromatin accessibility changes with transcriptional outputs, integrative analysis was performed using BETA (v1.0.7)^82^, which infers regulatory potential by correlating differential chromatin accessibility with gene expression changes. Specifically, the Regulatory Potential Score is a composite score calculated by BETA for each gene, based on the number, strength, and proximity of differential peaks (e.g., ATAC-seq peaks) relative to the gene’s transcription start site (TSS). Higher scores indicate a stronger predicted regulatory influence (activation or repression) by nearby chromatin changes. By plotting the cumulative distribution of gene ranks, BETA evaluates whether genes in a particular category (e.g., upregulated or downregulated in RNA-seq) tend to be enriched at the top of the ranked list, which would suggest that chromatin accessibility changes near those genes likely drive transcriptional activation or repression. Statistical significance is assessed by the Kolmogorov–Smirnov test. This approach predicts direct target genes and assesses the overall directionality of regulation (activation or repression) based on chromatin dynamics.

### Statistic analysis

Significance levels were determined using GraphPad Prism 10.0 (GraphPad Software). All data were presented as mean ± SD. Statistical significance between two groups was determined using unpaired Student’s *t* test. For comparisons involving more than two conditions, one-way ANOVA followed by Tukey’s post hoc test was used. The specific statistical tests used for each figure are indicated in the figure legends. A *p* value < 0.05 was considered statistically significant (*p < 0.05, **p < 0.01, ***p < 0.001, ****p < 0.0001). No animals or data points were excluded from the analyses unless otherwise stated. All experiments were performed with at least three independent biological replicates. For imaging and electrophysiological experiments, samples were randomly assigned to experimental groups, and data acquisition was performed in a blinded manner. Data analysis was performed without blinding.

## Supplementary information

Supplementary Information

Supplementary Data 1

Supplementary Data 2

Supplementary Data 3

Supplementary Data 4

Supplementary Data 5

Supplementary Data 6

Supplementary Data 7

## Data Availability

All sequencing data generated in this study have been deposited in the NCBI Gene Expression Omnibus (GEO) under accession numbers GSE294140 (RNA-seq) and GSE294156 (ATAC-seq). Additional data supporting the findings of this study are available from the corresponding author upon reasonable request.

## References

[1] Koopman, M. B., Ferrari, L. & Rudiger, S. G. D. How do protein aggregates escape quality control in neurodegeneration? *Trends Neurosci.* **45**, 257–271 (2022). 35210101 10.1016/j.tins.2022.01.006

[2] Shahmoradian, S. H. et al. Lewy pathology in Parkinson’s disease consists of crowded organelles and lipid membranes. *Nat. Neurosci.* **22**, 1099–1109 (2019). 31235907 10.1038/s41593-019-0423-2

[3] Mahul-Mellier, A. L. et al. The process of Lewy body formation, rather than simply alpha-synuclein fibrillization, is one of the major drivers of neurodegeneration. *Proc. Natl. Acad. Sci. USA* **117**, 4971–4982 (2020). 32075919 10.1073/pnas.1913904117 PMC7060668

[4] Burre, J. et al. Alpha-synuclein promotes SNARE-complex assembly in vivo and in vitro. *Science* **329**, 1663–1667 (2010). 20798282 10.1126/science.1195227 PMC3235365

[5] Vargas, K. J. et al. Synucleins have multiple effects on presynaptic architecture. *Cell Rep.* **18**, 161–173 (2017). 28052246 10.1016/j.celrep.2016.12.023 PMC5510332

[6] Volpicelli-Daley, L. A., Luk, K. C. & Lee, V. M. Addition of exogenous alpha-synuclein preformed fibrils to primary neuronal cultures to seed recruitment of endogenous alpha-synuclein to Lewy body and Lewy neurite-like aggregates. *Nat. Protoc.* **9**, 2135–2146 (2014). 25122523 10.1038/nprot.2014.143 PMC4372899

[7] Volpicelli-Daley, L. A. et al. Exogenous alpha-synuclein fibrils induce Lewy body pathology leading to synaptic dysfunction and neuron death. *Neuron* **72**, 57–71 (2011). 21982369 10.1016/j.neuron.2011.08.033 PMC3204802

[8] Abdelmotilib, H. et al. alpha-Synuclein fibril-induced inclusion spread in rats and mice correlates with dopaminergic neurodegeneration. *Neurobiol. Dis.* **105**, 84–98 (2017). 28576704 10.1016/j.nbd.2017.05.014 PMC5701756

[9] Paumier, K. L. et al. Intrastriatal injection of pre-formed mouse alpha-synuclein fibrils into rats triggers alpha-synuclein pathology and bilateral nigrostriatal degeneration. *Neurobiol. Dis.* **82**, 185–199 (2015). 26093169 10.1016/j.nbd.2015.06.003 PMC4640952

[10] Klumperman, J. & Raposo, G. The complex ultrastructure of the endolysosomal system. *Cold Spring Harb. Perspect. Biol.* **6**, a016857 (2014). 24851870 10.1101/cshperspect.a016857 PMC4176003

[11] Ferguson, S. M. Axonal transport and maturation of lysosomes. *Curr. Opin. Neurobiol.* **51**, 45–51 (2018). 29529416 10.1016/j.conb.2018.02.020 PMC6066426

[12] Gomez, R. C., Wawro, P., Lis, P., Alessi, D. R. & Pfeffer, S. R. Membrane association but not identity is required for LRRK2 activation and phosphorylation of Rab GTPases. *J. Cell Biol.* **218**, 4157–4170 (2019). 31624137 10.1083/jcb.201902184 PMC6891090

[13] Gonzalez-Rodriguez, P., Zampese, E. & Surmeier, D. J. Selective neuronal vulnerability in Parkinson’s disease. *Prog. Brain Res.* **252**, 61–89 (2020). 32247375 10.1016/bs.pbr.2020.02.005

[14] Perera, R. M., Di Malta, C. & Ballabio, A. MiT/TFE family of transcription factors, lysosomes, and cancer. *Annu. Rev. Cancer Biol.* **3**, 203–222 (2019). 31650096 10.1146/annurev-cancerbio-030518-055835 PMC6812561

[15] Palmieri, M. et al. Characterization of the CLEAR network reveals an integrated control of cellular clearance pathways. *Hum. Mol. Genet.* **20**, 3852–3866 (2011). 21752829 10.1093/hmg/ddr306

[16] Puertollano, R., Ferguson, S. M., Brugarolas, J. & Ballabio, A. The complex relationship between TFEB transcription factor phosphorylation and subcellular localization. *EMBO J.* **37**. 10.15252/embj.201798804 (2018). 10.15252/embj.201798804 PMC5983138 29764979

[17] Muraleedharan, A. & Vanderperre, B. The endo-lysosomal system in Parkinson’s disease: expanding the horizon. *J. Mol. Biol.* **435**, 168140 (2023). 37148997 10.1016/j.jmb.2023.168140

[18] Oji, Y. et al. Variants in saposin D domain of prosaposin gene linked to Parkinson’s disease. *Brain* **143**, 1190–1205 (2020). 32201884 10.1093/brain/awaa064

[19] Robak, L. A. et al. Excessive burden of lysosomal storage disorder gene variants in Parkinson’s disease. *Brain* **140**, 3191–3203 (2017). 29140481 10.1093/brain/awx285 PMC5841393

[20] Udayar, V., Chen, Y., Sidransky, E. & Jagasia, R. Lysosomal dysfunction in neurodegeneration: emerging concepts and methods. *Trends Neurosci.* **45**, 184–199 (2022). 35034773 10.1016/j.tins.2021.12.004 PMC8854344

[21] Nixon, R. A. & Rubinsztein, D. C. Mechanisms of autophagy-lysosome dysfunction in neurodegenerative diseases. *Nat. Rev. Mol. Cell Biol.* **25**, 926–946 (2024). 39107446 10.1038/s41580-024-00757-5 PMC12239022

[22] Kuwahara, T. & Iwatsubo, T. The emerging functions of LRRK2 and Rab GTPases in the endolysosomal system. *Front. Neurosci.* **14**, 227 (2020). 32256311 10.3389/fnins.2020.00227 PMC7095371

[23] Erb, M. L. & Moore, D. J. LRRK2 and the endolysosomal system in Parkinson’s disease. *J. Parkinsons Dis.* **10**, 1271–1291 (2020). 33044192 10.3233/JPD-202138 PMC7677880

[24] Kania, E. et al. LRRK2 phosphorylation status and kinase activity regulate (macro)autophagy in a Rab8a/Rab10-dependent manner. *Cell Death Dis.* **14**, 436 (2023). 37454104 10.1038/s41419-023-05964-0 PMC10349885

[25] Pfeffer, S. R. LRRK2 phosphorylation of Rab GTPases in Parkinson’s disease. *FEBS Lett.* **597**, 811–818 (2023). 36114007 10.1002/1873-3468.14492

[26] Pfeffer, S. R. LRRK2 and Rab GTPases. *Biochem Soc. Trans.* **46**, 1707–1712 (2018). 30467121 10.1042/BST20180470

[27] Bonet-Ponce, L. et al. LRRK2 mediates tubulation and vesicle sorting from lysosomes. *Sci. Adv.* **6**, 10.1126/sciadv.abb2454 (2020). 10.1126/sciadv.abb2454 PMC7673727 33177079

[28] Kluss, J. H., Bonet-Ponce, L., Lewis, P. A. & Cookson, M. R. Directing LRRK2 to membranes of the endolysosomal pathway triggers RAB phosphorylation and JIP4 recruitment. *Neurobiol. Dis.* **170**, 105769 (2022). 35580815 10.1016/j.nbd.2022.105769 PMC9665168

[29] Zeigerer, A. et al. Rab5 is necessary for the biogenesis of the endolysosomal system in vivo. *Nature* **485**, 465–470 (2012). 22622570 10.1038/nature11133

[30] Steger, M. et al. Systematic proteomic analysis of LRRK2-mediated Rab GTPase phosphorylation establishes a connection to ciliogenesis. *Elife* **6**, 10.7554/eLife.31012 (2017). 10.7554/eLife.31012 PMC5695910 29125462

[31] Jeong, G. R. et al. Dysregulated phosphorylation of Rab GTPases by LRRK2 induces neurodegeneration. *Mol. Neurodegener.* **13**, 8 (2018). 29439717 10.1186/s13024-018-0240-1 PMC5811984

[32] Fujiwara, H. et al. alpha-Synuclein is phosphorylated in synucleinopathy lesions. *Nat. Cell Biol.* **4**, 160–164 (2002). 11813001 10.1038/ncb748

[33] Barral, D. C. et al. Current methods to analyze lysosome morphology, positioning, motility and function. *Traffic* **23**, 238–269 (2022). 35343629 10.1111/tra.12839 PMC9323414

[34] Henry, A. G. et al. Pathogenic LRRK2 mutations, through increased kinase activity, produce enlarged lysosomes with reduced degradative capacity and increase ATP13A2 expression. *Hum. Mol. Genet.* **24**, 6013–6028 (2015). 26251043 10.1093/hmg/ddv314

[35] Dou, Z. et al. Autophagy mediates degradation of nuclear lamina. *Nature* **527**, 105–109 (2015). 26524528 10.1038/nature15548 PMC4824414

[36] Shin, H. R. & Zoncu, R. The lysosome at the intersection of cellular growth and destruction. *Dev. Cell* **54**, 226–238 (2020). 32610045 10.1016/j.devcel.2020.06.010 PMC7959181

[37] Tang, T., Hasan, M. & Capelluto, D. G. S. Phafins are more than phosphoinositide-binding proteins. *Int. J. Mol. Sci.* **24**, 10.3390/ijms24098096 (2023). 10.3390/ijms24098096 PMC10178739 37175801

[38] Sardiello, M. et al. A gene network regulating lysosomal biogenesis and function. *Science* **325**, 473–477 (2009). 19556463 10.1126/science.1174447

[39] de Luzy, I. R., Lee, M. K., Mobley, W. C. & Studer, L. Lessons from inducible pluripotent stem cell models on neuronal senescence in aging and neurodegeneration. *Nat. Aging* **4**, 309–318 (2024). 38429379 10.1038/s43587-024-00586-3 PMC11824472

[40] Huang, P. et al. P2X4 forms functional ATP-activated cation channels on lysosomal membranes regulated by luminal pH. *J. Biol. Chem.* **289**, 17658–17667 (2014). 24817123 10.1074/jbc.M114.552158 PMC4067200

[41] Usmani, A., Shavarebi, F. & Hiniker, A. The cell biology of LRRK2 in Parkinson’s disease. *Mol. Cell. Biol.* **41**, 10.1128/mcb.00660-20 (2023). 10.1128/MCB.00660-20 PMC8088268 33526455

[42] Xu, L., Nagai, Y., Kajihara, Y., Ito, G. & Tomita, T. The regulation of Rab GTPases by phosphorylation. *Biomolecules* **11**, 10.3390/biom11091340 (2021). 10.3390/biom11091340 PMC8469595 34572553

[43] Anding, A. L. et al. Vps13D encodes a ubiquitin-binding protein that is required for the regulation of mitochondrial size and clearance. *Curr. Biol.* **28**, 287–295.e286 (2018). 29307555 10.1016/j.cub.2017.11.064 PMC5787036

[44] Dresselhaus, E. C. et al. ESCRT disruption provides evidence against trans-synaptic signaling via extracellular vesicles. *J. Cell Biol.* **223**, 10.1083/jcb.202405025 (2024). 10.1083/jcb.202405025 PMC11157088 38842573

[45] Vazquez-Velez, G. E. & Zoghbi, H. Y. Parkinson’s disease genetics and pathophysiology. *Annu. Rev. Neurosci.* **44**, 87–108 (2021). 34236893 10.1146/annurev-neuro-100720-034518

[46] Aoto, K. et al. ATP6V0A1 encoding the a1-subunit of the V0 domain of vacuolar H(+)-ATPases is essential for brain development in humans and mice. *Nat. Commun.* **12**, 2107 (2021). 33833240 10.1038/s41467-021-22389-5 PMC8032687

[47] Todd, T. W., Shao, W., Zhang, Y. J. & Petrucelli, L. The endolysosomal pathway and ALS/FTD. *Trends Neurosci.* **46**, 1025–1041 (2023). 37827960 10.1016/j.tins.2023.09.004 PMC10841821

[48] Small, S. A. & Petsko, G. A. Retromer in Alzheimer disease, Parkinson disease and other neurological disorders. *Nat. Rev. Neurosci.* **16**, 126–132 (2015). 25669742 10.1038/nrn3896

[49] Tekirdag, K. & Cuervo, A. M. Chaperone-mediated autophagy and endosomal microautophagy: Joint by a chaperone. *J. Biol. Chem.* **293**, 5414–5424 (2018). 29247007 10.1074/jbc.R117.818237 PMC5900761

[50] Zhu, H., Yoshimoto, T. & Yamashima, T. Heat shock protein 70.1 (Hsp70.1) affects neuronal cell fate by regulating lysosomal acid sphingomyelinase. *J. Biol. Chem.* **289**, 27432–27443 (2014). 25074941 10.1074/jbc.M114.560334 PMC4183783

[51] Welz, T., Wellbourne-Wood, J. & Kerkhoff, E. Orchestration of cell surface proteins by Rab11. *Trends Cell Biol.* **24**, 407–415 (2014). 24675420 10.1016/j.tcb.2014.02.004

[52] York, H. M. et al. Deterministic early endosomal maturations emerge from a stochastic trigger-and-convert mechanism. *Nat. Commun.* **14**, 4652 (2023). 37532690 10.1038/s41467-023-40428-1 PMC10397212

[53] Hanafusa, H. et al. LRRK1 phosphorylation of Rab7 at S72 links trafficking of EGFR-containing endosomes to its effector RILP. *J. Cell Sci*. **132**, 10.1242/jcs.228809 (2019). 10.1242/jcs.228809 31085713

[54] Malik, A. U. et al. PKC isoforms activate LRRK1 kinase by phosphorylating conserved residues (Ser1064, Ser1074 and Thr1075) within the CORB GTPase domain. *Biochem. J.* **479**, 1941–1965 (2022). 36040231 10.1042/BCJ20220308 PMC9555798

[55] Poewe, W. et al. Parkinson disease. *Nat. Rev. Dis. Prim.* **3**, 17013 (2017). 28332488 10.1038/nrdp.2017.13

[56] GBD 2016 Parkinson's Disease Collaborators Global, regional, and national burden of Parkinson’s disease, 1990-2016: a systematic analysis for the Global Burden of Disease Study 2016. *Lancet Neurol.* **17**, 939–953 (2018). 30287051 10.1016/S1474-4422(18)30295-3 PMC6191528

[57] Vidyadhara, D. J., Lee, J. E. & Chandra, S. S. Role of the endolysosomal system in Parkinson’s disease. *J. Neurochem.* **150**, 487–506 (2019). 31287913 10.1111/jnc.14820 PMC6707858

[58] Winslow, A. R. et al. alpha-Synuclein impairs macroautophagy: implications for Parkinson’s disease. *J. Cell Biol.* **190**, 1023–1037 (2010). 20855506 10.1083/jcb.201003122 PMC3101586

[59] Raj, A. et al. Dysregulation of protein degradation and alteration of secretome in alpha-synuclein-exposed astrocytes: implications for dopaminergic neuronal dysfunction. *Cell Commun. Signal* **22**, 574 (2024). 39617881 10.1186/s12964-024-01928-9 PMC11610152

[60] Lin, W. J. et al. Lysosomal targeting of phafin1 mediated by Rab7 induces autophagosome formation. *Biochem. Biophys. Res. Commun.* **417**, 35–42 (2012). 22115783 10.1016/j.bbrc.2011.11.043

[61] Matias, I. et al. Loss of lamin-B1 and defective nuclear morphology are hallmarks of astrocyte senescence in vitro and in the aging human hippocampus. *Aging Cell* **21**, e13521 (2022). 34894056 10.1111/acel.13521 PMC8761005

[62] Bedrosian, T. A. et al. Lamin B1 decline underlies age-related loss of adult hippocampal neurogenesis. *EMBO J.* **40**, e105819 (2021). 33300615 10.15252/embj.2020105819 PMC7849303

[63] Jang, H. Y., Kim, S. J., Park, K. S. & Kim, J. H. Klotho prevents transforming growth factor-beta2-induced senescent-like morphological changes in the retinal pigment epithelium. *Cell Death Dis.* **14**, 334 (2023). 37210384 10.1038/s41419-023-05851-8 PMC10199917

[64] Gire, V., Roux, P., Wynford-Thomas, D., Brondello, J. M. & Dulic, V. DNA damage checkpoint kinase Chk2 triggers replicative senescence. *EMBO J.* **23**, 2554–2563 (2004). 15192702 10.1038/sj.emboj.7600259 PMC449769

[65] Herdy, J. R. et al. Increased post-mitotic senescence in aged human neurons is a pathological feature of Alzheimer’s disease. *Cell Stem Cell* **29**, 1637–1652.e1636 (2022). 36459967 10.1016/j.stem.2022.11.010 PMC10093780

[66] Ferreira, F. J. et al. FOXM1 expression reverts aging chromatin profiles through repression of the senescence-associated pioneer factor AP-1. *Nat. Commun.* **16**, 2931 (2025). 40133272 10.1038/s41467-025-57503-4 PMC11937471

[67] De Cecco, M. et al. Genomes of replicatively senescent cells undergo global epigenetic changes leading to gene silencing and activation of transposable elements. *Aging Cell* **12**, 247–256 (2013). 23360310 10.1111/acel.12047 PMC3618682

[68] Song, Q. et al. Integrated multi-omics approach revealed cellular senescence landscape. *Nucleic Acids Res.* **50**, 10947–10963 (2022). 36243980 10.1093/nar/gkac885 PMC9638896

[69] Dixon, J. R. et al. Topological domains in mammalian genomes identified by analysis of chromatin interactions. *Nature* **485**, 376–380 (2012). 22495300 10.1038/nature11082 PMC3356448

[70] Kriukov, D. et al. Nuclear expansion and chromatin structure remodeling in mouse aging neurons. *NAR Mol. Med.* **1**, 10.1093/narmme/ugae011 (2024). 10.1093/narmme/ugae011 PMC12430011 41256807

[71] Hoffmann, A. C. et al. Extracellular aggregated alpha synuclein primarily triggers lysosomal dysfunction in neural cells prevented by trehalose. *Sci. Rep.* **9**, 544 (2019). 30679445 10.1038/s41598-018-35811-8 PMC6345801

[72] Guiney, S. J. et al. Fibrillar alpha-synuclein toxicity depends on functional lysosomes. *J. Biol. Chem.* **295**, 17497–17513 (2020). 33453994 10.1074/jbc.RA120.013428 PMC7762966

[73] Alessi, D. R. & Pfeffer, S. R. Leucine-rich repeat kinases. *Annu. Rev. Biochem.* **93**, 261–287 (2024). 38621236 10.1146/annurev-biochem-030122-051144

[74] Shrivastava, R., Pradhan, G., Ghosh, S. & Mukhopadhyay, S. Rabaptin5 acts as a key regulator for Rab7l1-mediated phagosome maturation process. *Immunology* **165**, 328–340 (2022). 34888849 10.1111/imm.13438

[75] Eguchi, T. et al. LRRK2 and its substrate Rab GTPases are sequentially targeted onto stressed lysosomes and maintain their homeostasis. *Proc. Natl. Acad. Sci. USA* **115**, E9115–E9124 (2018). 30209220 10.1073/pnas.1812196115 PMC6166828

[76] Zhu, H. et al. Rab29-dependent asymmetrical activation of leucine-rich repeat kinase 2. *Science* **382**, 1404–1411 (2023). 38127736 10.1126/science.adi9926 PMC10786121

[77] Kingwell, K. LRRK2-targeted Parkinson disease drug advances into phase III. *Nat. Rev. Drug Discov.* **22**, 3–5 (2023). 36509915 10.1038/d41573-022-00212-0

[78] Ysselstein, D. et al. LRRK2 kinase activity regulates lysosomal glucocerebrosidase in neurons derived from Parkinson’s disease patients. *Nat. Commun.* **10**, 5570 (2019). 31804465 10.1038/s41467-019-13413-w PMC6895201

[79] Fang, F. et al. Synuclein impairs trafficking and signaling of BDNF in a mouse model of Parkinson’s disease. *Sci. Rep.* **7**, 3868 (2017). 28634349 10.1038/s41598-017-04232-4 PMC5478665

[80] Roney, J. C. et al. Lipid-mediated motor-adaptor sequestration impairs axonal lysosome delivery leading to autophagic stress and dystrophy in Niemann-Pick type C. *Dev. Cell* **56**, 1452–1468.e1458 (2021). 33878344 10.1016/j.devcel.2021.03.032 PMC8137671

[81] De Cegli, R. et al. TFEBexplorer: an integrated tool to study genes regulated by the stress-responsive Transcription Factor EB. *Autophagy Rep.* **1**, 295–305 (2022). 40396031 10.1080/27694127.2022.2097822 PMC11864625

[82] Wang, S. et al. Target analysis by integration of transcriptome and ChIP-seq data with BETA. *Nat. Protoc.* **8**, 2502–2515 (2013). 24263090 10.1038/nprot.2013.150 PMC4135175

