# Supplementary material for: Preformed fibrils of α-synuclein rapidly activate LRRK2 on early endosomes, driving Rab5 phosphorylation and disrupting endolysosomal and synaptic function
Source: NPJ Parkinsons Dis. 2026 May 12;12:176. doi: 10.1038/s41531-026-01382-z (PMC13392030; doi:10.1038/s41531-026-01382-z)
Supplement: Supplementary file 1 — Supplementary Information [file 41531_2026_1382_MOESM1_ESM.pdf]

Supplementary Figures and Legends

Supplementary Fig. 1

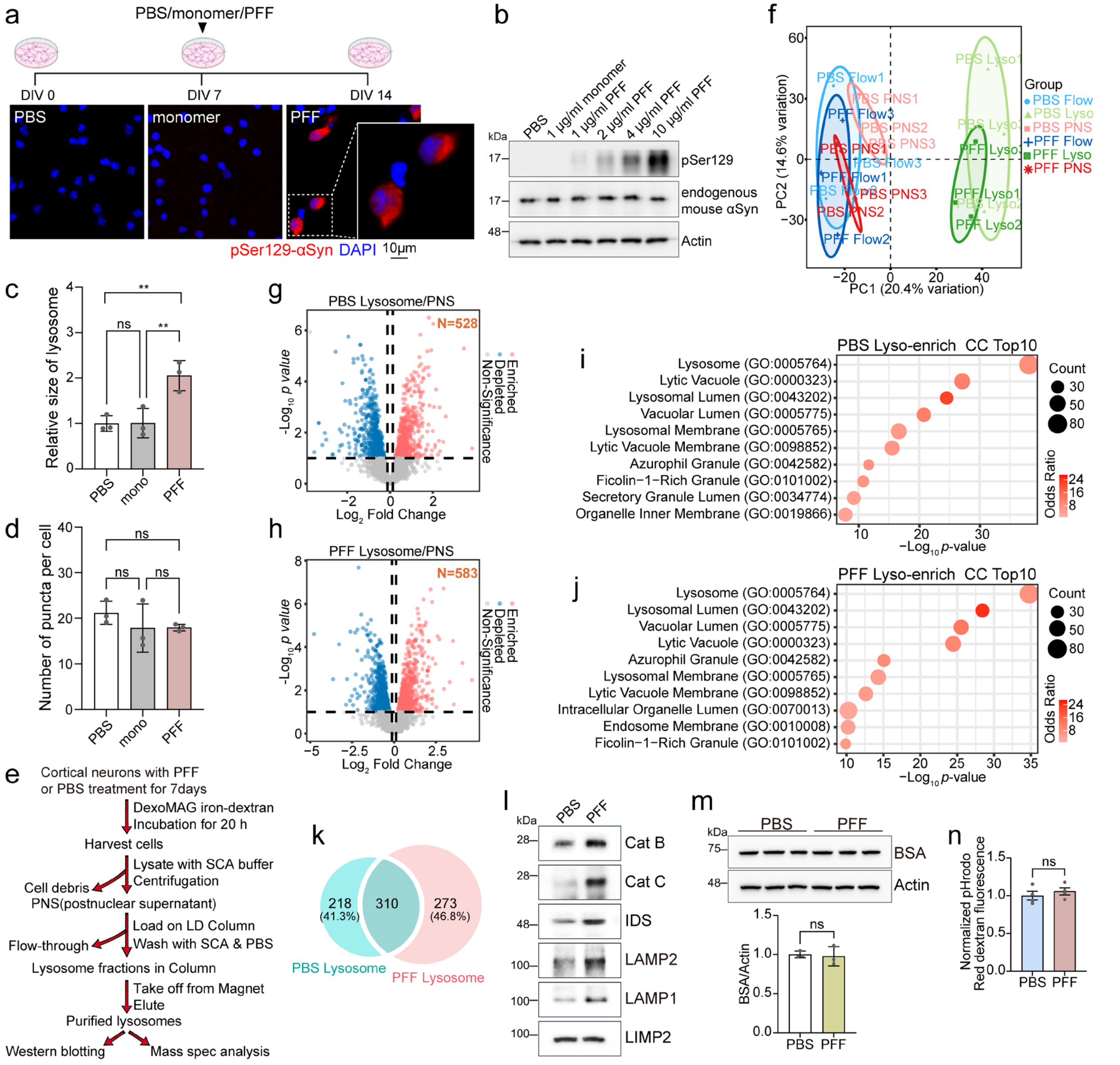

Supplementary Fig. 1: Characterization of lysosomal alterations and proteomic profiling in neurons treated with PBS or PFF.

(**a**) Immunofluorescent imaging using the pSer129-αSyn phosphorylation-specific antibody in mouse primary neurons 7 days post-treatment by PBS, 1 μg/mL αSyn monomer and 1 μg/mL PFF. Scale bar, 10 μm. Representative image from n = 3 independent experiments.

(**b**) Western blotting of pSer129-αSyn and endogenous mouse αSyn in neurons after incubation with PBS, αSyn monomer and different concentrations of PFFs for 7 days. Actin was included as a loading control. Representative image from n = 3 independent experiments.

(**c**) Quantification of lysosomal size in primary mouse cortical neurons treated with PBS, αSyn monomer, or PFF. Lysosomal size was determined based on LysoTracker fluorescence intensity. >30 cells were analyzed per condition. Data are presented as mean ± SD from n = 3 independent experiments. Statistical significance was determined by one-

way ANOVA followed by Tukey's post hoc test; ns, not significant; $**p < 0.01$.
(**d**) Quantification of lysosome number per cell in neurons treated with PBS, αSyn monomer, or PFF. >30 cells were analyzed per condition. Data are presented as mean ± SD ($n = 3$); one-way ANOVA followed by Tukey's post hoc test; ns, not significant.
(**e**) The fractionation experiment scheme for isolating lysosomes with dextran-coated magnetite beads from mouse primary neurons treated with PFF or PBS for 7 days.
(**f**)The PCA plot shows the distribution of individual samples based on proteomics data from different cellular fractions, including flow-through (Flow), lysosomal (Lyso), and post-nuclear supernatant (PNS) fractions in different colors. Analyses based on proteomic data from $n = 3$ independent experiments.
(**g**, **h**) Volcano plots display the differential enrichment of proteins in lysosomal fractions compared to post-nuclear supernatant (PNS) in PBS-treated (**g**) and PFF-treated (**h**) primary neurons. Significantly enriched proteins are shown in orange, depleted proteins in blue, and nonsignificant proteins in gray. Analyses based on proteomic data from $n = 3$ independent experiments.
(**i**, **j**) Gene Ontology (GO) Cellular Component (CC) analysis of lysosome-enriched proteins identified by mass spectrometry under PBS (**i**) and PFF (**j**) treatment conditions, showing the top 10 enriched terms for each group. Analyses based on proteomic data from $n = 3$ independent experiments.
(**k**) The Venn diagram shows the overlap of proteins enriched in lysosome fractions between PBS-treated and PFF-treated primary neurons. Numbers indicate the quantitation of proteins in each category. Analyses based on proteomic data from $n = 3$ independent experiments.
(**l**) Western blotting of the expression level of lysosome residential proteins in isolated lysosomal fractions. LIMP2 was used as an internal reference for lysosomal protein abundance. Representative image from $n = 3$ independent experiments.
(**m**) Assessment of total cellular levels of DQ™ Red BSA in PBS- and PFF-treated neurons following 6-hour incubation. Data are presented as mean ± SD ($n = 3$); unpaired Student's *t*-test; ns, not significant.
(**n**) Measurement of relative lysosomal acidity in neurons treated with PBS or PFF for 7 days, assessed by fluorescence intensity of the pH-sensitive endocytic probe pHrodo Red dextran (Thermo Fisher Scientific). Data are presented as mean ± SD ($n = 3$); unpaired Student's *t*-test; ns, not significant.

Supplementary Fig. 2

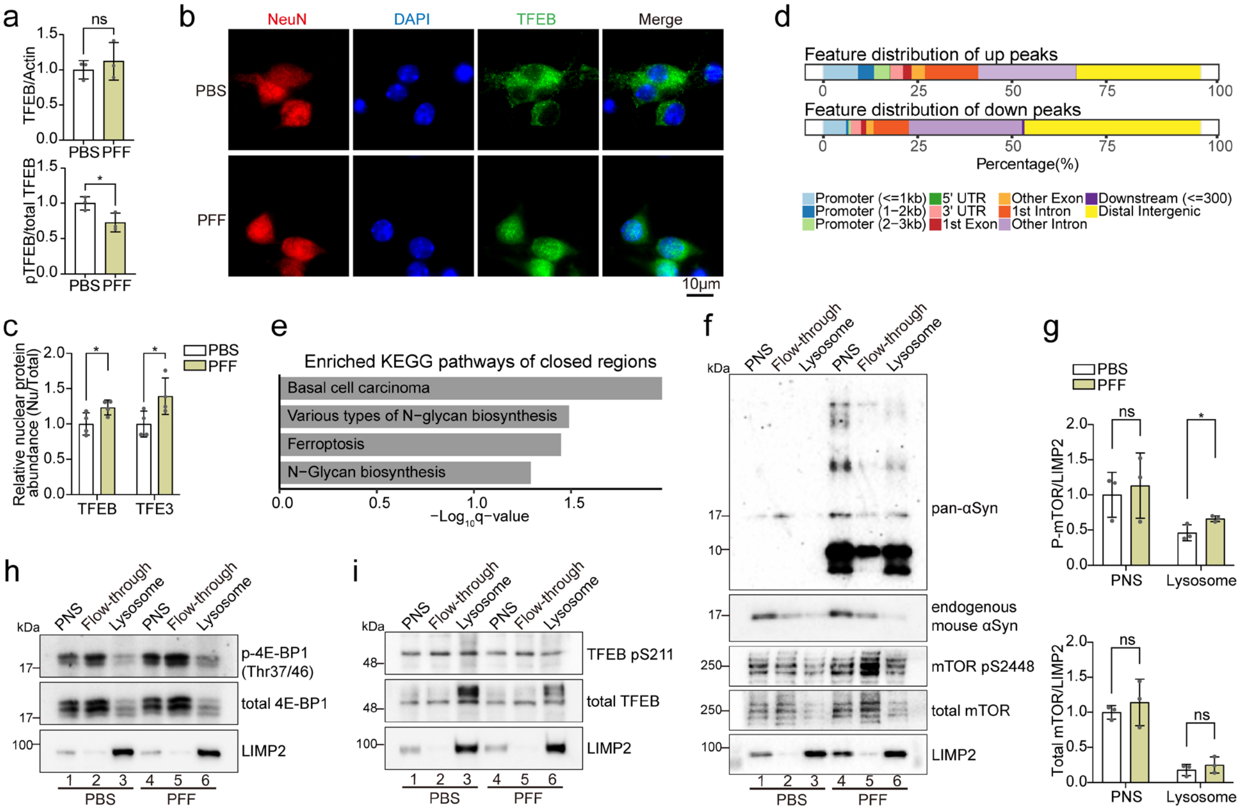

Supplementary Fig. 2: PFF-induced changes in TFEB/TFE3 signaling, chromatin accessibility, and mTOR activity.

(**a**) Quantitative analysis of total TFEB (top) and phosphorylated TFEB (bottom, Ser211) protein levels. Data are presented as mean ± SD from $n = 3$ independent experiments. Statistical significance was determined by unpaired Student's *t*-test; ns, not significant; *$p < 0.05$.

(b) Immunofluorescence of PBS- or PFF-treated cortical neurons (7 days) stained for NeuN (red), TFEB (green), and nuclei (DAPI, blue). Merged images display TFEB and DAPI channels. Scale bar, 10 μm. Representative image from $n = 3$ independent experiments.

(**c**) Nuclear abundance of TFEB and TFE3 based on biochemical nuclear-cytoplasmic fractionation, quantified as the ratio of nuclear to total protein levels in PBS- and PFF-treated neurons. Data are presented as mean ± SD ($n = 4$); unpaired Student's *t*-test; *$p < 0.05$.

(**d**) Distribution of genomic features for regions with significantly increased (up peaks) and decreased (down peaks) ATAC-seq signal. The bars show the percentage of differential peaks in various genomic features, including promoters, UTRs, exons, introns, and intergenic regions. Analyses based on ATAC-seq data from $n = 3$ independent experiments.

(**e**) KEGG pathway enrichment analysis of genes corresponding to regions with significantly decreased ATAC-seq peaks. Analyses based on ATAC-seq data from $n = 3$ independent experiments.

(**f**) Western blot analysis of the distribution of pan-αSyn, endogenous αSyn, phospho-mTOR (Ser2448) and total mTOR in post-nuclear supernatant (PNS), flow-through, and lysosome fraction. LIMP2 as the marker for lysosome isolation. Representative image from $n = 3$ independent experiments.

(**g**) Quantification of phospho-mTOR (top) and total mTOR (bottom) normalized to LIMP2 in PNS and lysosomal fractions from PBS- or PFF-treated neurons. Data are presented as mean ± SD ($n = 3$); unpaired Student's *t* test; ns, not significant; *$p < 0.05$.

(**h, i**) Western blot analysis of phosphorylated 4E-BP1 (Thr37/46) and total 4E-BP1 (**h**), and phosphorylated TFEB (Ser211) and total TFEB (**i**) in post-nuclear supernatant (PNS), flow-through, and lysosome fractions from PBS- and PFF-treated neurons. LIMP2 served as a marker for lysosome isolation. Representative images from $n = 3$ independent experiments.

Supplementary Fig. 3

a

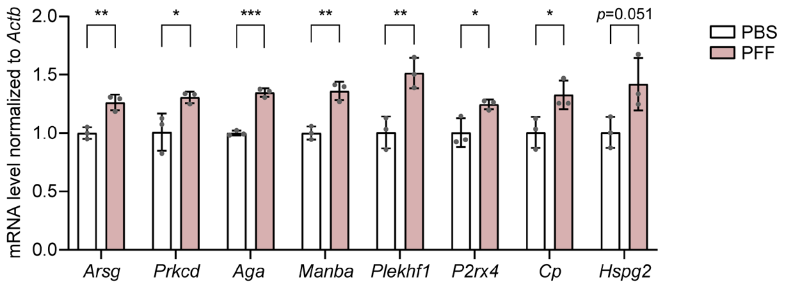

b

Genesymbol	Number	Score	Sequence	Chromsome	Start	End	Strand	TSS Position
Aga	2	0.91	GTCTCGCGAG	chr8	53511655	53511664	+	-46
		0.95	GTCACCTGCC	chr8	53512190	53512199	+	489
Arsg	1	0.91	GGCACGCGCC	chr11	109473376	109473385	+	3
Cp	1	0.95	GTCATGTGCC	chr3	19956399	19956408	+	-654
Hspg2	1	0.91	GTCAGGGGCC	chr4	137469114	137469123	+	312
Manba	1	0.91	CTCAGCTGAC	chr3	135485576	135485585	+	-34
P2rx4	1	0.93	GTCAGGGGAC	chr5	122707212	122707221	+	-331
Plekhf1	2	0.91	GGCAGGTGAG	chr7	38228531	38228540	-	-545
		0.93	GGCACCTGAG	chr7	38228300	38228309	-	-314
Prkcd	5	0.90	ATCATCTGCC	chr14	30626980	30626989	-	-780
		0.95	GGCATGTGAC	chr14	30610762	30610771	-	-460
		0.95	GGCATGTGAC	chr14	30610740	30610749	-	-438
		0.90	CTCACGTGGG	chr14	30626307	30626316	-	-107
		0.91	GTCACAGGAG	chr14	30609889	30609898	-	413

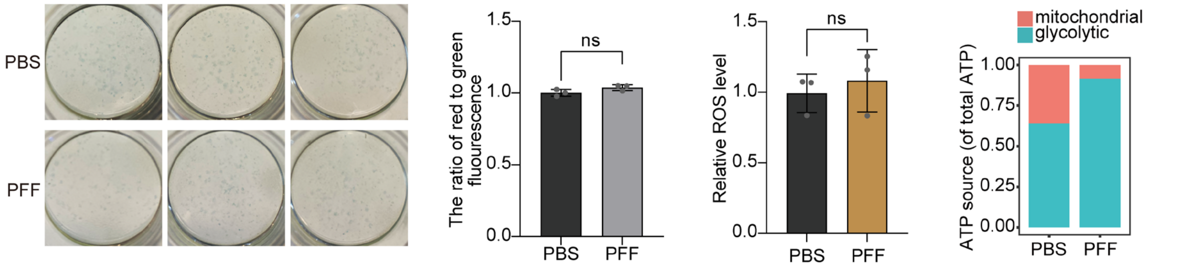

Supplementary Fig. 3: Analysis of senescence markers and cellular changes in PFF-treated mouse cortical neurons.

(**a**) RT-qPCR validation of differentially expressed lysosomal genes from RNA-seq analysis. Relative mRNA levels are normalized to *Actb*. Data are presented as mean ± SD from n = 3 independent experiments. Statistical significance was determined by unpaired Student's t-test; *$p < 0.05$, **$p < 0.01$, ***$p < 0.001$.

(**b**)The table shows predicted TFEB binding sites in the promoter-proximal regions of lysosome-related genes, identified using the TFEBexplorer tool. For each gene, the table lists the number of predicted sites, binding sequence, genomic position, distance from the transcription start site (TSS), and Position Weight Matrix (PWM) score. In motif enrichment analysis, a PWM score ≥ 0.85 is generally considered a reliable threshold for identifying high-confidence binding sites.

(**c**) Senescence-associated β-galactosidase staining in cortical neurons treated with PFF or PBS. Representative image from n = 3 independent experiments.

(**d**) Mitochondrial membrane potential in PBS- and PFF-treated neurons were assessed by JC-1(5 μM). Fluorescence intensities were normalized to the PBS group and expressed as relative values. Data are presented as mean ± SD (n = 3); unpaired Student's t-test; ns, not significant.

(**e**) Intracellular ROS levels in PBS- and PFF-treated neurons were assessed by DFCH-A (10 μM). Fluorescence intensities were normalized to the PBS group and expressed as relative values. Data are presented as mean ± SD (n = 3); unpaired Student's t-test; ns, not significant.

(**f**) ATP levels were measured with or without oligomycin to estimate glycolytic and mitochondrial contributions to total ATP in neurons treated with PBS or PFF, using the Glycolysis/OXPHOS Assay Kit (Dojindo Laboratories). Quantification showed a significant shift in ATP source contribution following PFF treatment (n = 3; one-way ANOVA followed by Tukey's post hoc test; p = 0.0054).

Supplementary Fig. 4

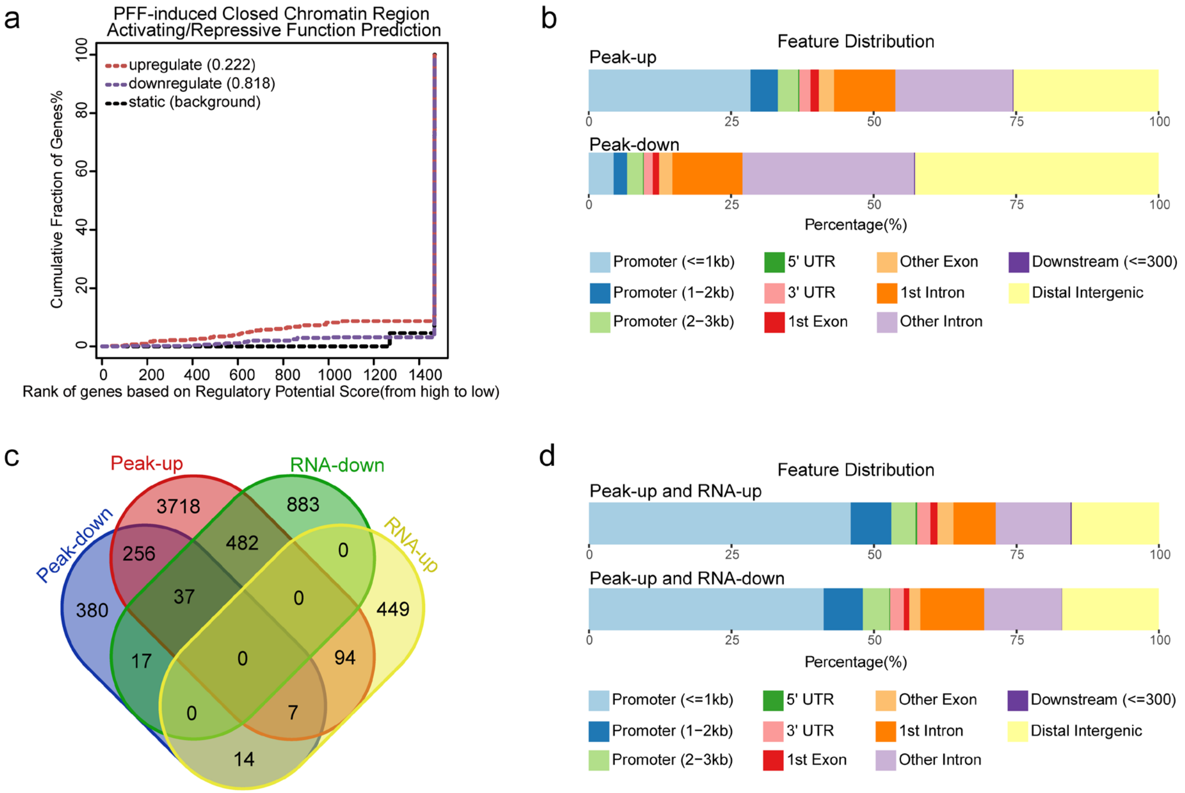

Supplementary Fig. 4: Overlap and genomic feature distribution of differential ATAC-seq peaks and DEGs.
(**a**) Correlation between PFF-induced closed chromatin accessibility and gene expression. The x-axis ranks genes by their Regulatory Potential Score (from highest to lowest), reflecting regulatory strength in closed chromatin regions. The y-axis shows the cumulative fraction of genes in each category. Red and purple curves represent upregulated and downregulated genes, respectively; the black curve represents static genes with no significant expression changes. Statistical significance was assessed by comparing up- and downregulated genes to static genes. Analyses were based on ATAC-seq data from n = 3 and RNA-seq data from n = 4 independent biological experiments.
(**b**)Genomic feature distribution of regions with increased (Peak-up) and decreased (Peak-down) chromatin accessibility. Analyses based on ATAC-seq data from n = 3 independent experiments.
(**c**) Venn diagram illustrates the overlap between RNA-seq-identified differentially expressed genes (DEGs) and genes linked to differentially accessible chromatin regions identified by ATAC-seq. Analyses were based on ATAC-seq data from n = 3 and RNA-seq data from n = 4 independent biological experiments.
(**d**) Genomic feature distribution of opened peaks associated with either upregulated or downregulated DEGs. Analyses were based on ATAC-seq data from n = 3 and RNA-seq data from n = 4 independent biological experiments.

Supplementary Fig. 5

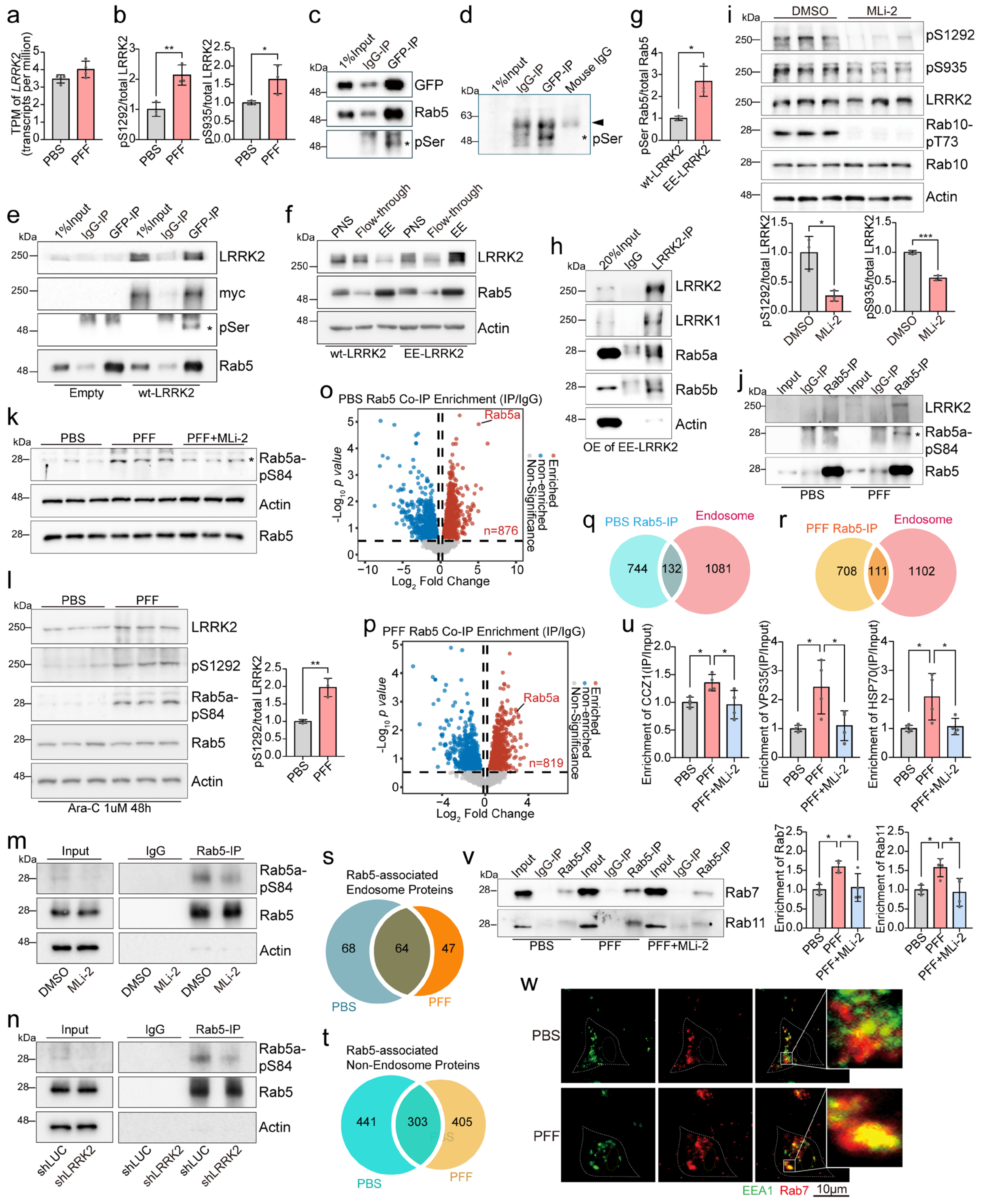

Supplementary Fig. 5: Validation of Rab5 phosphorylation and remodeling of the Rab5 interactome in neurons and cell lines.
(**a**) Bar plot showing LRRK2 expression level (Transcripts Per Million, TPM) in neurons treated with PBS or PFF. Analyses based on RNA-seq data from n = 4 independent experiments.

(**b**) Quantification of phosphorylated LRRK2 at Ser1292 (pS1292; left) and Ser935 (pS935; right), each normalized to total LRRK2, in cortical neurons treated with PBS or PFF, corresponding to Fig. **5c**. Data are presented as mean ± SD (n = 3); unpaired Student's t-test; *p < 0.05, **p < 0.01.

(**c**, **d**) Immunoprecipitation (IP) of GFP-tagged Rab5 in mouse neuroblastoma (N2a) cells. Western blot analysis was performed to detect GFP, Rab5, and phosphoserine (pSer). In (**d**), an IgG control was included in the fourth lane, and gels were run in parallel to separate IgG heavy chains from target bands. Phosphoserine-positive bands are marked with * and the IgG heavy chain is indicated by an arrowhead. Representative image from n = 3 independent experiments.

(**e**) N2a cells transfected with GFP-Rab5 were co-transfected with either myc-tagged LRRK2 or an empty control vector were subjected to GFP IP. Western blots probed for phosphoserine (pSer), Rab5, LRRK2 and the myc tag. Phosphoserine-positive bands are marked with *. Representative image from n = 3 independent experiments.

(**f**) N2a cells transfected with GFP-Rab5 were co-transfected with either myc-tagged wild-type LRRK2 or early endosome-targeted LRRK2 (EE-LRRK2) and purified early endosomes with dextran-coated magnetite beads. Western blotting was performed to assess the distribution of LRRK2, Rab5, and Actin across fractions. Representative image from n = 3 independent experiments.

(**g**) Quantification of phosphorylated Rab5 relative to total Rab5 in GFP-Rab5 IP fractions from N2a cells expressing wild-type LRRK2 or EE-targeted LRRK2, corresponding to Fig. **5g**. Data are presented as mean ± SD (n = 3); unpaired Student's t-test; *p < 0.05.

(**h**) Co-immunoprecipitation of LRRK2 from N2a cells overexpressing early endosome-targeted LRRK2. Western blot analysis was performed to detect LRRK2, LRRK1, Rab5a, Rab5b, and Actin in the input, IgG control, and LRRK2 IP fractions. Representative image from n = 3 independent experiments.

(**i**) Western blot analysis of primary cortical neurons treated with either DMSO or 600 nM MLi-2 for 5 days. Total LRRK2, phosphorylated LRRK2 at serine 1292 (pS1292), phosphorylated LRRK2 (pS935), total Rab10, and phosphorylated Rab10 (pT73) are shown, with Actin as a loading control. Quantification of pS1292/total LRRK2 (left) and pS935/total LRRK2 (right) are shown below. Data are presented as mean ± SD (n = 3); unpaired Student's t-test; *p < 0.05, ***p < 0.001.

(**j**) Primary neurons treated with PFF, or control PBS were immunoprecipitated using a Rab5-specific antibody or IgG. The levels of phosphorylated Rab5a at Ser84 (Rab5a pS84), and LRRK2 in the immunoprecipitants were evaluated by western blot. Phosphorylated Rab5 band are highlighted with *. Representative image from n = 3 independent experiments.

(**k**) Western blot analysis of phosphorylated Rab5a pS84 in cortical neurons treated with PBS, PFF, or PFF + MLi-2. Actin served as a loading control. Phosphorylated Rab5 band are highlighted with *. Representative image from n = 3 independent experiments.

(**l**) Western blot analysis of total LRRK2, phosphorylated LRRK2 at serine 1292 (pS1292), phosphorylated Rab5a at Ser84 (Rab5a pS84), and total Rab5 in cortical neuron cultures treated with PBS or PFF under Ara-C (1 μM, 48 h) conditions to suppress proliferating non-neuronal cells. Actin served as a loading control. Quantification of pS1292 relative to total LRRK2 is shown on the right. Data are presented as mean ± SD (n = 3); unpaired Student's t-test; **p < 0.01.

(**m, n**) Cortical neurons treated with DMSO or the LRRK2 inhibitor MLi-2 (**m**) or infected with lentivirus expressing control shRNA (shLUC) or LRRK2-targeting shRNA (shLRRK2) (**n**). Rab5 was immunoprecipitated from cortical neuron lysates prior to immunoblotting for phosphorylated Rab5a at Ser84 (Rab5a pS84). Input, IgG control, and Rab5-IP fractions are shown. Total Rab5 and Actin are included as controls. Representative image from n = 3 independent experiments.

(**o**, **p**) Volcano plots display the relative enrichment (IP/IgG) of proteins co-immunoprecipitated with Rab5 under PBS (**o**) and PFF (**p**) treatment conditions. Red points represent proteins enriched in co-immunoprecipitation (Co-IP), blue points are non-enriched proteins, and gray points indicate non-significant changes. The number of enriched proteins is indicated. Rab5a is highlighted. Analyses based on proteomic data from n = 4 independent experiments.

(**q**, **r**) Overlap between Rab5-IP-identified proteins and known endosomal proteins under PBS (**q**) and PFF (**r**) conditions.

(**s**) Venn diagram showing the overlap of associated endosomal proteins captured by Rab5 Co-IP in PBS and PFF conditions.

(**t**) Venn diagram showing the overlap of Rab5-associated non-canonical endosomal proteins between PBS and PFF conditions.

(**u**) Quantification of enrichment of CCZ1, VPS35 and HSP70 in Rab5 co-IP under indicated conditions. Protein levels in co-IP fraction were normalized to input. Data are presented as mean ± SD (n = 4); one-way ANOVA followed by Tukey's post hoc test; *p < 0.05.

(**v**) Rab5 co-immunoprecipitation was performed under PBS, PFF, and PFF plus MLi-2 conditions to assess the association of Rab5 with Rab7 and Rab11. Representative immunoblots (left) show proteins co-immunoprecipitated with Rab5. Quantification of Rab7 and Rab11 enrichment (right) was normalized to input. Data are presented as mean ± SD (n = 4); one-way ANOVA followed by Tukey's post hoc test; *p < 0.05.

(**w**) Immunofluorescence of cortical neurons treated with PBS or PFF for 7 days stained for early endosome marker EEA1 (green) and late endosome/lysosome marker Rab7 (red). Dashed outlines indicate neuronal soma. Scale bar, 10 μm. Representative image from n = 3 independent experiments.

Supplementary Fig. 6

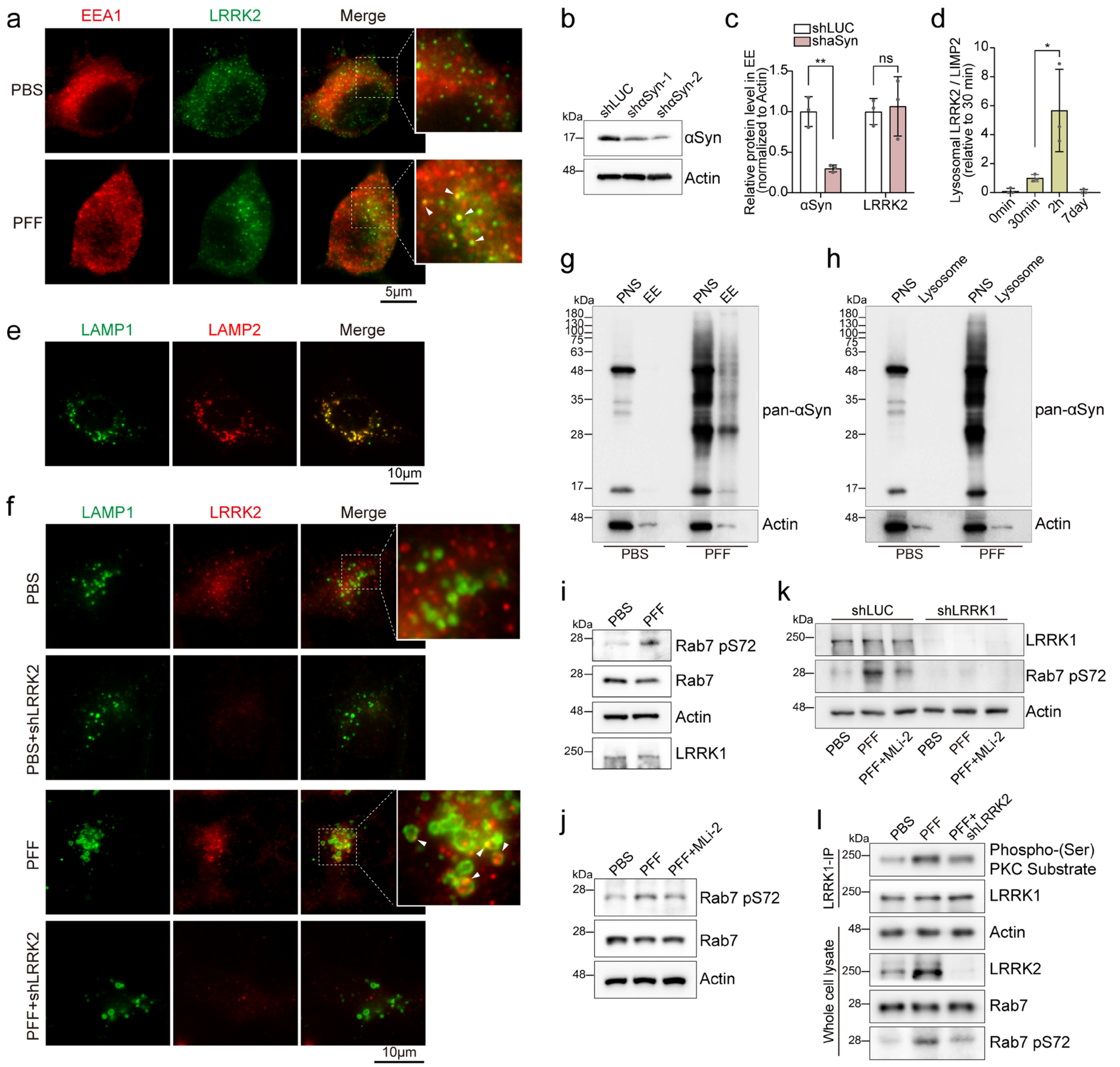

Supplementary Fig. 6: LRRK2 mediates PFF-induced phosphorylation of Rab7 in primary neurons.

(**a**) Immunofluorescence of cortical neurons treated with PBS or PFF for 2 h stained for the early endosome marker EEA1 (red) and LRRK2 (green). Merged images are shown on the right. Arrowheads indicate representative puncta. Scale bar, 5 µm. Representative image from *n* = 3 independent experiments.

(**b**) Primary cortical neurons were infected with lentivirus expressing one of two shRNAs targeting mouse α-synuclein (shaSyn-1 and shaSyn-2), or a control shRNA targeting luciferase (shLUC). Cells were harvested 3 days post-infection for western blot analysis. Actin was used as a loading control. Representative image from *n* = 3 independent experiments.

(**c**) Quantification of endogenous αSyn and LRRK2 relative to Actin in the EE fraction shown in Fig. **6b**. Data are presented as mean ± SD (*n* = 3); unpaired Student's *t*-test; ***p* < 0.01; ns, not significant.

(**d**) Quantification of lysosomal LRRK2 normalized to LIMP2 in the lysosome fraction shown in Fig. **6c**, expressed relative to the 30 min time point. Statistical comparison was performed between the 30 min and 2 h groups using an unpaired Student's *t*-test. Data are presented as mean ± SD (*n* = 3); **p* < 0.05.

(**e**) Immunofluorescence of cortical neurons stained for the lysosomal markers LAMP1 (green) and LAMP2 (red) to assess overlap of lysosomal labeling. Merged images are shown on the right. Scale bar, 10 µm. Representative image

from *n* = 3 independent experiments.

(**f**) Immunofluorescence of cortical neurons treated with PBS or PFF for 2 h, with or without shLRRK2, stained for the lysosomal marker LAMP1 (green) and LRRK2 (red). Merged images are shown in the third column, with enlarged views of the boxed regions shown on the right for PBS and PFF conditions. Arrowheads indicate representative puncta showing partial overlap of LRRK2 and LAMP1 signals under the PFF condition. Scale bar, 10 μm. Representative image from *n* = 3 independent experiments.

(**g**) Western blot analysis of pan-α-synuclein (αSyn) in post-nuclear supernatant (PNS) and early endosome (EE)-enriched fractions from primary neurons treated with PBS or PFF for 30 minutes. Actin was used as a loading control. Representative image from *n* = 3 independent experiments.

(**h**) Western blot analysis of pan-α-synuclein (αSyn) in post-nuclear supernatant (PNS) and lysosome-enriched fractions from primary neurons treated with PBS or PFF for 30 minutes. Actin was used as a loading control. Representative image from *n* = 3 independent experiments.

(**i**) Primary neurons were treated with PFF or control PBS, and total cell lysates were analyzed by western blot to assess the levels of phosphorylated Rab7 (Ser72) using a phospho-specific antibody. Total Rab7 and LRRK1 were also evaluated. Actin served as a loading control. Representative image from *n* = 3 independent experiments.

(**j**) Primary neurons were treated with PBS, PFF, or PFF in combination with the LRRK2 inhibitor MLi-2. Western blot analysis of total cell lysates was performed to assess the levels of phosphorylated Rab7 (Ser72), total Rab7, and Actin. Representative image from *n* = 3 independent experiments.

(**k**) Primary cortical neurons were infected with lentivirus expressing control shRNA (shLUC) or LRRK1-targeting shRNA (shLRRK1) and treated with PBS, PFF, or PFF in combination with the LRRK2 inhibitor MLi-2. Western blots analysis of total LRRK1, phosphorylated Rab7 at Ser72 (Rab7 pS72), and Actin as a loading control. Representative image from *n* = 3 independent experiments.

(**l**) Cortical neurons were treated with PBS, PFF, or PFF in combination with LRRK2 knockdown. LRRK1 was immunoprecipitated, followed by immunoblotting with a phospho-(Ser) PKC substrate antibody and total LRRK1 (Top panels: LRRK1-IP); Western blot analysis of total LRRK2, total Rab7, and Rab7 pS72 in whole-cell lysates (bottom panels), with Actin as a loading control. Representative image from *n* = 3 independent experiments.

Supplementary Fig. 7

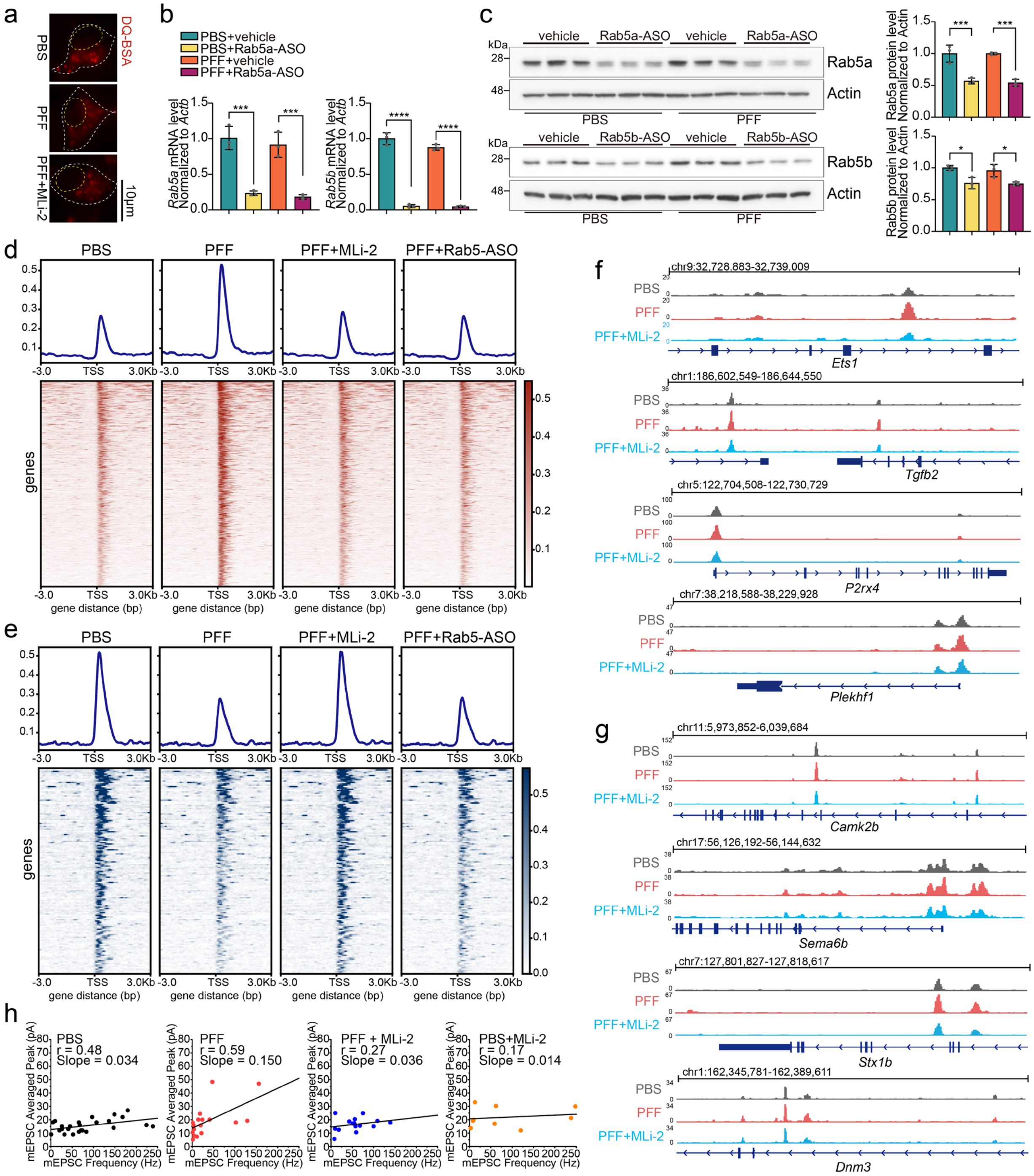

Supplementary Fig. 7: Rescue of PFF-induced changes by LRRK2 inhibition and Rab5 ASO.

(**a**) Representative live-cell imaging of DQ™ Red BSA fluorescence in mouse primary cortical neurons treated with PBS, PFF, or PFF plus MLi-2 to assess lysosomal proteolytic activity. Dashed lines outline cell boundaries. Scale bar, 10 µm. Representative image from $n = 3$ independent experiments.

(**b**) Mouse cortical neurons were treated with PBS or PFF, with or without antisense oligonucleotides (ASOs) targeting Rab5a or Rab5b. The mRNA levels of Rab5a (left) and Rab5b (right) were measured by RT-qPCR, using *Actb* as the internal control. Data are presented as mean ± SD from $n = 3$ independent experiments. Statistical significance was determined by one-way ANOVA followed by Tukey's post hoc test; $^{***}p < 0.001$, $^{****}p < 0.0001$.

(**c**) Primary mouse cortical neurons were treated with PBS or PFF, with or without Rab5a- or Rab5b-targeting antisense oligonucleotides (ASOs). Protein levels of Rab5a (top) and Rab5b (bottom) were assessed by western blot, with Actin used as a loading control. Quantification of Rab5a and Rab5b protein levels normalized to Actin is shown on the right. Data are presented as mean ± SD (n = 3); one-way ANOVA followed by Tukey's post hoc test; *$p < 0.05$, ***$p < 0.001$.
(**d**, **e**) Heatmaps showing regions with significantly increased (**d**) and decreased (**e**) ATAC-seq peak signals in PFF-treated neurons compared to PBS, with effects of MLi-2 or Rab5 ASO treatment. The heatmaps display chromatin accessibility centered around transcription start sites (TSS), extending 3 kb upstream and downstream. Analyses based on ATAC-seq data from n = 3 independent experiments.
(**f**, **g**) ATAC-seq peak diagrams illustrating changes in open chromatin regions at differentially expressed genes (DEGs), showing increased accessibility at upregulated genes (**f**) and at downregulated genes (**g**) in PFF-treated neurons compared to PBS. The tracks from top to bottom represent PBS, PFF, and PFF in combination with MLi-2 treatments. Genomic coordinates are shown at the top, and the gene structure is represented by line-box diagrams at the bottom. Analyses based on ATAC-seq data from n = 3 independent experiments.
(**h**) Correlation analysis between mEPSC frequencies and averaged mEPSC amplitudes of the primary cortical neurons treated with PBS, PFF, PFF in combination with MLi-2 or PBS in combination with MLi-2. Each dot represents an individual neuron. Pearson's correlation coefficient (*r*) and linear regression slope are indicated for each condition. Data represents n = 4 independent recordings per condition.

Supplementary Fig. 8

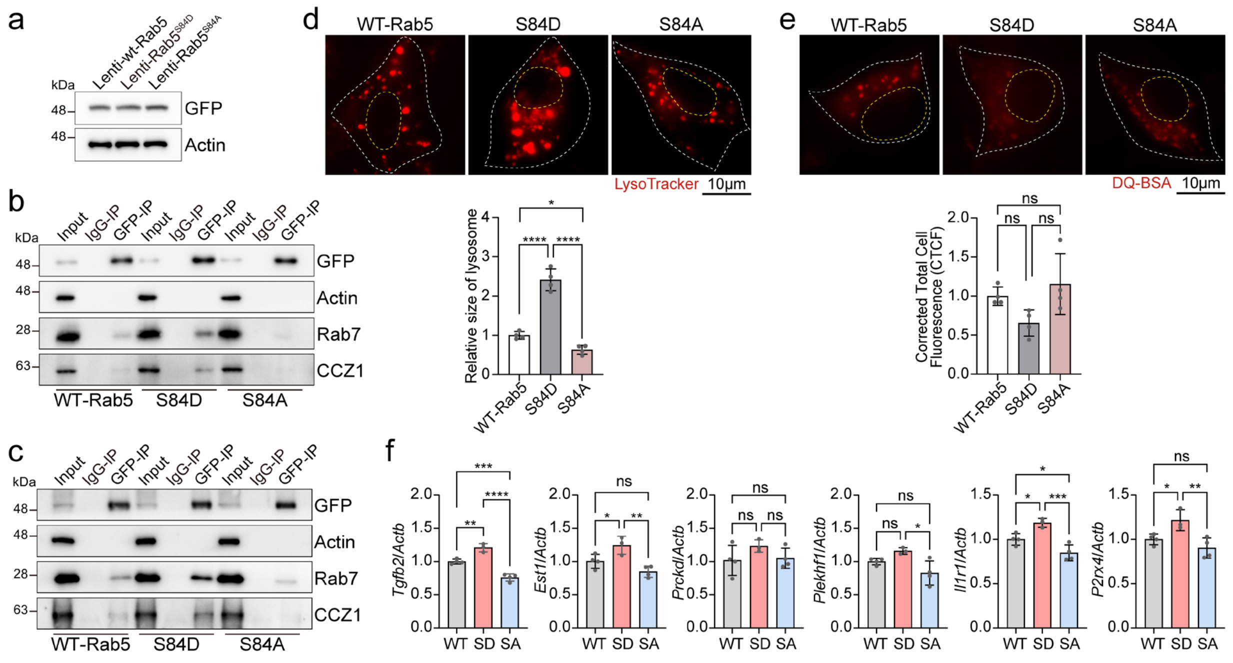

Supplementary Fig. 8: Functional analysis of Rab5a Ser84 phosphorylation using phospho-mimetic and phospho-dead mutants in neurons.

(**a**) Western blot analysis confirming expression of lentivirally delivered GFP-tagged human WT-Rab5, phospho-mimetic Rab5-S84D, and phospho-dead Rab5-S84A in primary cortical neurons, with Actin as a loading control. Representative image from n = 3 independent experiments.

(**b**) Co-immunoprecipitation of GFP-Rab5 from PBS-treated neuronal cultures followed by immunoblotting for Rab7 and CCZ1, with GFP confirming immunoprecipitation efficiency and Actin serving as a loading control. Representative image from n = 3 independent experiments.

(**c**)Co-immunoprecipitation of GFP-Rab5 from PFF-treated neuronal cultures (2h exposure) followed by immunoblotting for Rab7 and CCZ1, with GFP confirming immunoprecipitation efficiency and Actin serving as a loading control. Representative image from n = 3 independent experiments.

(**d**) Representative live-cell imaging of lysosome by LysoTracker Red DND-99 uptake in 2h PFF-treated neurons expressing WT-Rab5, Rab5-S84D, or Rab5-S84A. Lysosomal size was determined based on LysoTracker fluorescence intensity. Scale bar, 10 µm. Data are presented as mean ± SD from n = 4 independent experiments. Statistical significance was determined by one-way ANOVA followed by Tukey's post hoc test; *$p < 0.05$, ****$p < 0.0001$.

(**e**) Representative live-cell imaging of DQ™ Red BSA fluorescence in 2h PFF-treated neurons expressing WT-Rab5, Rab5-S84D, or Rab5-S84A. Quantitative analysis of corrected total cell fluorescence (CTCF) from DQ-BSA imaging. Scale bar, 10 µm. Data are presented as mean ± SD (n = 4); one-way ANOVA followed by Tukey's post hoc test; ns, not significant.

(**f**) RT-qPCR analysis in neurons expressing WT-Rab5(WT), Rab5-S84D(SD), or Rab5-S84A(SA) after 2h PFF treatment, focusing on representative genes involved in lysosomal and senescence pathways that exhibit PFF-induced chromatin opening and transcriptional upregulation, with chromatin accessibility changes reversible by MLi-2 treatment. Data are presented as mean ± SD (n = 4 for WT and S84A; n = 3 for S84D); one-way ANOVA followed by Tukey's post hoc test; ns, not significant; *$p < 0.05$, **$p < 0.01$, ***$p < 0.001$, ****$p < 0.0001$.

Supplementary Tables

Supplementary Data 1: Lysosome-enriched proteomic signatures and GO cellular component enrichment in PBS- and PFF-treated neurons.
Supplementary Data 1 includes four worksheets. Worksheets 1 and 3 list lysosome-enriched proteins identified in PBS- and PFF-treated neurons, respectively. Worksheets 2 and 4 present GO cellular component enrichment analyses of these lysosome-enriched proteins under PBS and PFF conditions. These data support the analyses presented in Fig. S1g-j.

Supplementary Data 2: Differentially enriched lysosomal proteins in PFF compared with PBS-treated neurons.
Supplementary Data 2 includes three worksheets. Worksheet 1 lists proteins with significantly altered lysosomal enrichment based on ΔEnScore in PFF- versus PBS-treated neurons. Worksheet 2 presents GO cellular component enrichment analyses of proteins with increased lysosomal enrichment under PFF treatment. Worksheet 3 provides a list of lysosomal and late endosomal proteins among those with increased enrichment in PFF-treated neurons. These data support the analyses presented in Fig. 1c.

Supplementary Data 3: Altered lysosomal versus flow-through protein distribution in PFF compared with PBS-treated neurons.
Supplementary Data 3 includes one worksheet summarizing changes in Lyso/Flow-through protein distribution upon PFF treatment. These data support the analyses presented in Fig. 1f.

Supplementary Data 4: Differential chromatin accessibility regions in PFF compared with PBS-treated neurons.
Supplementary Data 4 includes one worksheet listing differentially accessible chromatin regions identified by ATAC-seq after 7 days of PFF versus PBS treatment. These data support the analyses presented in Fig. 2c.

Supplementary Data 5: RNA-seq-based differential gene expression in PFF versus PBS-treated neurons.
Supplementary Data 5 includes three worksheets. Worksheet 1 lists differentially expressed genes identified by RNA-seq in PFF- versus PBS-treated neurons. Worksheet 2 presents TFEB-binding motifs identified in the promoter regions of upregulated genes. Worksheet 3 provides primer sequences used for RT-qPCR validation. These data support the analyses presented in Fig. 3a-f and Fig. S3a-b.

Supplementary Data 6: Overlap between differential chromatin accessibility and differentially expressed genes in PFF versus PBS-treated neurons.
Supplementary Data 6 includes one worksheet listing representative genes showing concordant or discordant changes between chromatin accessibility (ATAC-seq) and gene expression (RNA-seq), indicating whether each gene is associated with up- or down-peaks and up- or down-regulated transcripts. These data support the analyses presented in Fig. 4b-d and Fig. S4c-d.

Supplementary Data 7: Mass spectrometry-based identification of Rab5-associated proteins in PBS- and PFF-treated neurons.
Supplementary Data 7 includes three worksheets. Worksheet 1 lists Rab5-associated proteins identified by co-immunoprecipitation and mass spectrometry in PBS-treated neurons. Worksheet 2 lists Rab5-associated proteins identified under PFF treatment using the same criteria. Worksheet 3 provides endosomal proteins detected in Rab5 co-immunoprecipitates under PBS and PFF conditions, indicating condition-specific and shared interactors. These data support the analyses presented in Fig. 5j and Fig. S5o-t.

Uncropped blot

Fig. 1

Fig. 1i

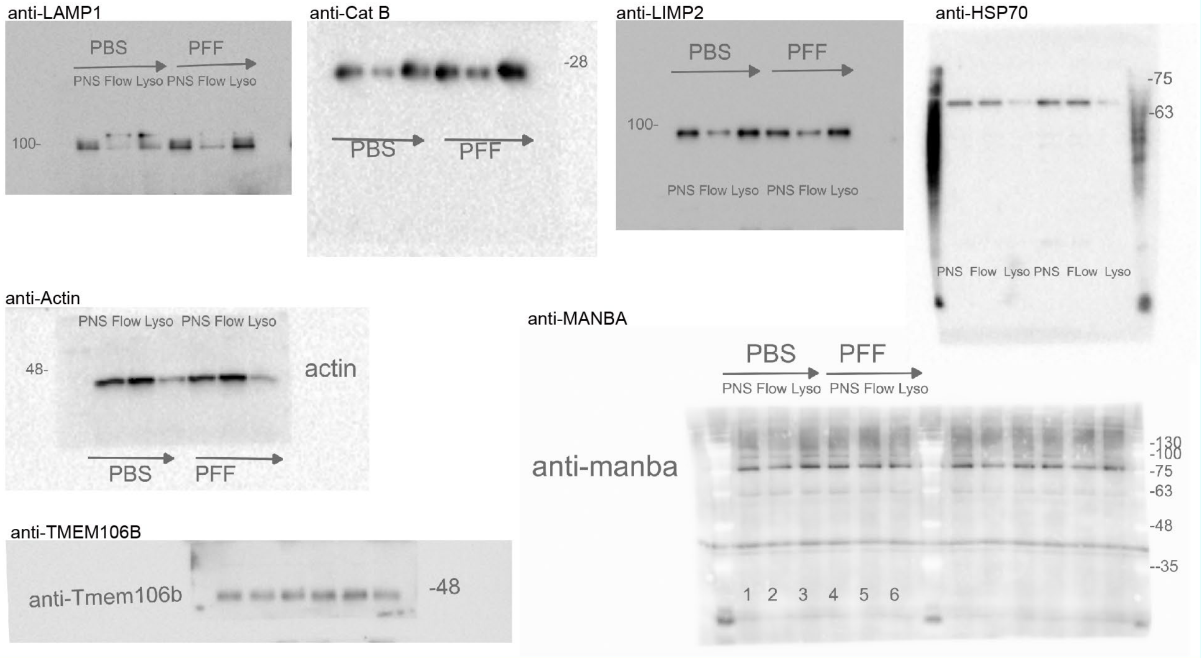

Fig. 2

Fig. 2a

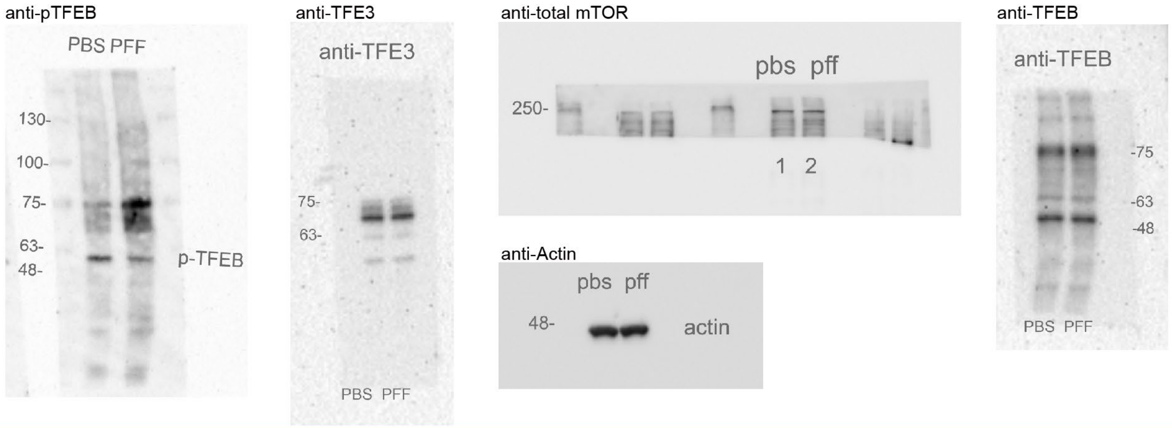

Fig. 2b

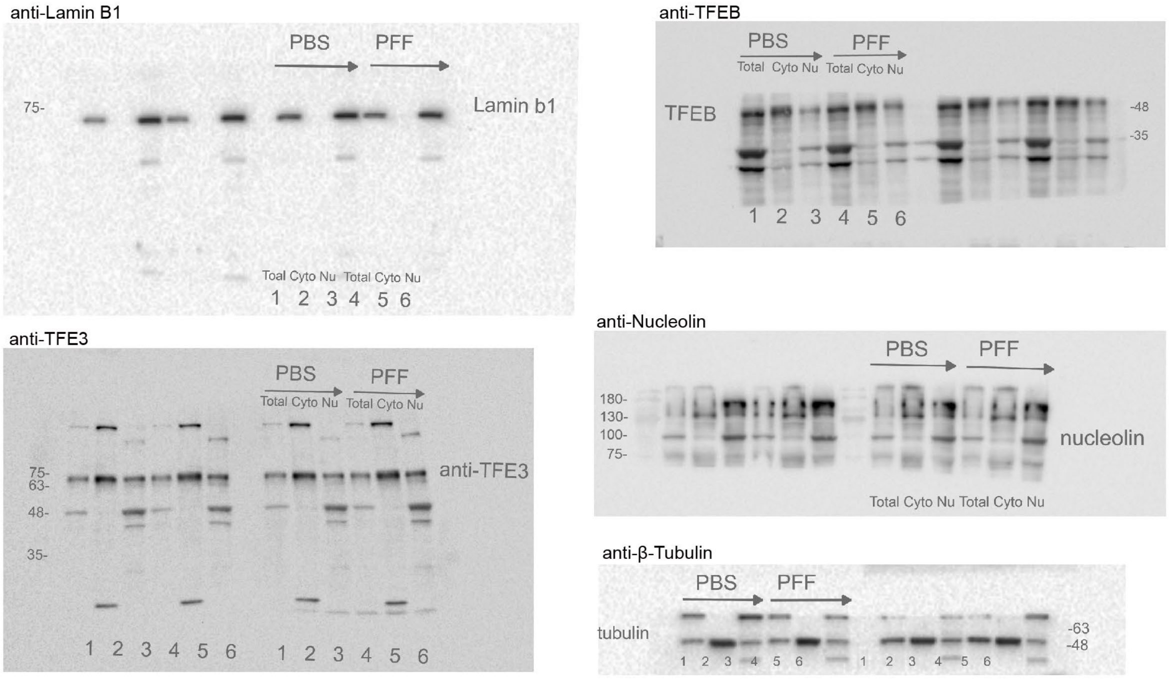

Fig. 3

Fig. 3g

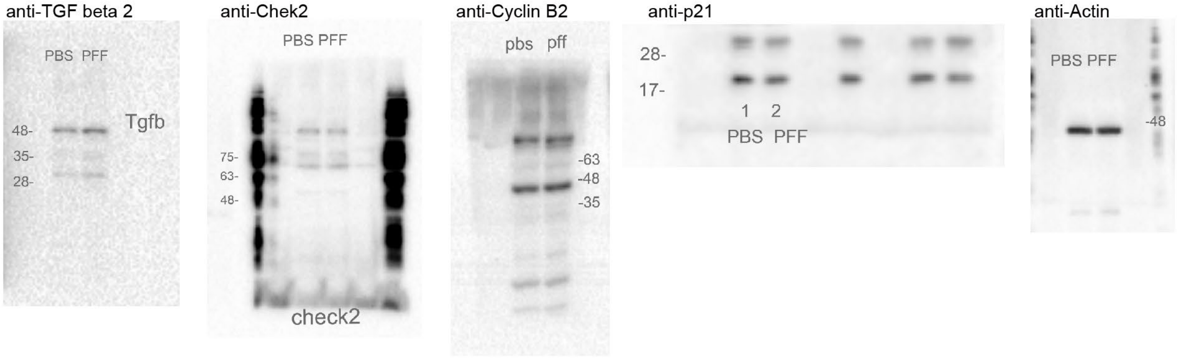

Fig. 5

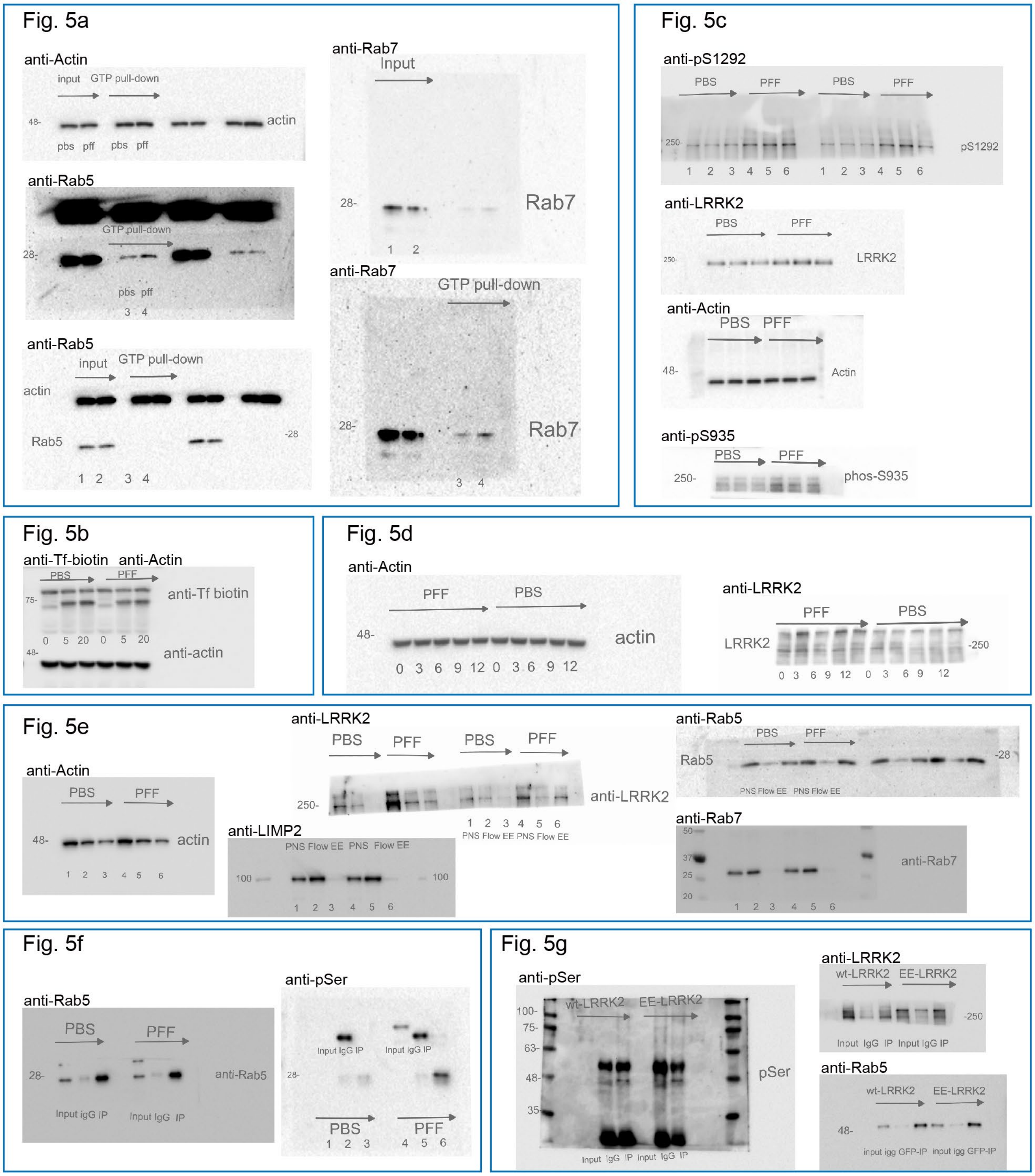
Fig. 5a
anti-Actin
input GTP pull-down
48-
actin
pbs pff pbs pff
anti-Rab5
GTP pull-down
28-
pbs pff
3 4
anti-Rab5
input GTP pull-down
actin
Rab5
-28
1 2 3 4
anti-Rab7
Input
28-
Rab7
1 2
anti-Rab7
GTP pull-down
28-
Rab7
3 4
Fig. 5c
anti-pS1292
PBS PFF PBS PFF
250-
pS1292
1 2 3 4 5 6 1 2 3 4 5 6
anti-LRRK2
PBS PFF
250-
LRRK2
anti-Actin
PBS PFF
48-
Actin
anti-pS935
PBS PFF
250-
phos-S935
Fig. 5b
anti-Tf-biotin anti-Actin
PBS PFF
75-
anti-Tf biotin
0 5 20 0 5 20
48-
anti-actin
Fig. 5d
anti-Actin
PFF PBS
48-
actin
0 3 6 9 12 0 3 6 9 12
anti-LRRK2
PFF PBS
LRRK2
-250
0 3 6 9 12 0 3 6 9 12
Fig. 5e
anti-Actin
PBS PFF
48-
actin
1 2 3 4 5 6
anti-LRRK2
PBS PFF PBS PFF
250-
anti-LRRK2
1 2 3 4 5 6
PNS Flow EE PNS Flow EE
anti-LIMP2
PNS Flow EE PNS Flow EE
100
100
1 2 3 4 5 6
anti-Rab5
PBS PFF
Rab5
-28
PNS Flow EE PNS Flow EE
anti-Rab7
50
37
25
20
anti-Rab7
1 2 3 4 5 6
Fig. 5f
anti-Rab5
PBS PFF
28-
anti-Rab5
Input igG IP Input IgG IP
anti-pSer
Input IgG IP Input IgG IP
28-
PBS PFF
1 2 3 4 5 6
Fig. 5g
anti-pSer
wt-LRRK2 EE-LRRK2
100-
75-
63-
48-
35-
pSer
Input IgG IP Input IgG IP
anti-LRRK2
wt-LRRK2 EE-LRRK2
-250
Input IgG IP Input IgG IP
anti-Rab5
wt-LRRK2 EE-LRRK2
48-
input igg GFP-IP input igg GFP-IP

Fig. 5h

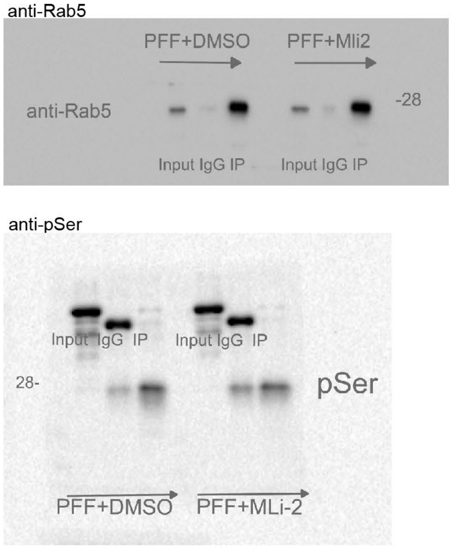

Fig. 5i

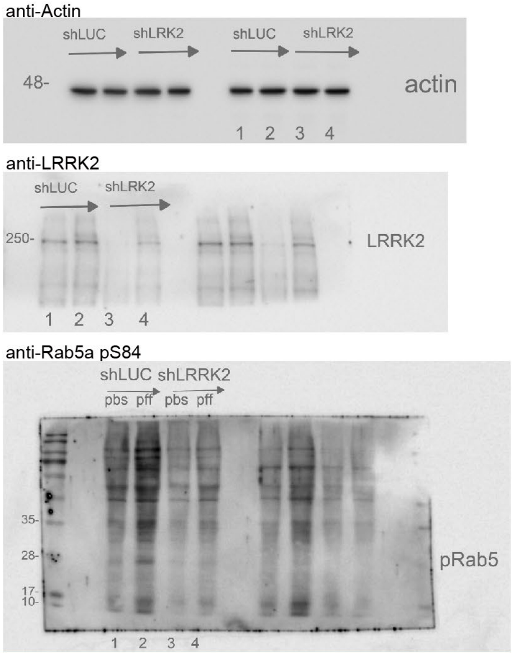

Fig. 5k

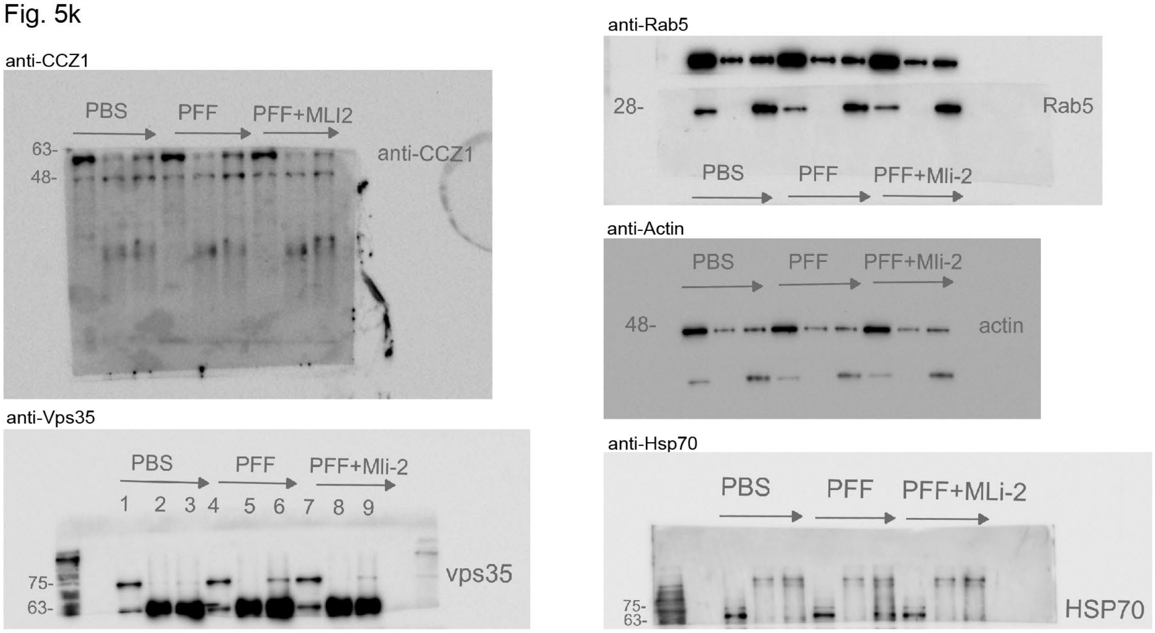

Fig. 6

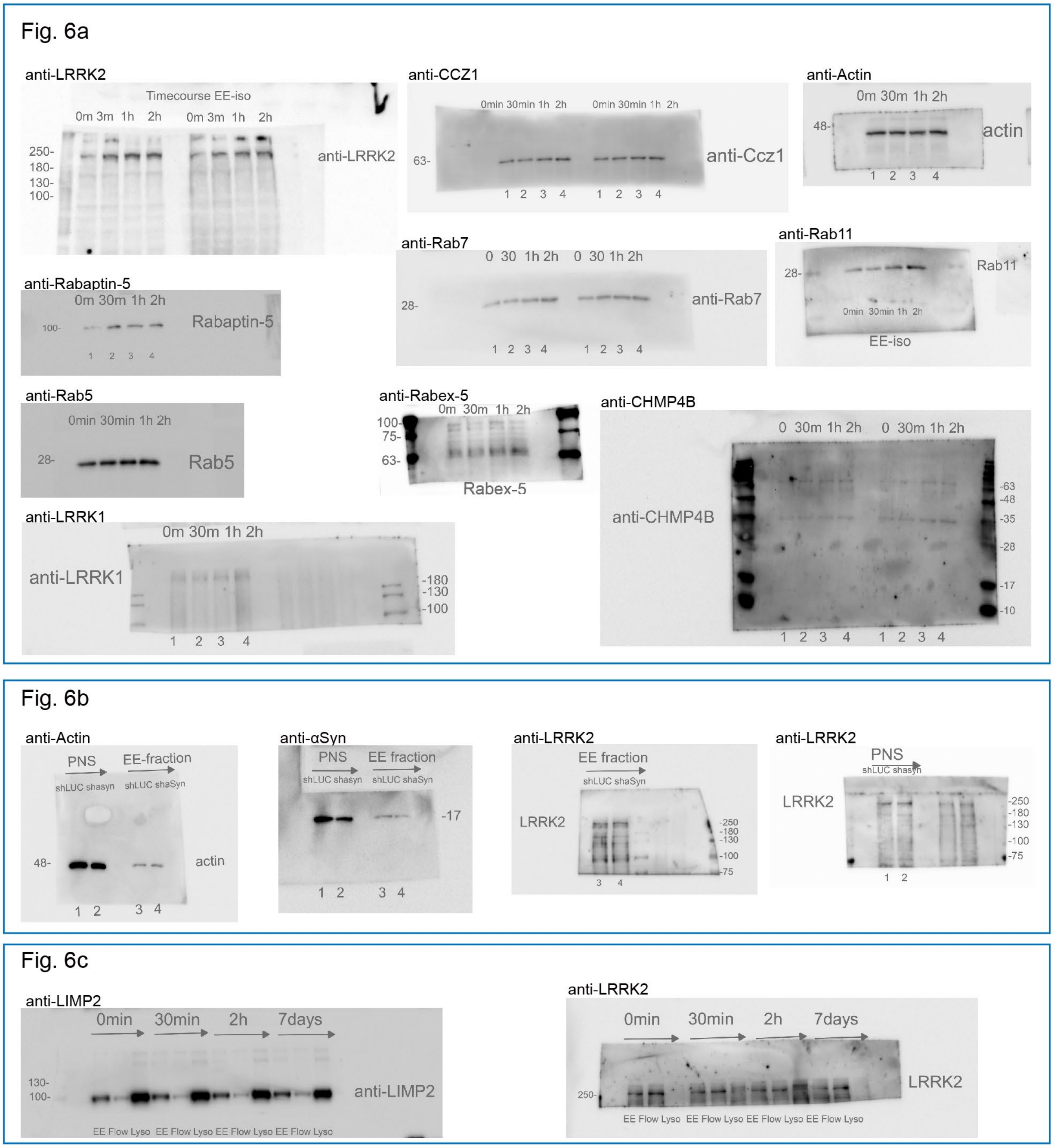
Fig. 6a
anti-LRRK2
Timecourse EE-iso
0m 3m 1h 2h 0m 3m 1h 2h
250-
180-
130-
100-
anti-LRRK2
anti-CCZ1
0min 30min 1h 2h 0min 30min 1h 2h
63-
anti-Ccz1
1 2 3 4 1 2 3 4
anti-Actin
0m 30m 1h 2h
48-
actin
1 2 3 4
anti-Rabaptin-5
0m 30m 1h 2h
100-
Rabaptin-5
1 2 3 4
anti-Rab7
0 30 1h 2h 0 30 1h 2h
28-
anti-Rab7
1 2 3 4 1 2 3 4
anti-Rab11
28-
Rab11
0min 30min 1h 2h
EE-iso
anti-Rab5
0min 30min 1h 2h
28-
Rab5
anti-Rabex-5
0m 30m 1h 2h
100-
75-
63-
Rabex-5
anti-CHMP4B
0 30m 1h 2h 0 30m 1h 2h
anti-CHMP4B
-63
-48
-35
-28
-17
-10
1 2 3 4 1 2 3 4
anti-LRRK1
0m 30m 1h 2h
anti-LRRK1
-180
-130
-100
1 2 3 4
Fig. 6b
anti-Actin
PNS
EE-fraction
shLUC shasyn shLUC shaSyn
48-
actin
1 2 3 4
anti-αSyn
PNS
EE fraction
shLUC shasyn shLUC shaSyn
-17
1 2 3 4
anti-LRRK2
EE fraction
shLUC shaSyn
LRRK2
-250
-180
-130
-100
-75
3 4
anti-LRRK2
PNS
LRRK2
-250
-180
-130
-100
-75
1 2
Fig. 6c
anti-LIMP2
0min 30min 2h 7days
130-
100-
anti-LIMP2
EE Flow Lyso EE Flow Lyso EE Flow Lyso EE Flow Lyso
anti-LRRK2
0min 30min 2h 7days
250-
LRRK2
EE Flow Lyso EE Flow Lyso EE Flow Lyso EE Flow Lyso

Fig. 6d

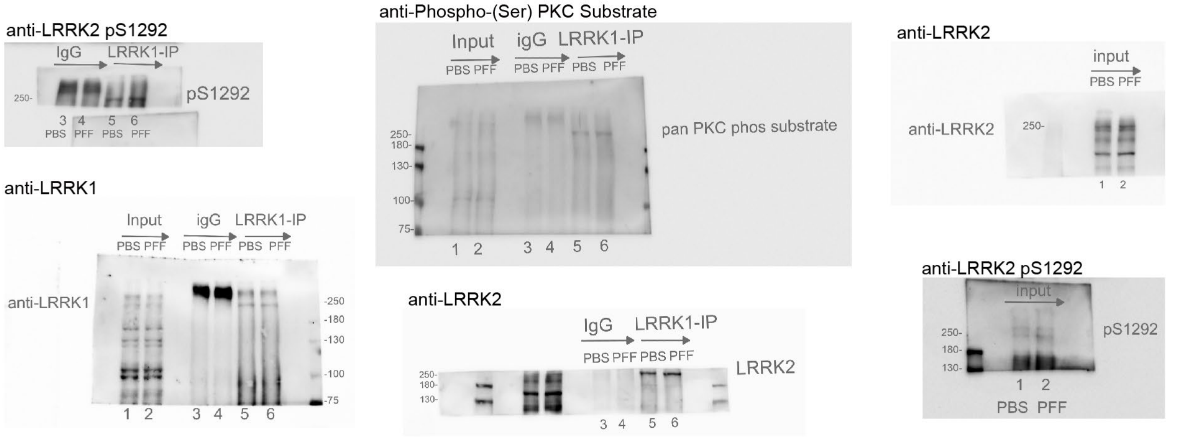

Fig. 6e

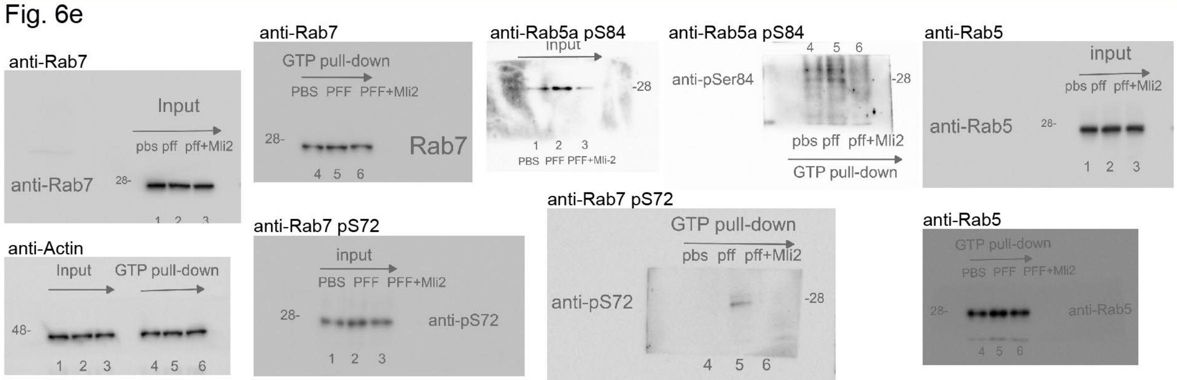

Fig. 7

Fig. 7a

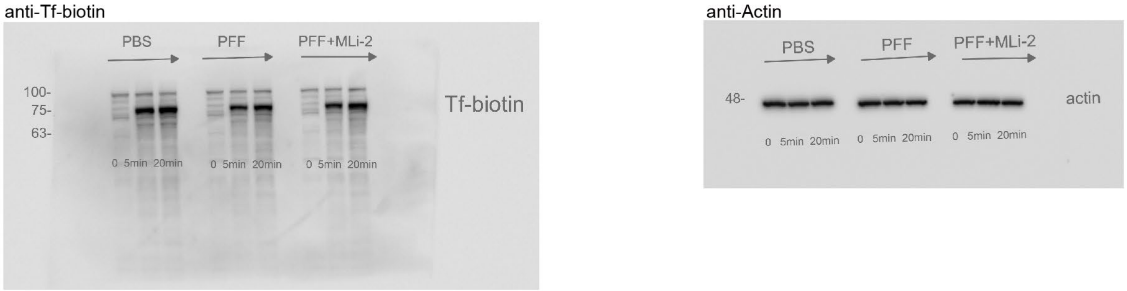

Supplementary Fig. 1

Fig. S1b

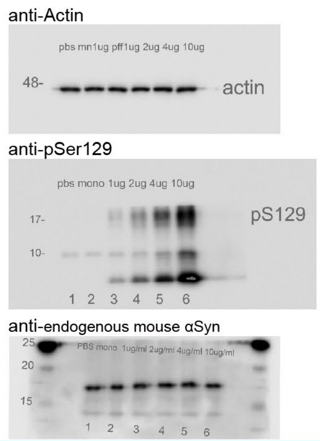

Fig. S1l

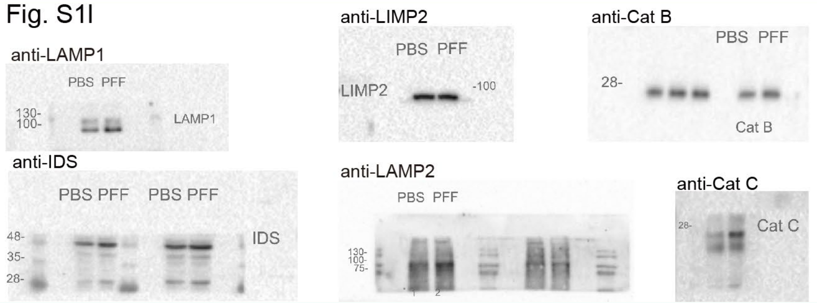

Fig. S1m

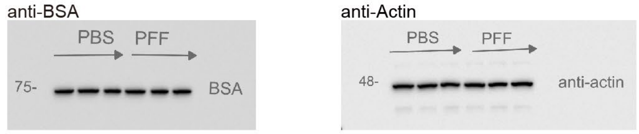

Supplementary Fig. 2

Fig. S2f

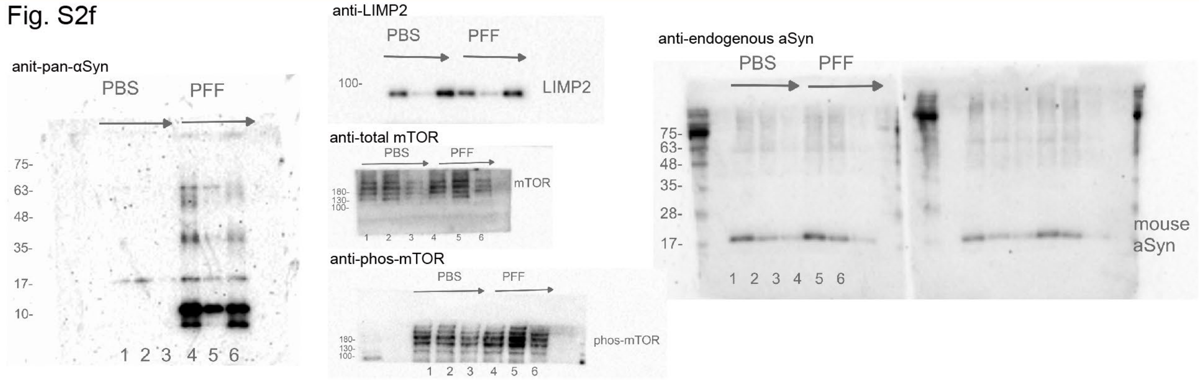

Fig. S2h

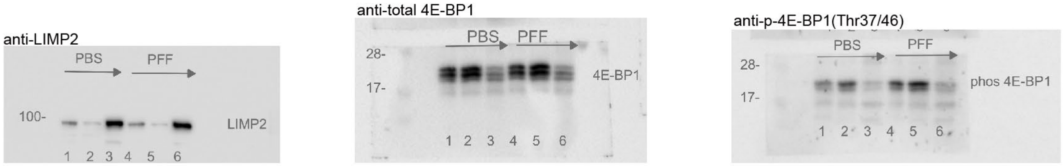

Fig. S2i

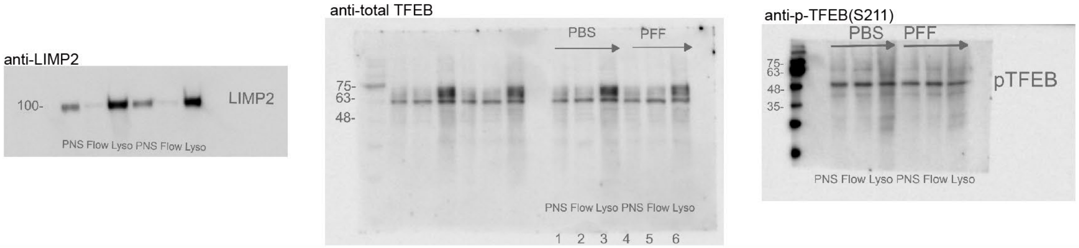

Supplementary Fig. 5

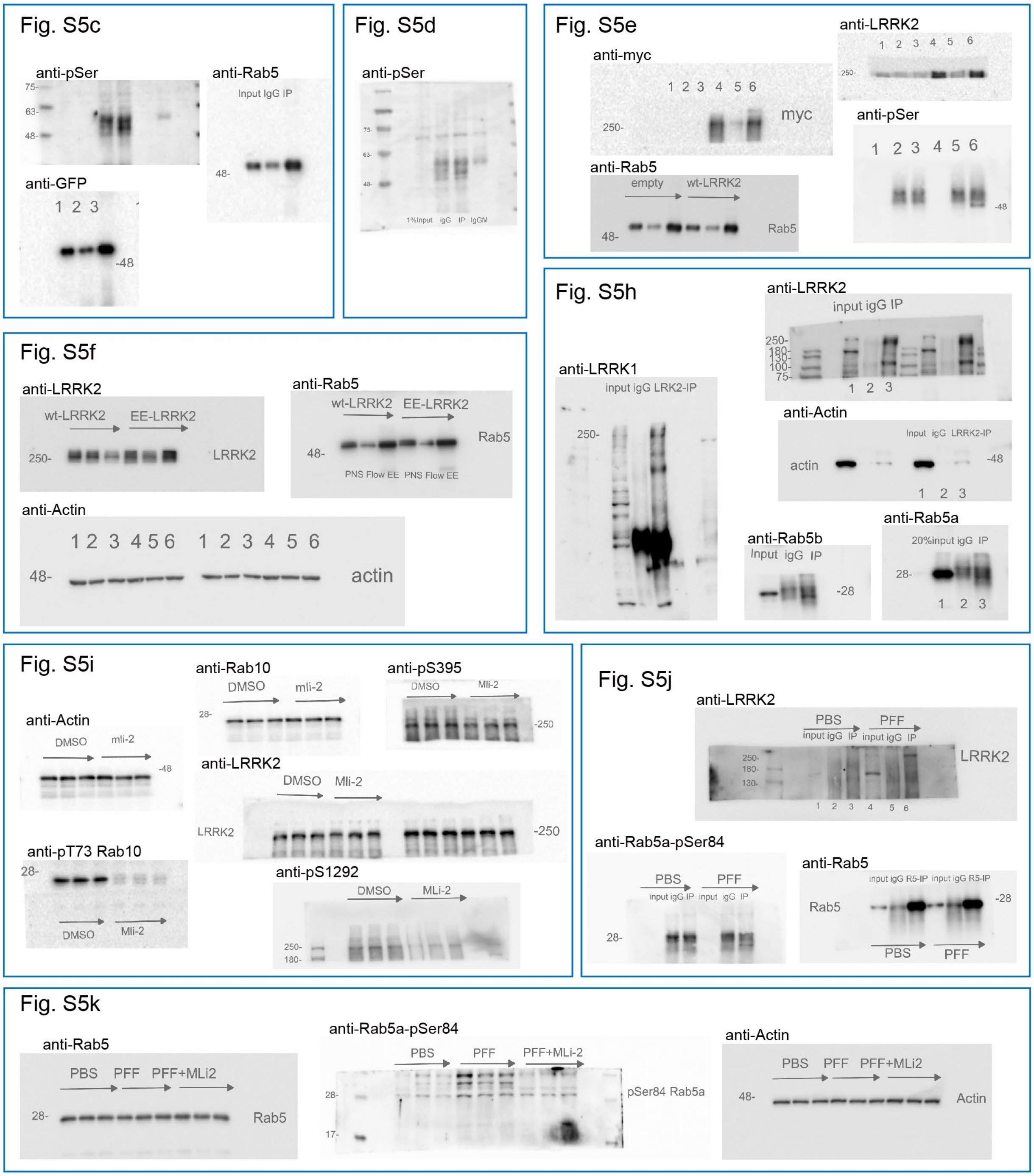

Fig. S5l

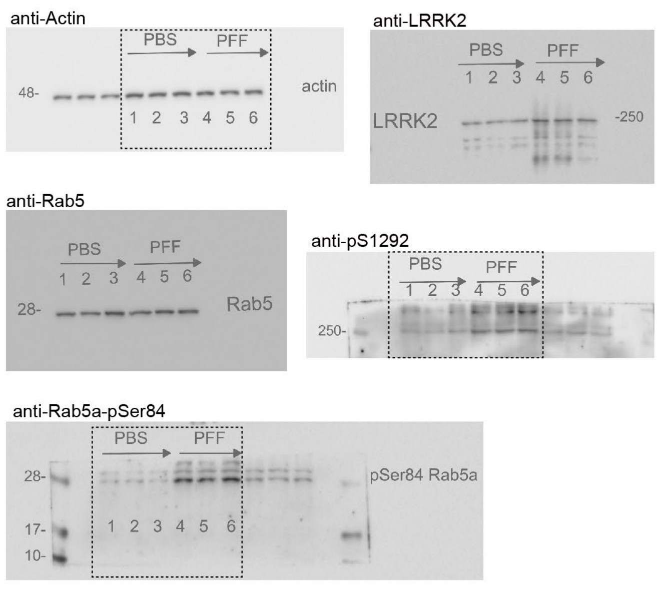

Fig. S5m

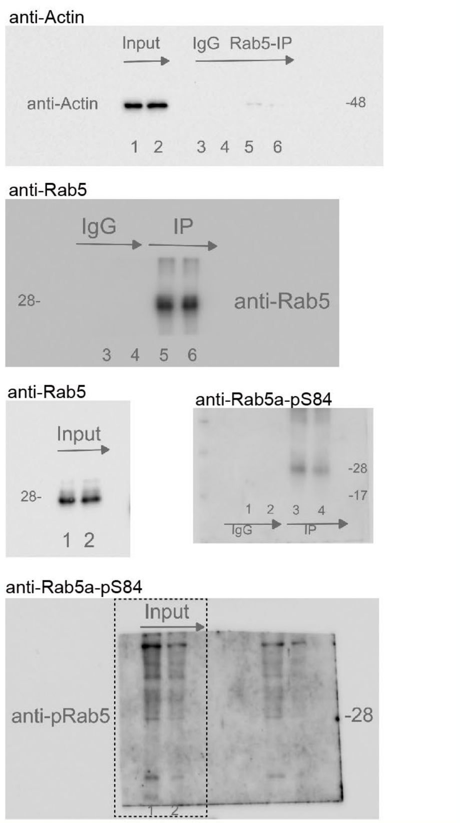

Fig. S5n

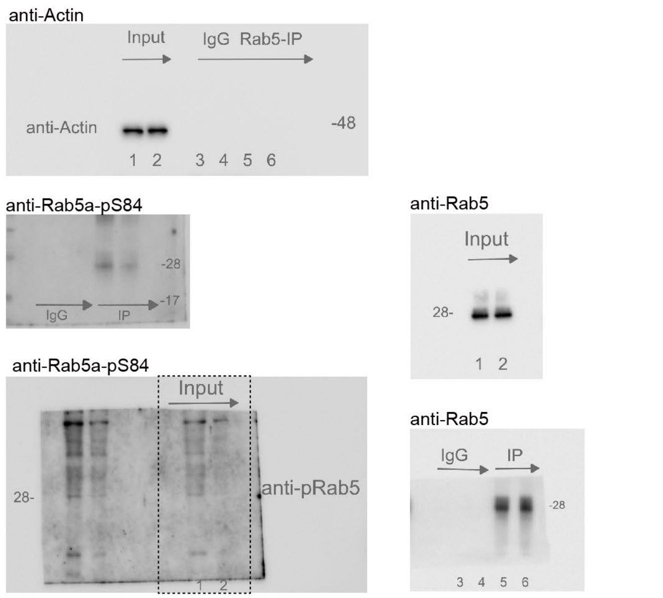

Fig. S5v

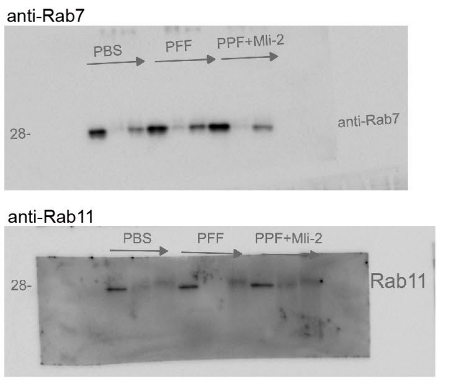

Supplementary Fig. 6

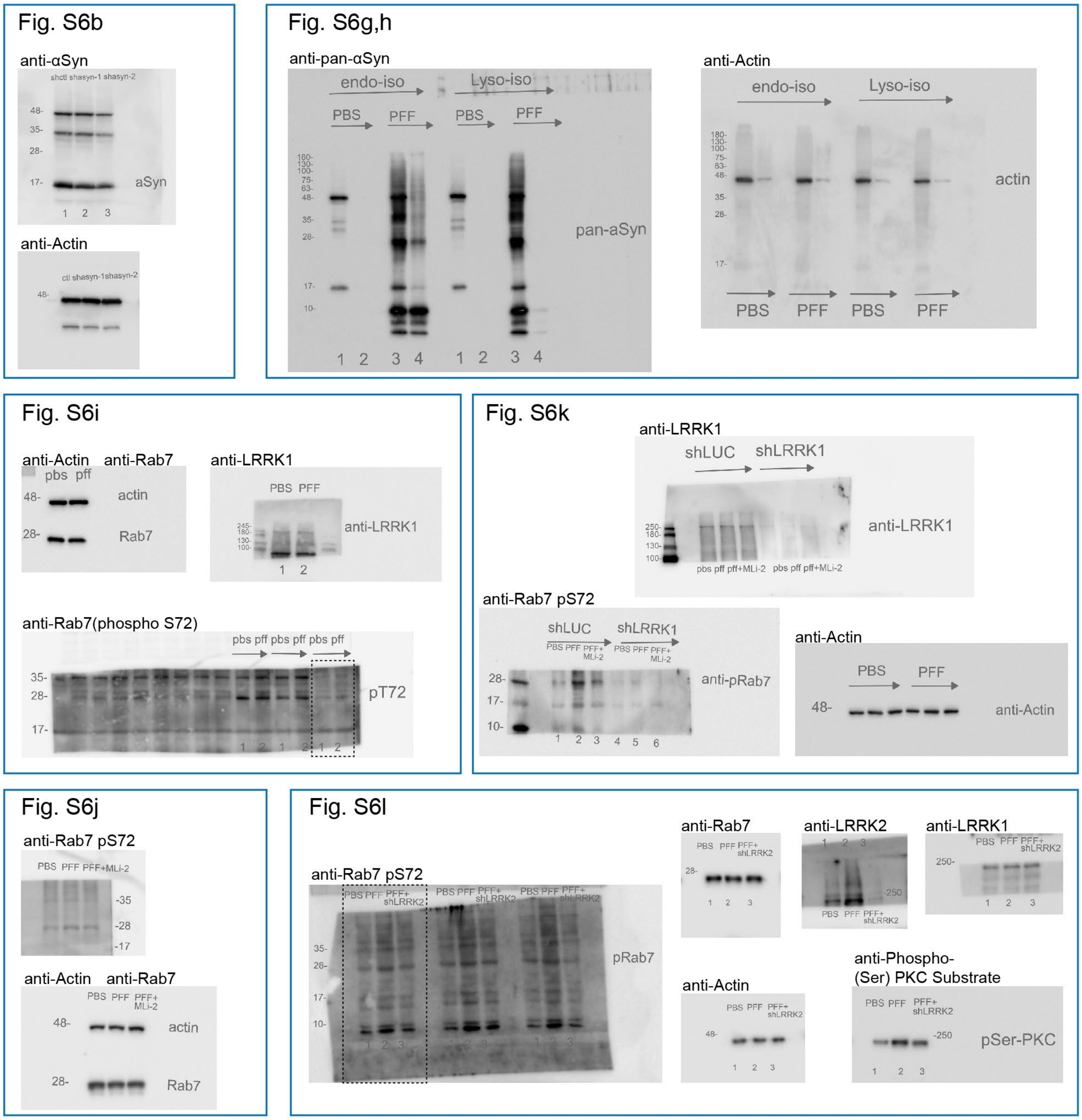

Supplementary Fig. 7

Fig. S7c

anti-Rab5a anti-Actin

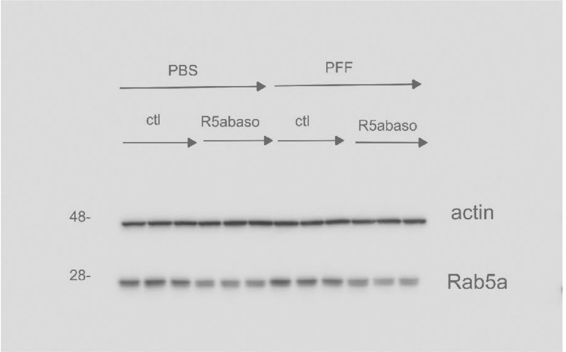

anti-Rab5b anti-Actin

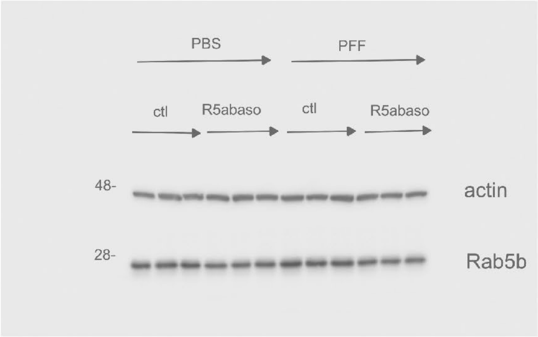

Supplementary Fig. 8

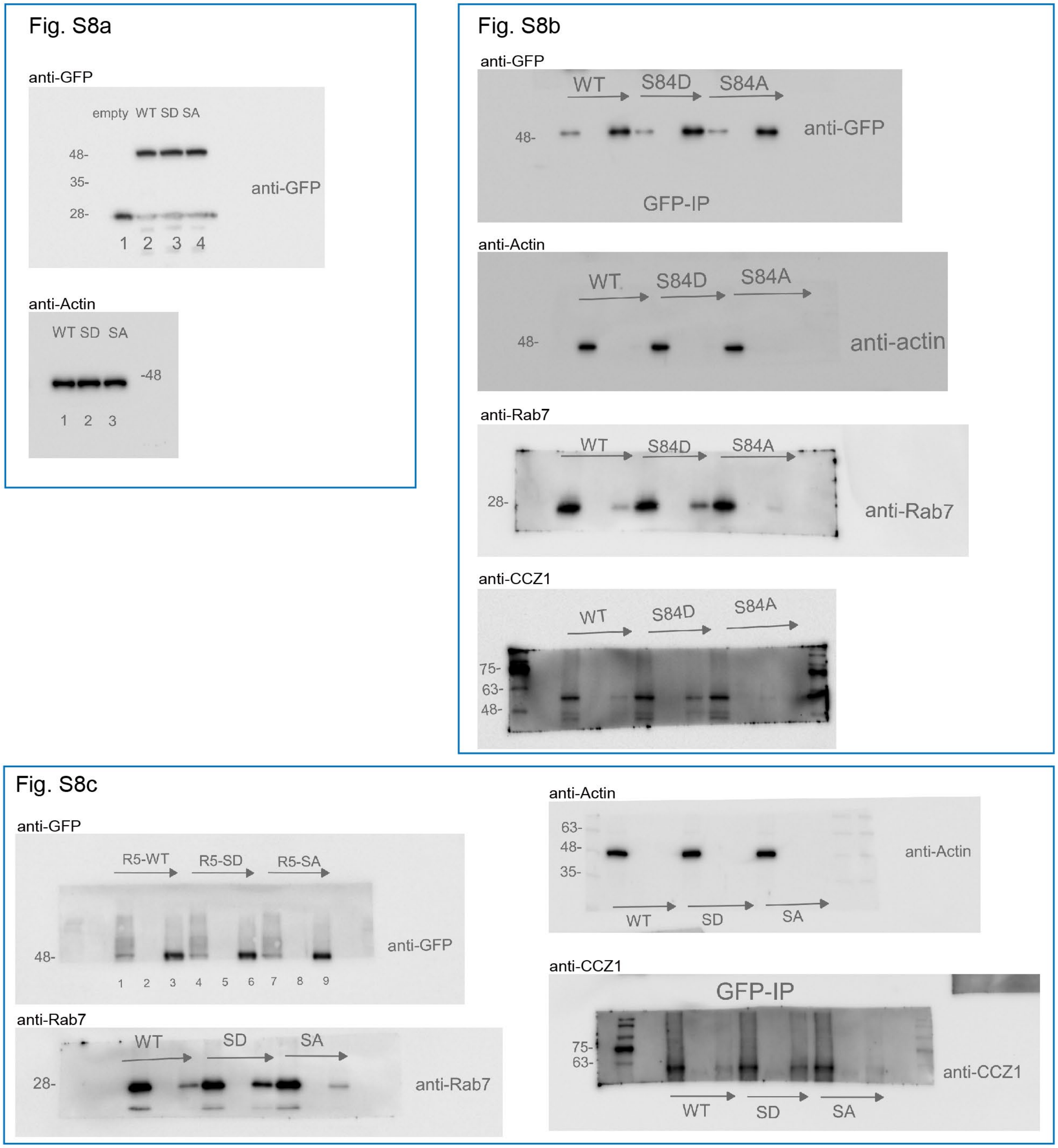

The ARRIVE Essential 10: Compliance Questionnaire

Use this questionnaire to evaluate how well a manuscript complies with the ARRIVE Essential 10. It can be applied to any manuscript describing comparative experiments in living animals, by assessors such as journal staff, editors, or peer reviewers.

Item	Question(s)	Answers
1 Study Design	Are all experimental and control groups clearly identified?	☒ Yes, for at least one experiment<br>☐ No
	Is the experimental unit (e.g. an animal, litter or cage of animals) clearly identified?	☒ Yes, for at least one experiment<br>☐ No
2 Sample Size	Is the exact number of experimental units in each group at the start of the study provided (e.g. in the format '*n*=')?	☒ Yes, for at least one experiment<br>☐ No
	Is the method by which the sample size was chosen explained?	☒ Yes, for at least one experiment<br>☐ No
3 Inclusion & Exclusion Criteria	Are the criteria used for including and excluding animals, experimental units, or data points provided?	☐ Yes, for at least one experiment<br>☒ No
	Are any exclusions of animals, experimental units, or data points reported, or is there a statement indicating that there were no exclusions?	☒ Yes, for at least one analysis<br>☐ No
4 Randomisation	Is the method by which experimental units were allocated to control and treatment groups described?	☒ Yes, for at least one experiment<br>☐ No
5 Blinding	Is it clear whether researchers were aware of, or blinded to, the group allocation at any stage of the experiment or data analysis?	☒ Yes, for at least one experiment<br>☐ No
6 Outcome Measures	For all experimental outcomes presented, are details provided of exactly what parameter was measured?	☒ Yes, for at least one experiment<br>☐ No
7 Statistical Methods	Is the statistical approach used to analyse each outcome detailed?	☒ Yes, for at least one analysis<br>☐ No
	Is there a description of any methods used to assess whether data met statistical assumptions?	☒ Yes, for at least one analysis<br>☐ No<br>☐ Not applicable
8 Experimental Animals	Are all species of animal used specified?	☒ Yes, for at least one experiment<br>☐ No
	Is the sex of the animals specified?	☐ Yes, for at least one experiment<br>☒ No<br>☐ Not applicable to species
	Is at least one of age, weight or developmental stage of the animals specified?	☒ Yes, for at least one experiment<br>☐ No
9 Experimental Procedures	Are both the timing and frequency with which procedures took place specified?	☒ Yes, for at least one experiment<br>☐ No
	Are details of acclimatisation periods to experimental locations provided?	☐ Yes, for at least one experiment<br>☒ No
10 Results	Are descriptive statistics for each experimental group provided, with a measure of variability (e.g. mean and SD, or median and range)?	☒ Yes, for at least one experiment<br>☐ No<br>☐ Not applicable to the type of data collected
	Is the effect size and confidence interval provided?	☐ Yes, for at least one experiment<br>☐ No<br>☒ Not applicable to the type of analysis used

Notes on questionnaire design

The ARRIVE guidelines are a useful resource for authors preparing manuscripts describing animal research, and also provide a framework to evaluate the transparency of those manuscripts. To assess reporting quality, numerous studies have in the past sought to operationalise reporting guidelines (including ARRIVE). Typically, this involves scoring a manuscript's degree of compliance with guideline items in a binary fashion (e.g. an item is either not reported or reported) [1-3], a graded fashion (e.g. not, partially, or completely reported) [4,5], or a combination of the two [6].

This questionnaire has been designed to be as concise and user-friendly as possible. The number of questions used to assess a manuscript's compliance has been kept to a minimum, and in most cases each question is designed to be answered in a binary fashion. Compliance with some Essential 10 sub-items is inherently impossible to judge in this way, instead requiring a subjective judgement on the level of detail provided. For this reason, not all sub-items are represented by a question in this questionnaire.

To facilitate binary answers, it has been necessary to identify the minimum information in a manuscript sufficient to comply with each question. The strengths of this approach include the relatively short length of the questionnaire (and the correspondingly low time burden of using it), and the avoidance of ambiguity that would arise from a graded answering system, in which an intermediate score (e.g. 'partially/insufficiently reported') could denote a number of distinct deficiencies in compliance with an item (e.g. either only part of the item was complied with, or only the reporting of some experiments in the manuscript complied with the item.)

Limitations of this approach centre on the necessity to identify the minimum information sufficient to comply with each question. In some cases, this has resulted in questions that require a guideline sub-item's criteria to have been fulfilled in the reporting of only one experiment in a manuscript. As a result, not all experiments in a manuscript may be described in a way that fulfils that criterion, despite the manuscript being considered to comply with the guidelines overall.

References

1. Hair *et al* (2020). *Res Integ Peer Rev*. doi: 10.1186/s41073-019-0069-3
2. Tihanyi *et al* (2019). *J Surg Res*. doi: 10.1016/j.jss.2018.10.038
3. Zhao *et al* (2020). *BMC Vet Res*. doi: 10.1186/s12917-020-02664-1
4. Han *et al* (2017). *Plos One*. doi: 10.1371/journal.pone.0183591
5. Chatzimanouil *et al* (2019). *J Am Soc Nephrol*. doi: 10.1681/ASN.2018050515
6. Leung *et al* (2018). *Plos One*. doi: 10.1371/journal.pone.0197882
